# A Wars2 Mutant Mouse Model Displays OXPHOS Deficiencies and Activation of Tissue-Specific Stress Response Pathways

**DOI:** 10.1016/j.celrep.2018.11.080

**Published:** 2018-12-18

**Authors:** Thomas Agnew, Michelle Goldsworthy, Carlos Aguilar, Anna Morgan, Michelle Simon, Helen Hilton, Chris Esapa, Yixing Wu, Heather Cater, Liz Bentley, Cheryl Scudamore, Joanna Poulton, Karl J. Morten, Kyle Thompson, Langping He, Steve D.M. Brown, Robert W. Taylor, Michael R. Bowl, Roger D. Cox

**Affiliations:** 1 MRC Harwell Institute, Mammalian Genetics Unit and Mary Lyon Centre, Harwell Campus, Oxfordshire OX11 0RD, UK; 2 Nuffield Department of Obstetrics and Gynaecology, University of Oxford, Level 3 The Women’s Centre, John Radcliffe Hospital, Headington, Oxford OX3 9DU, UK; 3 Wellcome Centre for Mitochondrial Research, Institute of Neuroscience, The Medical School, Newcastle University, Newcastle upon Tyne NE2 4HH, UK

**Keywords:** WARS2, deafness, adiposity, hypertrophic cardiomyopathy, pleiotropic, ISR, mitochondrial dysfunction

## Abstract

Mutations in genes essential for mitochondrial function have pleiotropic effects. The mechanisms underlying these traits yield insights into metabolic homeostasis and potential therapies. Here we report the characterization of a mouse model harboring a mutation in the tryptophanyl-tRNA synthetase 2 (*Wars2*) gene, encoding the mitochondrial-localized WARS2 protein. This hypomorphic allele causes progressive tissue-specific pathologies, including hearing loss, reduced adiposity, adipose tissue dysfunction, and hypertrophic cardiomyopathy. We demonstrate the tissue heterogeneity arises as a result of variable activation of the integrated stress response (ISR) pathway and the ability of certain tissues to respond to impaired mitochondrial translation. Many of the systemic metabolic effects are likely mediated through elevated fibroblast growth factor 21 (FGF21) following activation of the ISR in certain tissues. These findings demonstrate the potential pleiotropy associated with *Wars2* mutations in patients.

## Highlights

• A hypomorphic point mutation in the *Wars2* gene was identified
• Mutant mice exhibit progressive tissue-specific pathologies
• Variable activation of stress response pathways
• Demonstrating pleiotropic effects

## Graphical Abstract

**Figure:**
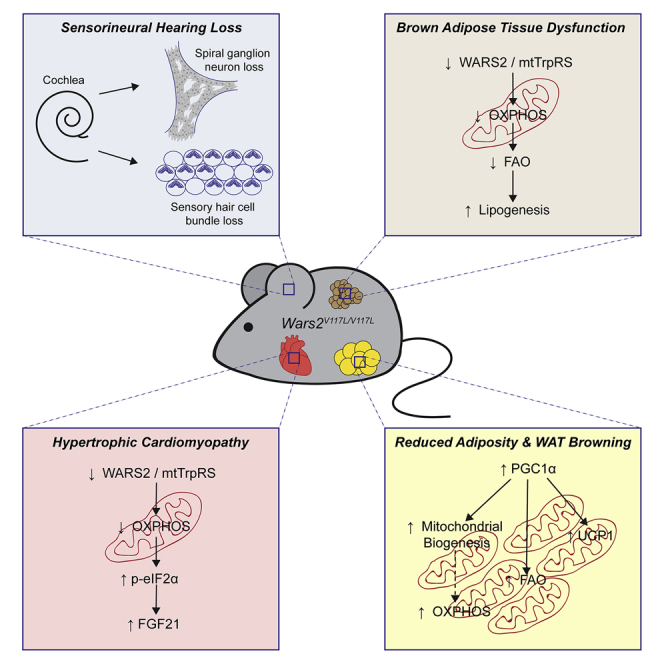

## Teaser

A reduced-function mutation in the nuclear-encoded, mitochondrial-localized *Wars2* gives rise to deafness, reduced and abnormal fat, and hypertrophic cardiomyopathy. Agnew et al. show that the different tissue effects of this mutation arise from variable activation of stress response pathways and tissue-specific responses to impaired mitochondrial function.

## Introduction

Mitochondrial diseases are a heterogeneous group of disorders caused by mutations in mitochondrial proteins encoded by either the mitochondrial genome (mtDNA) or the nuclear genome (genomic DNA [gDNA]). The nuclear-encoded mitochondrial aminoacyl-tRNA synthetase (mt-aaRS) proteins catalyze the aminoacylation of mitochondrial tRNAs with their cognate amino acid. Mitochondrial tRNA aminoacylation is fundamental to mitochondrial translation and synthesis of mtDNA-encoded respiratory chain subunits and the supply of ATP to the cell. The mt-aaRS proteins are encoded by separate nuclear genes with the exception of glycine- and lysine-tRNA synthetase (*GARS* and *KARS*), which function in both the mitochondria and the cytoplasm. With the description of families with compound heterozygous variants in the tryptophanyl-tRNA synthetase 2 (*WARS2*) gene (Burke et al., 2018, Musante et al., 2017, Theisen et al., 2017, Vantroys et al., 2018, Wortmann et al., 2017), patients have been reported with biallelic, pathogenic mutations in all 19 nuclear-encoded mt-aaRS genes (Oprescu et al., 2017).

Surprisingly, given their common function within mitochondrial translation and ubiquitous expression, mt-aaRS mutations cause distinct tissue-specific pathologies and respiratory chain deficiencies in a gene-dependent manner (Konovalova and Tyynismaa, 2013). For example, pathogenic histidyl-tRNA synthetase 2 (*HARS2*) (Pierce et al., 2011) and leucyl-tRNA synthetase 2 (*LARS2*) (Soldà et al., 2016) mutations cause Perrault syndrome (sensorineural hearing loss and ovarian dysgenesis), glutamyl-tRNA synthetase 2 (*EARS2*) (Steenweg et al., 2012) mutations cause leukoencephalopathy with thalamus and brainstem involvement with high lactate (LTBL), and seryl-tRNA synthetase 2 (*SARS2*) (Belostotsky et al., 2011, Rivera et al., 2013) mutations cause hyperuricemia, pulmonary hypertension, renal failure, and alkalosis (HUPRA) syndrome with hypertrophic cardiomyopathy. The underlying mechanisms dictating the pleiotropic effects and tissue-specific penetrance and variability among individuals with mt-aaRS mutations are unknown and are a major challenge in the understanding and developing therapies for mitochondrial disease (Nunnari and Suomalainen, 2012).

Global mt-aaRS knockout animal models are heterozygous haploinsufficient and homozygous lethal (http://www.mousephenotype.org/) (Dickinson et al., 2016). A heart and skeletal muscle-specific aspartyl-tRNA synthetase 2 (*Dars2*) knockout (Dars2-KO^Ckmm^) mouse with fatal cardiomyopathy has been reported (Dogan et al., 2014). Complete loss of *Dars2* function caused disrupted mitochondrial proteostasis and activating transcription factor 4 (ATF4)-dependent fibroblast growth factor 21 (FGF21) expression specifically in the heart, but not in skeletal muscle, suggesting tissue-specific differences in mitochondrial proteostatic buffering capacity. However, residual mt-aaRS activity is retained in human patients with mt-aaRS mutations; thus, animal models with global-hypomorphic mt-aaRS alleles are vital to investigating tissue-specific penetrance.

Hypomorphs from *N*-ethyl-*N*-nitrosourea (ENU) mutagenesis screens in the mouse allow the pleiotropic effects of a mutation to be identified. We have incorporated aging as a sensitizing factor to assess recessive pedigrees for late-onset and progressive phenotypes in metabolism and other body systems (Potter et al., 2016). We report here the identification of an ENU-induced mouse mutant harboring a recessive hypomorphic point mutation in the *Wars2* gene, *Wars2*^*V117L*^, which causes a complex tissue-specific pathology, including hearing loss, reduced adiposity, adipose tissue dysfunction, and hypertrophic cardiomyopathy. We demonstrate that reduced WARS2 levels causes tissue-specific respiratory chain deficiencies, modeling human mt-aaRS patients. We demonstrate that tissue-specific upregulation of mitochondrial biogenesis is coincident with respiratory chain deficiencies in *Wars2*^*V117L/V117L*^ mice, likely contributing to the tissue-specific respiratory chain deficiencies observed. We also show that activation of the integrated stress response (ISR) is a heart-specific response to inhibition of mitochondrial translation that contributes to increased FGF21 levels and systemic changes in metabolism.

## Results

We applied high-throughput broad-based phenotyping to pedigrees of mutagenized mice to investigate the pleiotropic effects of the mutations identified (Potter et al., 2016).

### *Wars2*-V117L ENU-Induced Mutation Causal for Hearing Loss and Reduced Adiposity

Auditory phenotyping of one of these pedigrees (MPC151) identified progressive hearing loss. At 3 months of age, all mice displayed a normal response to a clickbox stimulus. However, at 6 months of age, 2 of 58 mice had a reduced response, increasing to 7 animals (12%) by 9 months of age. At 12 months of age, the pedigree was assessed using auditory brainstem response (ABR) testing, which showed that 5 of the 53 surviving mice exhibited elevated hearing thresholds at all frequencies tested (Figure S1A). In addition, the hearing-impaired mice were found to have reduced body weight (Figure S1B).

A genome scan of G_3_ mice showed linkage to a ∼73.3 Mb region on chromosome 3 containing 1,298 genes (Figure S1C). DNA from an affected G_3_ mouse (MPC151/2.10 g) underwent whole-genome sequencing, and analysis of the data identified only three high-confidence non-synonymous coding changes within the mapped interval. These consisted of Chr3:93446568A>T at nucleotide 3314 of the trichohyalin (*Tchh*) gene (Ensembl: ENSMUST00000064257), causing an aspartate-to-valine substitution at residue 1105 (*Tchh*^*D1105V*^); Chr3:99204536G>T at nucleotide 349 of the *Wars2* gene (Ensembl: ENSMUST00000004343), causing a valine-to-leucine substitution at residue 117 (*Wars2*^*V117L*^); and Chr3:133330454A>T at nucleotide 368 of the pyrophosphatase (inorganic) 2 (*Ppa2*) gene (Ensembl: ENSMUST00000029644), causing a tyrosine-to-phenylalanine substitution at residue 123 (*Ppa2*^*Y123F*^). The presence of the three lesions was confirmed using Sanger sequencing, and only mice showing hearing impairment were homozygous for these ENU-induced lesions (Figure S1D).

To segregate the mutations, the offspring were backcrossed for three generations to C3H.Pde6b+ mice. The *Ppa2*^*Y123F*^ allele was segregated from the *Tchh*^*D1105V*^ and *Wars2*^*V117L*^ alleles at backcross 2. However, the *Tchh*^*D1105V*^ and *Wars2*^*V117L*^ alleles remained linked due to their proximity. Auditory phenotyping of *Ppa2*^*Y123F/Y123F*^ mice at 6 months of age showed they had similar ABR thresholds to their wild-type and heterozygous littermates (Figure S1E, A). In addition, the body, fat, and lean mass of animals for each genotype were not significantly different (Figure S1E, B–G). Thus, the *Ppa2*^*Y123F*^ lesion was excluded as being causative of the phenotypes.

The *Tchh* gene encodes a protein for hair shaft formation, and a patient with a homozygous nonsense *TCHH* mutation and uncombable hair syndrome has been described (Ü Basmanav et al., 2016). We did not observe a hair phenotype in *Wars2*^*V117L/V117L*^ mice. However, to determine which of the two lesions, *Wars2*^*V117L*^ or *Tchh*^*D1105V*^, is causal, we undertook a genetic complementation test, crossing *Wars2*^*V117L/+*^:*Tchh*^*D1105V/+*^ mice with mice heterozygous for a *Wars2* knockout (*Wars2*^+/−^) allele (Figure S2A). This generated offspring that are compound heterozygotes for *Wars2*, but heterozygous for *Tchh* (*Wars2*^*V117L/*−^:*Tchh*^*D1105V/+*^). These mice displayed elevated ABR thresholds at 4 months of age and reduced weight, total fat, and lean mass compared to their colony mates, which had normal hearing and weight (*Wars2*^+/+^:*Tchh*^+/+^, *Wars2*^*V117L/+*^*:Tchh*^*D1105V/+*^, and *Wars2*^+/−^:*Tchh*^+/+^) (Figures S2B–S2E). Failure of the *Wars2* alleles to complement confirms the *Wars2*^*V117L*^ lesion as the causal mutation underlying the observed phenotypes. Homozygous null (*Wars2*^−/−^) mice were embryonic lethal, and *Wars2*^*V117L/*−^ and *Wars2*^*V117L/V117L*^ mice were subviable and viable, respectively; thus, the *Wars2*^*V117L*^ allele is hypomorphic, rather than a complete loss of function (Table S1).

To further characterize the phenotypes and establish underlying mechanisms, we bred additional cohorts of mice.

### Hearing Loss in *Wars2*^*V117L/V117L*^ Mice Was Progressive

**Figure 1:**
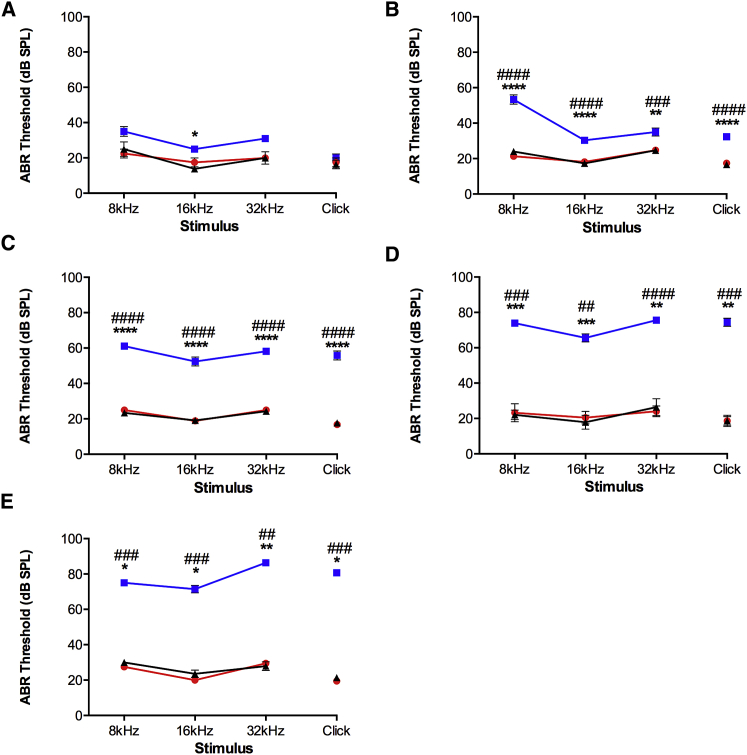
ABR Phenotyping Minimum auditory detection thresholds (decibel sound pressure level, dB SPL) were determined using auditory brainstem response (ABR) at (A) 1 month, (B) 3 months, (C) 6 months, (D) 10 months, and (E) 12 months of age. *Wars2*^*V117L/V117L*^, *Wars2*^*V117L/+*^, and *Wars2*^+/+^ littermate numbers (males and females pooled) were (A) 5, 2, and 4; (B) 15, 19, and 15; (C) 19, 19, and 22; (D) 9, 11, and 7; and (E) 7, 10, and 7, respectively; mean ± SEM. Significance was determined using a one-way ANOVA Kruskal-Wallis test with Dunn’s multiple comparisons test. Significance between *Wars2*^*V117L/V117L*^ and *Wars2*^+/+^ and between *Wars2*^*+/V117L*^ and *Wars2*^+/+^ is shown as ^∗^ and #p < 0.05, ^∗∗^ and ##p < 0.01, ^∗∗∗^ and ###p < 0.001, and ^∗∗∗∗^ and ####p < 0.0001, respectively. *Wars2*^*V117L/V117L*^ mice are blue squares, *Wars2*^*V117L/+*^ mice are red circles, and *Wars2*^+/+^ mice are black triangles. See also Figures S1–S3 and Table S1.

To investigate progression of the auditory phenotype, ABR was measured at 1, 3, 6, 10, and 12 months of age. The hearing thresholds of *Wars2*^+/+^ and *Wars2*^*+/V117L*^ mice were comparable and within the normal range at all ages tested. In contrast, *Wars2*^*V117L/V117L*^ mice display an age-related increase in hearing thresholds at all tested frequencies (Figure 1). Investigation of the cochlear sensory epithelia using scanning electron microscopy showed a progressive loss of outer hair cell stereocilia bundles in the homozygous mutants, with an apical-to-basal increase in severity (Figure S3A). In addition, assessment of cochlear histological sections identified a reduced number of spiral ganglion neurons in the cochlear apex of 12-month-old mutant mice (Figure S3B). The mutant mice showed no overt vestibular dysfunction (e.g., circling, head bob, or abnormal swim), and no craniofacial dysmorphology was observed.

### *Wars2*^*V117L/V117L*^ Mice Failed to Gain Fat Mass

**Figure 2:**
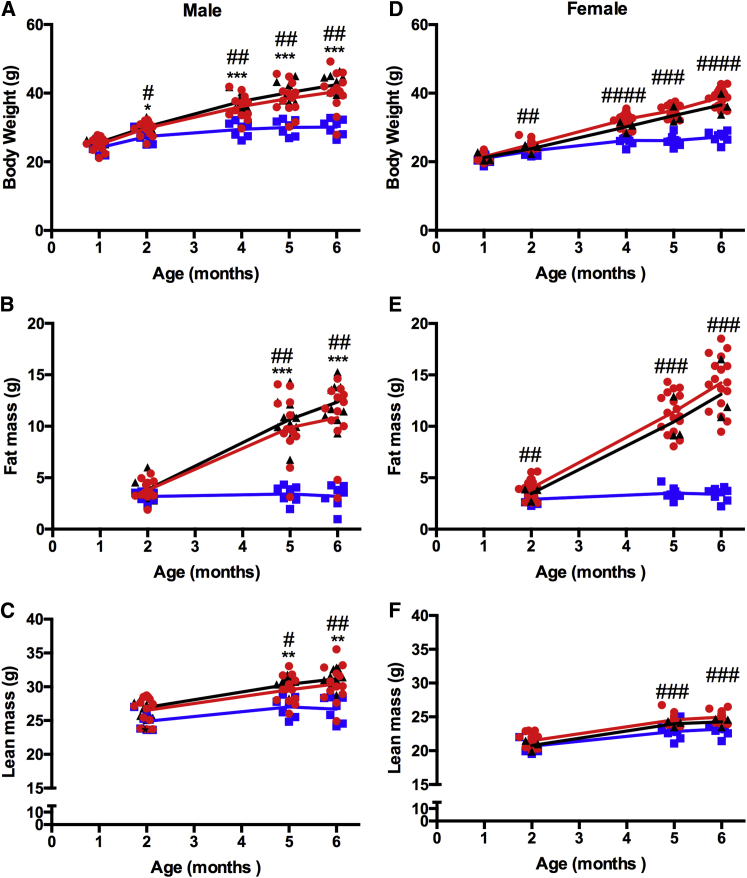
*Wars2*^*V117L/V117L*^ Mice Have Reduced Body Weight due to Reduced Adiposity Male and female cohort 1 mice: (A and D) body weight, (B and E) fat mass, and (C and F) lean mass, respectively. *Wars2*^*V117L/V117L*^, *Wars2*^*V117L/+*^, and *Wars2*^+/+^ littermate numbers were 7 and 8, 13 and 17–21, and 11 and 3 male and female, respectively; mean ± SD. Areas under the curve (AUCs) were compared for males using an ordinary one-way ANOVA with Tukey’s multiple comparison test and for females using a one-way ANOVA non-parametric Kruskal-Wallis test and Dunn’s multiple comparison test. For AUC for *Wars2*^+/+^ and *Wars2*^*V117L/V117L*^, *Wars2*^*+/V117L*^ and *Wars2*^*V117L/V117L*^, and *Wars2*^+/+^ and *Wars2*^*V117L/+*^, male body weight was p < 0.0001, p = 0.0002, and p > 0.5057; fat mass was p < 0.0001, p < 0.0001, and p = 0.6505; and lean mass was p = 0.0063, p = 0.0008, and p = 0.5314. Female body weight for *Wars2*^*+/V117L*^ and *Wars2*^*V117L/V117L*^ was p < 0.0001, fat mass was p = 0.0001, and lean mass was p = 0.0055 (wild-type [WT] comparisons not shown, because n = 3). Significance at specific time points was calculated with a one-way ANOVA non-parametric Kruskal-Wallis test and Dunn’s multiple comparison test. Significance between *Wars2*^+/+^ and *Wars2*^*V117L/V117L*^ and between *Wars2*^*+/V117L*^ and *Wars2*^*V117L/V117L*^ is shown as ^∗^ and #p < 0.05, ^∗∗^ and ##p < 0.01, and ^∗∗∗^ and ###p < 0.001, respectively. *Wars2*^*V117L/V117L*^ mice are blue squares, *Wars2*^*V117L/+*^ mice are red circles, and *Wars2*^+/+^ mice are black triangles. See also Figure S4.

**Figure 3:**
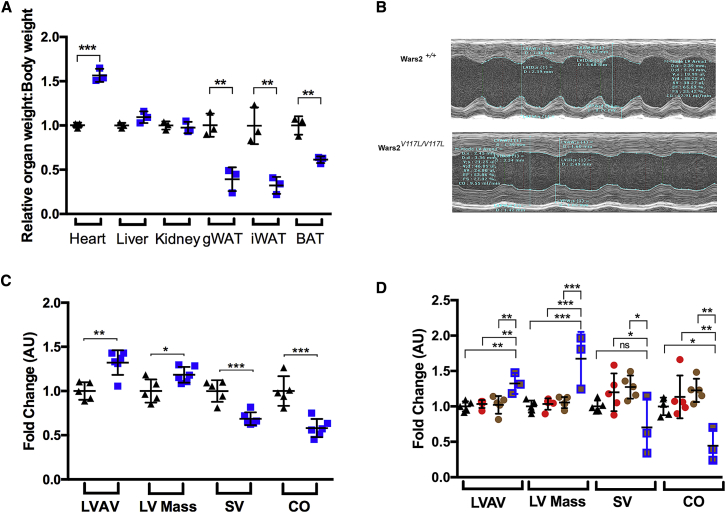
The *Wars2*^*V117L*^ Allele Causes Hypertrophic Cardiomyopathy (A) Organ weight divided by body weight at 6 months of age in male mice. *Wars2*^*V117L/V117L*^ and *Wars2*^+/+^ animal numbers were 3 and 3, respectively; mean ± SD. Data were analyzed with multiple t tests by the Holm-Sidak method. (B) Representative echocardiogram images of the left ventricle in male *Wars2*^*V117L/V117L*^ and *Wars2*^+/+^ mice at 6 months of age. (C) Functional analysis of images for left ventricle anterior wall (LVAW) diameter, left ventricle (LV) mass, stroke volume (SV), and cardiac output (CO). *Wars2*^*V117L/V117L*^ and *Wars2*^+/+^ male mice animal numbers were 6 and 5, respectively; mean ± SD. Data were analyzed using an ordinary one-way ANOVA with Tukey’s post hoc test to correct for multiple comparisons. (D) Echocardiogram analysis in *Wars2*^*V117L/*−^ male mice at 6 months of age. *Wars2*^*V117L/*−^, *Wars2*^*V117L/+*^, *Wars2*^+/−^ and *Wars2*^+/+^ animal numbers were 3, 5, 5, and 5, respectively; mean ± SD. Data were analyzed using an ordinary one-way ANOVA with Tukey’s post hoc test to correct for multiple comparisons. ^∗^p < 0.05, ^∗∗^p < 0.01, ^∗∗∗^p < 0.001. *Wars2*^*V117L/V117L*^ mice are blue squares, *Wars2*^*+/+*^ mice are black triangles, *Wars2*^*V117L/+*^ mice are red filled circles, compound heterozygote *Wars2*^*V117L/*−^ mice are brown filled blue squares, and *Wars2*^+/−^ mice are brown circles.

To refine the reduced body weight phenotype, we analyzed body composition at monthly intervals and found reduced total body weight from 2 months of age, reduced fat mass from 2 months (female) or 3 months (male) of age, and lean mass from 3 months (male) or 5 months (female, in cohort 1 only) of age (Figures 2A–2C, male; Figures 2D–2F, female cohort 1; Figures S4A–S4C, male; Figures S4D–S4F, female cohort 2). Thus, demonstration of the reduction in total mass was primarily due to decreased adiposity and a failure to increase fat mass. We further investigated whether these differences were the result of specific organ weight changes in *Wars2*^*V117L/V117L*^ mice dissected at 6 months of age. Visceral gonadal white adipose tissue (gWAT), subcutaneous inguinal WAT (iWAT), and brown adipose tissue (BAT) normalized to body weight were all significantly reduced in *Wars2*^*V117L/V117L*^ mice compared to wild-type colony mates (Figure 3A), consistent with reduced adiposity. Strikingly, heart weight was increased in *Wars2*^*V117L/V117L*^ mice (Figure 3A). No significant differences in liver or kidney weight (Figure 3A) were observed, demonstrating organ specificity and that the changes in adipose tissues and heart weight were not because of global growth or development impairment.

### *Wars2*^*V117L/V117L*^ Mice Showed Hypertrophic Cardiomyopathy

To determine the cause of increased heart weight, *Wars2*^*V117L/V117L*^ cardiac morphology was assessed by echocardiogram at 5 months of age (Figure 3B). We found significantly increased left ventricular anterior wall (LVAW) diameter and left ventricular (LV) mass in *Wars2*^*V117L/V117L*^ mice relative to wild-type colony mates, showing that the increase in heart weight was due to hypertrophic cardiomyopathy (Figure 3C). Consistent with this, the LV stroke volume (SV) and cardiac output (CO) were significantly reduced (Figure 3C). These differences were also observed in compound heterozygote *Wars2*^*V117L/*−^ mice, which showed increased LVAW and LV mass and decreased CO relative to *Wars2*^+/+^, *Wars2*^*+/V117L*^, and *Wars2*^+/−^ mice at the same age, regardless of *Tchh* genotype (Figure 3D), confirming that the *Wars2*^*V117L*^ allele was the causal mutation for hypertrophic cardiomyopathy.

### *Wars2*^*V117L/V117L*^ Mice Did Not Show Gross Brain Pathology

We carried out additional pathology screens to investigate whether there were neurological abnormalities, as reported in patients. On light microscopic examination of the brain (multiple sections of cerebrum and cerebellum) (data not shown) there were no detectable morphological differences between homozygote and wild-type animals (n = 3 of each) of the same age (approximately 7 months) and sex (male). In particular, there was no evidence of myelin deficits. There was also no evidence from visual welfare observation of an *in vivo* neurological phenotype (seizures, tremors, or changes in locomotion), the detection of which often precedes detectable morphological changes at the light-microscopy level. Detection of potential subtle neurological changes would require behavioral or neurophysiological testing, which was beyond the scope of this study.

### The *Wars2* c.349G>T Mutation Disrupted Exon Splicing and Caused Tissue-Specific WARS2 Deficiencies

**Figure 4:**
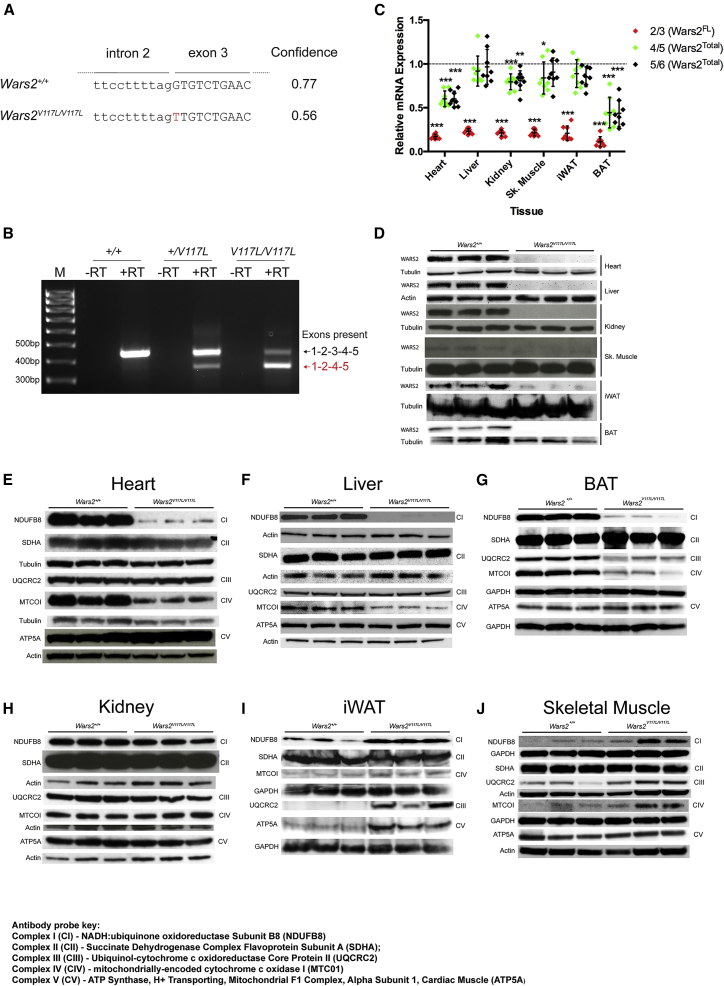
*Wars2*^*V117L*^ Allele Increases Exon Skipping, Causing Tissue-Specific *WARS2* and Mitochondrial Respiratory Chain Deficiencies (A) *Wars2* c.349G > T NetGene2 splice site prediction. (B) RT-PCR of RNA extracted from cochleae of *Wars2*^+/+^, *Wars2*^*V117L/+*^, and *Wars2*^*V117L/V117L*^ mice using oligonucleotide primer pairs designed to exons 1 and 5. Products were sequenced and contain the exons indicated. (C) To quantify *Wars2* mRNA missplicing *in vivo*, RNA was extracted from tissues from mice at 12 months of age and qRT-PCR was performed using 3 TaqMan probes targeted to alternate *Wars2* exon-exon boundaries and expressed relative to *Wars2*^+/+^: boundaries 2/3 (Wars2^FL^), 4/5 (Wars2^Total^), and 5/6 (*Wars2*^Total^). *Wars2*^*V117L/V117L*^ and *Wars2*^+/+^ littermate numbers were 8 and 8 (5 male and 3 female), respectively; mean ± SEM. Data were analyzed using a Mann-Whitney two-tailed t test. Significance differences between *Wars2*^*V117L/V117L*^ and *Wars2*^+/+^ samples are shown as ^∗^p < 0.05, ^∗∗^p < 0.01, and ^∗∗∗^p < 0.001. (D–J) Immunoblot analysis of (D) WARS2 protein levels in multiple tissues and (E–J) mitochondrial respiratory chain subunit protein levels in (E) heart, (F) liver, (G) brown adipose tissue (BAT), (H) kidney, (I) inguinal white adipose tissue (iWAT), and (J) skeletal muscle from female mice at 12 months of age. *Wars2*^*V117L/V117L*^ and *Wars2*^+/+^ littermate numbers were 3 and 3, respectively. See also Figure S5.

The *Wars2* c.349G>T lesion causes a missense substitution (p.V117L) in the encoded protein. *In silico* prediction of the functional effects of the p.V117L missense substitution did not predict that it was deleterious. However, the mutated nucleotide is the first coding nucleotide of exon 3, and the NetGene2 splice site prediction program indicated that substitution of G to T at the first nucleotide of the third exon of *Wars2* would affect the efficiency of exon 3 splicing (Figure 4A) (Hebsgaard et al., 1996). We modeled the predicted consequence of exon 3 skipping and found that three α helices, required for substrate binding and release, are missing, which would likely lead to a loss of WARS2 function (Figure S1F). To test the prediction of exon skipping *in vivo*, RT-PCR analysis of cochlear RNA derived from wild-type, heterozygous mutant, and homozygous mutant mice was undertaken (Figure 4B). This showed the *Wars2*^*V117L*^ allele, c.349G > T, caused in-frame skipping of exon 3. However, the mutation does not abolish normal splicing, and some full-length transcript is still produced (Figure 4B). Although the full-length transcript was severely decreased in homozygotes, the small amount still produced would generate mitochondrial *Wars2* tryptophanyl-tRNA synthetase 2 protein (mtTrpRS) (with the p.V117L substitution) and explains why *Wars2*^*V117L/V117L*^ mutants were viable, unlike *Wars2*^−/−^ nulls (Table S1).

We then investigated changes in RNA splicing using three TaqMan probes targeting *Wars2* 2/3, 4/5, and 5/6 exon-exon boundaries in tissues (Figure 4C). Exon 4/5 and exon 5/6 probes, present in all transcripts, were significantly reduced in the heart, kidney, and BAT and were unchanged in the other tissues (Figure 4C). Exon 2/3 junctions, present only in full-length transcript (*Wars2*^*FL*^), were significantly reduced in all *Wars2*^*V117L/V117L*^ tissues (Figure 4C). To determine the effect of these differences on WARS2 steady-state protein levels, tissues from *Wars2*^*V117L/V117L*^ mice were analyzed by immunoblotting. Consistent with the RNA results, WARS2 protein was significantly decreased in heart, liver, kidney, skeletal muscle, iWAT, and BAT of *Wars2*^*V117L/V117L*^ mice (Figure 4D; Figure S5A).

### The *Wars2* c.349G>T Mutation Caused Tissue-Specific OXPHOS Deficiencies

To determine the functional effects of reduced WARS2 protein in the mitochondria, steady-state levels of mitochondrial oxidative phosphorylation (OXPHOS) components (complex I–CV) were quantified by immunoblotting in each tissue at 12 months of age (Figures 4E–4J; Figure S5A). Consistent with decreased WARS2 protein, steady-state CI (NADH:ubiquinone oxidoreductase subunit B8 [NDUFB8]) and CIV (mitochondrially encoded cytochrome *c* oxidase I [MTCO1]) protein levels were significantly lower in *Wars2*^*V117L/V117L*^ heart (Figure 4E; Figure S5A), liver (Figure 4F; Figure S5A), and BAT (Figure 4G; Figure S5A). Furthermore, respiratory complex activity measurements in heart showed decreased CI and CIV activities (Figures S5B and S5C). In addition, CIII (ubiquinol:cytochrome *c* reductase core protein 2 [UQCRC2]) steady-state protein levels were decreased in *Wars2*^*V117L/V117L*^ BAT, showing more profound inhibition of mitochondrial translation in BAT compared to heart or liver (Figure 4G; Figure S5A). By comparison, no differences in CII (succinate:ubiquinone oxidoreductase complex flavoprotein subunit A [SDHA]), CIII, and CV (ATP synthase F1 subunit alpha [ATP5A]) subunit protein levels were observed in *Wars2*^*V117L/V117L*^ heart or liver; CV subunit levels were mildly increased in *Wars2*^*V117L/V117L*^ BAT relative to wild-type controls (Figure S5A).

Despite a severe loss of WARS2 protein, OXPHOS subunit steady-state protein levels remained largely unchanged in the *Wars2*^*V117L/V117L*^ kidney, with only a significant reduction in CV and a trend to mildly reduce CI observed at 12 months of age (Figure 4H; Figure S5A). Furthermore, immunoblot analysis showed a significant increase in CI and a trend toward increased CIII (unadjusted p = 0.029) steady-state OXPHOS protein levels in *Wars2*^*V117L/V117L*^ iWAT, despite decreased WARS2 protein (Figure 4I; Figure S5A). *Wars2*^*V117L/V117L*^ skeletal muscle showed no consistent respiratory chain deficiencies (Figure 4J; Figure S5A), although CIII steady-state protein levels appeared to be increased in skeletal muscle. This was confirmed in skeletal muscle by measurement of respiratory chain complex activities, which also showed a significant increase in CIII activity (Figure S5C).

Given the observation of brain pathology in patients, we also determined steady-state WARS2 and OXPHOS components in brains of mice 3–5 months of age and observed clear reduction of WARS2 protein and complex I subunit deficiency and a trend toward CIV deficiency (Figures S5D–S5F), indicating that the brain is not spared.

### *Wars2*^*V117L/V117L*^ Mice Show Browning of WAT and Dysfunctional BAT Pathology

**Figure 5:**
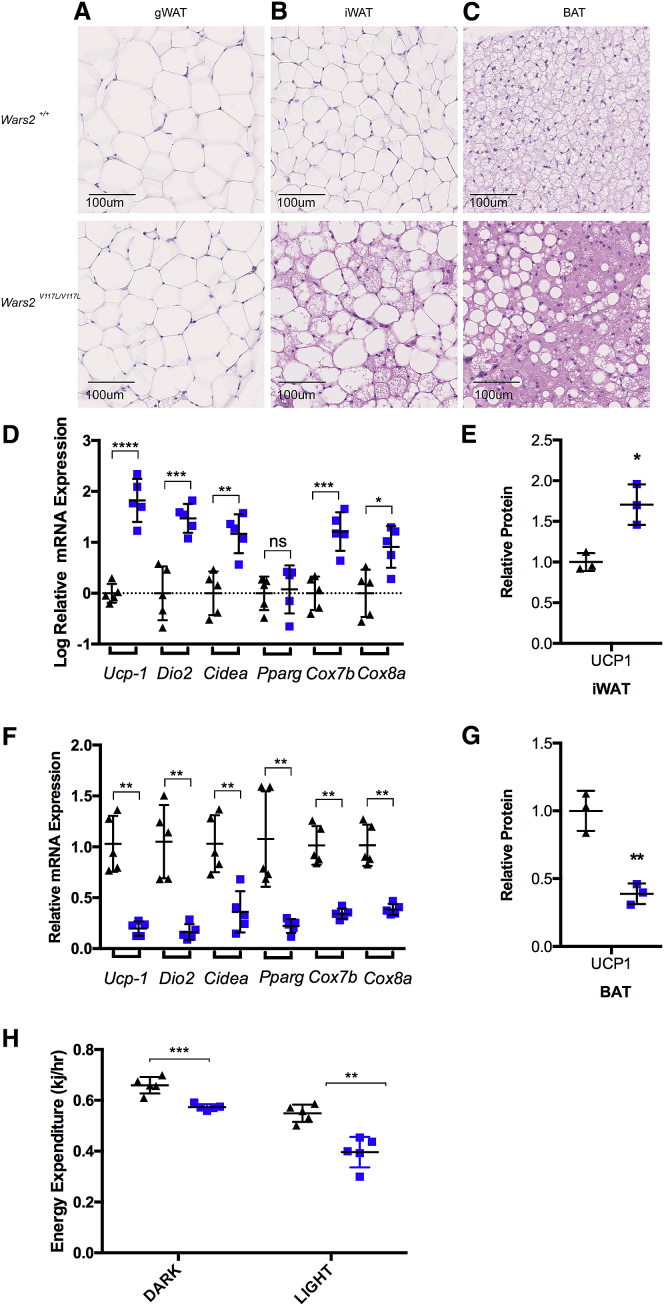
*Wars2*^*V117L/V117L*^ Mice Show Browning of WAT and Dysfunctional BAT (A–C) Representative images of H&E-stained sections of adipose tissue depots at 12 months of age from male mice. (A) Gonadal WAT (gWAT), (B) iWAT, and (C) BAT. Scale bar, 100 μm. *Wars2*^*V117L/V117L*^ and *Wars2*^+/+^ animal numbers were 3 and 3, respectively. (D) Relative mRNA expression analysis of browning markers in iWAT from male mice at 12 months of age. *Wars2*^*V117L/V117L*^ and *Wars2*^+/+^ animal numbers were 5 and 5, respectively; mean ± SD. Data were log transformed and analyzed using an unpaired two-tailed t test with equal SD. ^∗^p < 0.05, ^∗∗^p < 0.01, ^∗∗∗^p < 0.001. (E) Relative UCP1 protein levels in iWAT from female mice at 12 months of age. *Wars2*^*V117L/V117L*^ and *Wars2*^+/+^ animal numbers were 3 and 3, respectively; mean ± SD. Data were analyzed with an unpaired t test. ^∗^p < 0.05. (F) Relative mRNA expression analysis of browning markers in BAT from male mice at 12 months of age. *Wars2*^*V117L/V117L*^ and *Wars2*^+/+^ animal numbers were 8 and 8, respectively; mean ± SD. Data were analyzed with a Mann-Whitney two-tailed t test. ^∗∗^p < 0.01. (G) Relative UCP1 protein levels in BAT from female mice at 12 months of age. *Wars2*^*V117L/V117L*^ and *Wars2*^+/+^ animal numbers were 3 and 3, respectively; mean ± SD. Data were analyzed with an unpaired t test. ^∗∗^p < 0.01. (H) Energy expenditure (EE) normalized to lean mass by multiple linear regression (analysis of covariance [ANCOVA]) measured in female mice at 4 months of age. *Wars2*^*V117L/V117L*^ and *Wars2*^+/+^ animal numbers were 5 and 5, respectively; mean ± SD analyzed with an unpaired two-tailed t test. ^∗∗^p < 0.01, ^∗∗∗^p < 0.001. *Wars2*^*V117L/V117L*^ mice are blue squares, and *Wars2*^+/+^ mice are black triangles.

To investigate the functional consequences of the contrasting differences in iWAT and BAT for CI, CIII, and CIV subunit steady-state levels, we carried out histological analysis at 12 months of age in males. iWAT showed qualitatively higher multi-locular lipid droplet formation indicative of browning, although this was also observed to a lesser extent in wild-type mice. Visceral gWAT appeared relatively normal (Figures 5A and 5B). Similar patterns were seen in females at 3 months of age (data not shown). Gene expression analysis showed significant upregulation of the key browning markers uncoupling protein 1 (*Ucp1*), iodothyronine deiodinase 2 (*Dio2*), and cell death-inducing DNA fragmentation factor subunit alpha (DFFA)-like effector a (*Cidea*) (Figure 5D) and immunoblot analysis showed increased UCP1 protein levels (Figure 5E) in *Wars2*^*V117L/V117L*^ iWAT, showing activation of browning pathways. Furthermore, nuclear-encoded mitochondrial respiratory chain cytochrome *c* oxidase subunit 7B (*Cox7b*) and cytochrome *c* oxidase subunit 8A (*Cox8a*) mRNA were significantly increased in *Wars2*^*V117L/V117L*^ iWAT (Figure 5D), showing transcriptional upregulation, consistent with the increased respiratory chain subunit protein levels shown earlier (Figure 4I).

Conversely, in BAT, males at 12 months (Figure 5C) and females at 3 months (data not shown) showed strikingly increased unilocular lipid droplet formation, indicative of inhibition of lipolysis and β-oxidation, and reduced BAT thermogenic function. In keeping with these observations, gene expression analysis showed downregulation of browning markers *Ucp1*, *Dio2*, and *Cidea*; and peroxisome proliferator-activated receptor gamma (*Pparγ*) (Figure 5F) and immunoblot analysis showed reduced UCP1 protein (Figure 5G) in males at 12 months, consistent with BAT dysfunction. Nuclear-encoded mitochondrial respiratory chain subunit *Cox7b* and *Cox8a* mRNA expression levels were also decreased (Figure 5F), showing transcriptional downregulation, consistent with the respiratory chain dysfunction shown earlier (Figure 4G).

Given the abnormal BAT pathology and tissue-specific respiratory chain dysfunction observed, we carried out indirect calorimetry using a comprehensive laboratory animal monitoring system (CLAMS) at 4 months of age at 22°C (home cage temperature well below ∼28°C thermoneutrality). Energy expenditure (EE) was significantly reduced in female *Wars2*^*V117L/V117L*^ mice (Figure 5H), consistent with the observed abnormal BAT pathology and tissue-specific respiratory chain dysfunction.

### Upregulation of Mitochondrial Biogenesis Ameliorated Mitochondrial Respiratory Chain Dysfunction in *Wars2*^*V117L/V117L*^ MEFs, Skeletal Muscle, and iWAT

**Figure 6:**
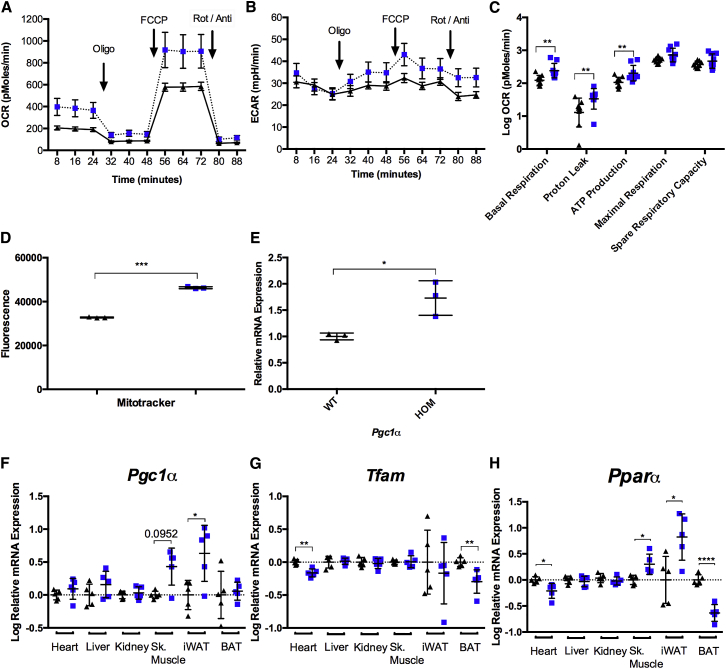
Upregulation of Mitochondrial Biogenesis Prevents Mitochondrial Respiratory Chain Dysfunction in *Wars2*^V117L/V117L^ MEFs, Skeletal Muscle, and iWAT (A and B) Oxygen consumption rate (OCR) (A) and extracellular acidification rate (ECAR) (B) were measured in cultured primary mouse embryonic fibroblasts harvested from *Wars2*^*V117L/V117L*^ and *Wars2*^+/+^ embryos using a Seahorse XF24 analyzer. OCR and ECAR measurements were taken at baseline and following oligomycin (Oligo), carbonyl cyanide 4-(trifluoromethoxy)phenylhydrazone (FCCP), and rotenone and antimycin (Rot/Anti) treatment. OCR and ECAR measurements were normalized to live cell number. (C) Relative oxygen consumption rates of basal respiration, proton leak, ATP production, maximal respiration, and spare respiratory capacity in *Wars2*^+/+^ and *Wars2*^*V117L/V117L*^ MEF cultures. There were 9 replicates of each genotype; mean ± SD. Data were log transformed and analyzed using an unpaired two-tailed t test or a Mann-Whitney t test. (D) MEFs were stained with MitoTracker green, and fluorescence in 30,000 cells per sample was quantified by fluorescence-activated cell sorting (FACS). There were 3 replicates of each genotype; mean fluorescence ± SD. Data were analyzed using an unpaired t test. (E) Relative mRNA expression analysis of *Pgc1α* in *Wars2*^+/+^ and *Wars2*^*V117L/V117L*^ MEF cultures. There were 3 replicates of each genotype; mean fluorescence ± SD. Data were analyzed using an unpaired t test. (F–H) Relative mRNA expression analysis of (F) *Pgc1α*, (G) *Tfam*, and (H) *Pparα* in tissues harvested from *Wars2*^*V117L/V117L*^ and *Wars2*^+/+^ male mice at 12 months of age. *Wars2*^*V117L/V117L*^ and *Wars2*^+/+^ animal numbers were 5 and 5, respectively; mean ± SD. Data are shown as log transformed and analyzed using an unpaired two-tailed t test or a Mann-Whitney t test (skeletal muscle and iWAT for *Pgc1α*). ^∗^p < 0.05, ^∗∗^p < 0.01, ^∗∗∗^p < 0.001, ^∗∗∗∗^p < 0.0001. *Wars2*^*V117L/V117L*^ mice are blue squares, and *Wars2*^+/+^ mice are black triangles.

We further examined the effects of *Wars2*-V117L on mitochondrial function in *Wars2*^*V117L/V117L*^ mouse embryonic fibroblasts (MEFs), which were cultured and assayed using microscale oxygraphy (Figures 6A and 6B). Unexpectedly, *Wars2*^*V117L/V117L*^ MEFs showed significantly increased basal respiration and ATP production compared to wild-type MEFs (Figures 6A and 6C), indicative of increased mitochondrial respiratory chain function. There was no difference in glycolysis as measured by extracellular acidification rate (ECAR) (Figure 6B). We hypothesized that this could be due to increased mitochondrial mass and upregulation of mitochondrial biogenesis. To directly measure mitochondrial mass, MEFs were stained with MitoTracker green, which localizes to mitochondria in live cells independent of mitochondrial membrane potential, and the average fluorescence per cell (30,000 cells per sample) was quantified by fluorescence-activated cell sorting (Figure 6D). We found that the average fluorescence intensity increased 40% in *Wars2*^*V117L/V117L*^ MEFs relative to *Wars2*^+/+^ controls showing increased mitochondrial mass (Figure 6D). Consistent with this, the master regulator of mitochondrial biogenesis, peroxisome proliferator-activated receptor gamma coactivator 1-alpha (*Pgc1α*), was significantly upregulated in *Wars2*^*V117L/V117L*^ MEFs (Figure 6E).

MEFs are derived from the mesenchyme stem cell lineage. We hypothesized that mature tissues composed primarily of cells derived from the mesenchymal stem cell lineage, such as myocytes and adipocytes (skeletal muscle and iWAT, respectively), upregulate *Pgc1α* and mitochondrial biogenesis to prevent respiratory chain dysfunction in *Wars2*^*V117L/V117L*^ mice. Gene expression analysis showed *Pgc1α* increased, on average, 3.3- and 4.3-fold in *Wars2*^*V117L/V117L*^ skeletal muscle and iWAT, respectively, at 12 months of age (Figure 6F), showing transcriptional upregulation of mitochondrial biogenesis, consistent with the increased respiratory chain subunits observed previously (Figures 4I and 4J). No significant differences in *Pgc1α* expression were observed in other tissues (Figure 6F).

These data show *Pgc1α* is upregulated in *Wars2*^*V117L/V117L*^ tissues displaying increased respiratory chain subunit levels, such as iWAT, indicating upregulation of mitochondrial biogenesis prevented respiratory chain dysfunction. Furthermore, *Pgc1α* is not upregulated in *Wars2*^*V117L/V117L*^ heart or BAT, in which respiratory chain dysfunction and disease pathology were observed, or in kidney, in which respiratory chain subunit levels are comparable with controls. Transcription factor A, mitochondrial (*Tfam*), required for transcription and associated with mtDNA copy number, was reduced in heart and BAT (Figure 6G). In addition, peroxisome proliferator-activated receptor alpha (*Pparα*) expression was increased in skeletal muscle and iWAT of *Wars2*^*V117L/V117L*^ mice, consistent with increased *Pgc1α* expression (Figure 6H). Conversely, *Pparα* was significantly decreased in the heart and BAT of *Wars2*^*V117L/V117L*^ mice and was unchanged in liver and kidney (Figure 6H). Overall, these data show that the tissue-specific respiratory chain dysfunction observed is partly because of the tissue-specific capacity for upregulation of *Pgc1α* and compensatory mitochondrial biogenesis.

### Heart-Specific Activation of the ISR Caused Increased Plasma FGF21 and Systemic Changes in Metabolism

**Figure 7:**
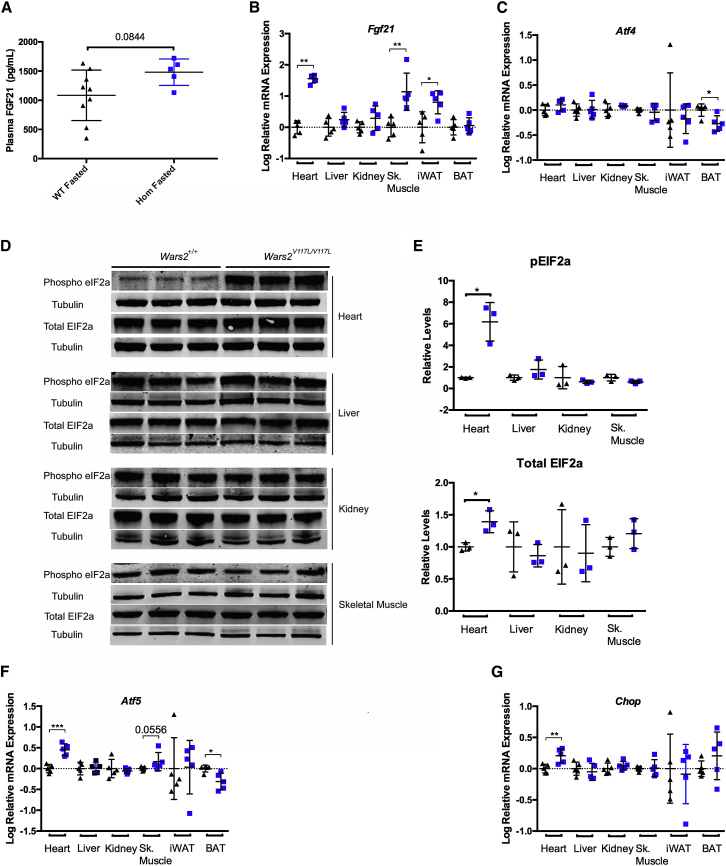
Heart-Specific Activation of the Integrated Stress Response Causes Increased Plasma FGF21 and Systemic Changes in Metabolism (A) Relative plasma FGF21 protein levels in male mice at 12 months of age. *Wars2*^*V117L/V117L*^ and *Wars2*^+/+^ animal numbers were 5 and 9, respectively; mean ± SD. Unpaired t test. (B and C) Relative mRNA expression levels of (B) *Fgf21* and (C) *Atf4* in tissues from male mice at 12 months of age. *Wars2*^*V117L/V117L*^ and *Wars2*^+/+^ animal numbers were 5 and 5, respectively; mean ± SD. Data were log transformed and analyzed using t tests or a Mann-Whitney test (*Fgf21* in heart and *Atf4* in skeletal muscle and iWAT). *Fgf21* RNA expression is very low in kidney and in wild-type skeletal muscle: mean C_T_ > 33. (D and E) Immunoblot analysis (D) and quantification (E) of p-eIF2α and total EIF2α protein levels in heart, liver, kidney, and skeletal muscle from female mice at 12 months of age. *Wars2*^*V117L/V117L*^ and *Wars2*^+/+^ animal numbers were 3 and 3, respectively; bands were normalized to tubulin and expressed relative to wild-type as the mean ± SD. Significance was determined using an unpaired t test with Welch’s correction. (F and G) Relative mRNA expression levels of (F) *Atf5* and (G) *Chop* in male *Wars2*^*V117L/V117L*^ and *Wars2*^+/+^ tissues harvested at 12 months of age. *Wars2*^*V117L/V117L*^ and *Wars2*^+/+^ animal numbers were 8 and 8, respectively; mean ± SD. Data were log transformed and analyzed using a t test or a Mann-Whitney test (*Atf5* and *Chop* in kidney and skeletal muscle). ^∗^p < 0.05, ^∗∗^p < 0.01, ^∗∗∗^p < 0.001. *Wars2*^*V117L/V117L*^ mice are blue squares, and *Wars2*^+/+^ mice are black triangles. See also Figures S6 and S7.

Fasted plasma FGF21 protein levels showed a trend toward elevation in male *Wars2*^*V117L/V117L*^ mice relative to controls at 12 months (Figure 7A) and similarly, but reaching significance, at 4 months of age in males only (Figures S6A and S6B). Plasma clinical chemistry analysis showed unchanged plasma free fatty acid levels and plasma glucose levels (Figures S6C and S6D), trends for reduced plasma triglycerides (Figure S6E), and markedly increased plasma ketone bodies (β-hydroxybutyrate) (Figure S6F) in *Wars2*^*V117L/V117L*^ mice. Furthermore, intraperitoneal glucose tolerance tests (IPGTTs) demonstrated increased glucose tolerance relative to wild-type controls (Figure S6G). FGF21 has previously been shown to reduce body weight by stimulating WAT lipolysis, induce temperature-dependent browning of WAT (Fisher et al., 2012), increase glucose tolerance by increasing insulin-independent glucose uptake in WAT and skeletal muscle (Kharitonenkov et al., 2005, Mashili et al., 2011), and increase hepatic ketogenesis (Inagaki et al., 2007). Thus, our findings, together with the reduced adiposity phenotype (Figure 2) and increased WAT browning observed previously (Figures 5A and 5B), align with the known effects of FGF21 on systemic metabolism and implicate FGF21 as the cause of metabolic phenotypes observed in *Wars2*^*V117L/V117L*^ mice.

FGF21 has previously been shown to be transcriptionally regulated via independent pathways governed by ATF4 and PPARα (Inagaki et al., 2007, Kim et al., 2013). To determine the mechanism of increased plasma FGF21, gene expression analysis was performed in *Wars2*^*V117L/V117L*^ mice at 12 months of age. *Fgf21* expression was significantly increased in heart, skeletal muscle, and iWAT (Figure 7B). No difference was observed in other tissues (Figure 7B). A significant reduction in *Atf4* expression was observed in BAT, but not in other tissues (Figure 7C). However, regulation of ATF4 at the protein level is key in *Fgf21* regulation. In other tissues, such as skeletal muscle and iWAT, an alternate mechanism governed by *Pparα* could contribute to the increased plasma FGF21 observed in *Wars2*^*V117L/V117L*^ mice (Figure 6H).

Upon various cellular stresses, the ISR is activated by phosphorylation of eukaryotic translation initiation factor 2A (eIF2α), resulting in reduced cytoplasmic 5′ cap-dependent protein synthesis and preferential translation of mRNAs that contain upstream open reading frames in their 5′ UTR, such as ATF4 (Lu et al., 2004). ATF4 has been shown to transcriptionally regulate stress response genes, including *Atf5*, DNA damage-inducible transcript 3 (C/EBP homologous protein [*Chop*]), and *Fgf21* (De Sousa-Coelho et al., 2012). We hypothesized that the *Wars2*-V117L allele caused cardiac-specific activation of the ISR pathway, leading to increased *Fgf21* expression and activation of stress response pathways. To assess activation of the ISR, we performed immunoblot analysis of phosphorylated eukaryotic translation initiation factor 2A (p-eIF2α)/eIF2α steady-state protein levels in *Wars2*^*V117L/V117L*^ heart, liver, kidney, and skeletal muscle at 12 months of age (Figures 7D and 7E). We found that p-eIF2α levels are significantly increased in heart (average of 6.1-fold) compared to controls (Figure 7E). Conversely, no significant differences in p-eIF2α levels were observed in kidney, liver, or skeletal muscle (Figure 7E). Furthermore, ISR pathway genes such as *Atf5* and *Chop* were significantly increased at the mRNA level in the heart of *Wars2*^*V117L/V117L*^ mice (Figures 7F and 7G). However, no significant differences in *Atf5* or *Chop* were observed in liver, kidney, skeletal muscle, or iWAT (Figures 7F and 7G). Altogether, these data show robust tissue-specific activation of the ISR in the heart of *Wars2*^*V117L/V117L*^ mice.

### Progressive Activation of the ISR Is Coincident with CI Deficiency and Independent of Disrupted Mitochondrial Proteostasis in the Heart of *Wars2*^*V117L/V117L*^ Mice

Disrupted mitochondrial proteostasis, rather than respiratory chain dysfunction, was the primary stress caused by inhibition of mitochondrial translation, leading to ATF4-dependent FGF21 expression and systemic changes in metabolism in the heart of *Dars2*-KO^Ckmm^ mice (Dogan et al., 2014). To characterize activation of the ISR in the heart, we performed time course immunoblot analysis of the ISR marker p-eIF2α, CI and CIV OXPHOS subunits, and mitochondrial proteostasis markers caseinolytic mitochondrial matrix peptidase proteolytic subunit (CLPP), Lon peptidase 1, mitochondrial (LONP1), heat shock protein family D (Hsp60) member 1 (HSP60), and mitochondrial heat shock protein family A (Hsp70) member 9 (mtHSP70) at 1, 3, and 12 months of age (Figures S7A–S7C). At 1 month, no differences in p-eIF2α, CI (NDUFB8), or CIV (COXI) steady-state protein levels were observed (Figure S7A). At 3 and 12 months of age, p-eIF2α levels were increased (Figure 7E; Figures S7B and S7C). At 3 and 12 months of age, CI and CIV levels were decreased (Figures S7B and S7C). Altogether, these data show that activation of the ISR and respiratory chain dysfunction occurred after 1 month of age in the heart of *Wars2*^*V117L/V117L*^ mice and that activation of the ISR was progressive, with age from 3 to 12 months, and coincident with progressive CI deficiency. CIV deficiencies were comparable between 3 and 12 months of age, showing no progressive further deficiency.

Finally, we found no significant differences in the steady-state protein levels of LONP1, CLPP, HSP60, or mtHSP70 at any time point measured in the heart of *Wars2*^*V117L/V117L*^ mice with the exception of a mild reduction in LONP1 protein at only 1 month of age (Figures S7A–S7C).

## Discussion

Sensorineural hearing loss is a common feature of human mitochondrial disease, and mutations in *LARS2*, *HARS2*, and *NARS2* have been shown to cause sensorineural hearing loss (Pierce et al., 2011, Pierce et al., 2013, Simon et al., 2015). Reduced body mass is also associated with mitochondrial diseases (Wolny et al., 2009). Furthermore, mutations in genes encoding critical proteins of the mitochondrial translation system, including *MTO1*, *GTBP3*, and *ELAC2* (Baruffini et al., 2013, Haack et al., 2013, Kopajtich et al., 2014) and several mt-aaRS mutations in *AARS2*, *PARS2*, *SARS2*, and *YARS2*, have been shown to cause hypertrophic cardiomyopathy (Belostotsky et al., 2011, Götz et al., 2011, Riley et al., 2010, Rivera et al., 2013, Shahni et al., 2013, Sofou et al., 2015). Individuals with compound heterozygous *WARS2* mutations showing neurological problems have been reported (Burke et al., 2018, Musante et al., 2017, Theisen et al., 2017, Vantroys et al., 2018, Wortmann et al., 2017). We demonstrate that hypomorphic *Wars2* alleles, *Wars2*^V117L/−^ and *Wars2*^V117L/V117L^, which do not have direct genocopies in humans, cause sensorineural hearing loss, reduced adiposity, and hypertrophic cardiomyopathy in mice. However, we have not observed gross neurological effects during welfare observations or morphological differences using light microscopy of brain sections. This may reflect the severity of the alleles described so far in patients, in comparison with this mouse hypomorphic allele, and possible species differences.

Genetic mapping in the rat for coronary flow and capillary density traits in the heart identified a causal missense variant in *Wars2* that reduced WARS2 activity by ∼40% (Wang et al., 2016). It was also shown that the mutation reduced endothelial cell proliferation and activated pro-apoptotic pathways, as well as impairing BAT function (Pravenec et al., 2017, Wang et al., 2016). Finally, Wang et al. (2016) demonstrated that Wars2 is a critical pro-angiogenic factor in zebrafish. We have not carried out an analysis of coronary vasculature in the *Wars2*^V117L/V117L^ model, although we observed BAT dysfunction.

As in our model, human hypomorphic mt-aaRS mutations cause tissue-specific pathology and respiratory chain dysfunction in humans, although the tissue-specific mechanisms remain unknown. However, increased respiratory chain subunits observed in iWAT of *Wars2*^*V117L/V117L*^ were associated with upregulation of *Pgc1α* mRNA expression. Furthermore, we showed increased *Pgc1α* expression, mitochondrial mass, and function in *Wars2*^*V117L/V117L*^ MEFs. We suggest that *Pgc1α* is upregulating mitochondria mass, preventing impaired respiratory chain function. In support of this, targeting *Pgc1α* to upregulate mitochondrial biogenesis, via therapeutic administration or genetic manipulation, can alleviate disease traits and increase mitochondrial respiratory capacity in human patient cell lines and mouse models (Bastin et al., 2008, Khan et al., 2014). Altogether, these data indicate that tissue-specific upregulation of mitochondrial biogenesis explains the pattern of respiratory chain deficiencies observed in *Wars2*^*V117L/V117L*^ mice. However, it remains unclear how some tissues in *Wars2*^*V117L/V117L*^ mice upregulate *Pgc1α* and are protected while other tissues are not. We speculate that this is explained partly by exogenous FGF21, because studies have shown that FGF21 regulates browning of WAT in response to adaptive thermogenesis and that this effect is partly because of increased PGC1a protein levels (Fisher et al., 2012). In addition, FGF21, which potentially signals metabolic demands from stressed mitochondria to other tissues in the body, was shown to regulate mitochondrial mass in BAT of polymerase gamma mutator (POLG) mice following high fat diet (HFD) administration (Wall et al., 2015). Some effects of FGF21, such as in fat, could also be through an autocrine or paracrine mechanism, as reported in thermogenic recruitment of WAT (Fisher et al., 2012). FGF21, a biomarker of mitochondrial translation defects in human, likely has a beneficial role in tissues such as WAT by upregulating browning and mitochondrial biogenesis, providing some explanation for the tissue-specific respiratory chain deficiencies observed in *Wars2*^*V117L/V117L*^ mice.

Common single-nucleotide polymorphisms, such as rs984222, with an effect allele frequency of 0.635, are associated with a 45% reduction in *WARS2* RNA expression in multiple tissues, including adipose (GTExPortal, http://www.gtexportal.org/home/). These single-nucleotide polymorphisms (SNPs) are associated with the waist-hip ratio in human genome-wide association studies, which are explained by changes in adipose tissue distribution (Heid et al., 2010). Our studies support the possibility that *WARS2* is one of the effector genes in this association locus (Pravenec et al., 2017).

Oxidative stress, mitochondrial unfolded protein response (UPR^mt^), inhibition of mitochondrial translation, and respiratory chain dysfunction are linked to activation of the ISR (Baker et al., 2012, Kim et al., 2013, Michel et al., 2015, Rath et al., 2012). Dogan et al. (2014) showed that knocking out *Dars2* caused tissue-specific activation of the UPR^mt^, leading to ATF4-dependent *Fgf21* expression in the heart of *Dars2*^*Ckmm*^ mice before respiratory chain dysfunction and concluding that mitochondrial proteostasis was the primary stress. We also demonstrated that activation of the ISR was a cardiac-specific response to inhibition of mitochondrial translation in *Wars2*^*V117L/V117L*^ mice, resulting in increased *Fgf21* gene expression. However, in contrast with the Dogan et al. (2014) findings, we showed that activation of the ISR was independent of UPR^mt^ activation, was progressive with age, and was coincident with progressive CI respiratory chain deficiency. Several studies have demonstrated activation of the ISR upon progressive respiratory chain deficiency independent of activation of the UPR^mt^. Inhibition of expression of the mitochondrial genome via mtDNA depletion or inhibition of mitochondrial translation through doxycycline treatment caused respiratory complex deficiencies and activation of the ISR independent of UPR^mt^ activation *in vitro* (Michel et al., 2015). Furthermore, activation of the ISR due to doxycycline treatment depended on the eIF2α kinase GCN2 (Michel et al., 2015). Activation of the ISR in the heart of *Wars2*^*V117L/V117L*^ mice is thus due to progressive respiratory chain dysfunction, is independent of the UPR^mt^, and may occur via GCN2-dependent phosphorylation of eIF2α.

We conclude that inhibition of mitochondrial translation can cause ISR activation via alternate mechanisms that depend upon the degree of mitochondrial translation inhibition. We speculate that complete inhibition of mitochondrial translation, e.g., via *Dars2*-KO, results in the accumulation of unassembled nuclear-encoded respiratory chain subunits, causing severe proteostatic stress and UPR^mt^-dependent ISR activation. In contrast, partial inhibition of mitochondrial translation, e.g., *Wars2*^*V117L/V117L*^ heart, causes activation of the ISR due to respiratory chain dysfunction and loss of mitochondrial membrane potential. The failed ability of the ISR to attenuate mitochondrial proteostatic stress likely explains the increased severity of the cardiac phenotype observed in *Dars2*-KO^Ckmm^ mice that cannot survive beyond 6 weeks of age compared to *Wars2*^*V117L/V117L*^ mice.

In summary, we have generated a key mouse model for studying tissue-specific deficits in mitochondrial protein translation, linking phenotypes and mechanisms and offering the potential for therapeutic testing.

## STAR★Methods

### Key Resources Table

**Table:** 

REAGENT or RESOURCE	SOURCE	IDENTIFIER
**Antibodies**		

WARS2 – rabbit polyclonal by Covolab	This paper	N/A
Mouse monoclonal anti-NDUFB8	Abcam	Cat#ab110242; CLONE No: 20-E9DH10C12; RRID: AB_10859122
Mouse monoclonal anti-SDHA	Abcam	Cat#ab14715; CLONE No: 2E3GC12FBZAE2; RRID:AB_301433
Mouse monoclonal anti-UQCRC2	Abcam	Cat#ab14745; CLONE No: 13G12AF12BB11; RRID:AB_2213640
Mouse monoclonal anti-MTCO1	Abcam	Cat#ab14705; CLONE No: 1D6E1A8; RRID:AB_2084810
Mouse monoclonal anti-ATP5A	Abcam	Cat#ab14748; CLONE No: 15H4C4; RRID:AB_301447
Rabbit monoclonal anti-eIF2α	Cell signaling	Cat#5324; CLONE No: D7D3; RRID:AB_10692650
Rabbit monoclonal anti-phosphor-Ser51-eIF2α	Epitomics	Cat#1090-1/ab32157; CLONE No: E90; RRID:AB_732117
Rabbit polyclonal anti-ATF4	Santa Cruz	Cat#sc-22800; LOT No: CREB-2(H-290); RRID:AB_2058742
Rabbit polyclonal anti-LONP1	Abcam	Cat#ab103809; RRID:AB_10858161
Rabbit monoclonal anti-CLPP	Abcam	Cat#ab124822; CLONE No: EPR7133; RRID:AB_10975619
Rabbit polyclonal anti-HSP60	Abcam	Cat#ab46798; RRID:AB_881444
Mouse monoclonal anti-HSP70	Abcam	Cat#ab2799; CLONE No:JG1; RRID:AB_303311
Goat polyclonal anti-UCP1	Santa Cruz	Cat#sc-6529; LOT No: M17; RRID:AB_2213781
Mouse monoclonal anti-Actin	Millipore	Cat#MAB1501; CLONE No: C4; RRID:AB_2223041
Rabbit polyclonal anti-α-Tubulin	Cell Signaling	Cat#2144
Mouse monoclonal anti-GAPDH	Abcam	Cat#ab8245; CLONE No: 6C5; RRID:AB_2107448
Total mouse monoclonal anti-OXPHOS rodent WB antibody cocktail (5 monoclonal antibodies)	Abcam	Cat#ab110413; RRID:AB_2629281

**Chemicals, Peptides, and Recombinant Proteins**		

Seahorse XF Cell Mito Stress Test Kit	Agilent	Cat#103015-100

**Critical Commercial Assays**		

Plasma FGF21 Quantikine ELISA Mouse / Rat FGF-21 Immunoassays (ELISA)	R&D Systems	Cat#MF2100
Plasma Free fatty Acids	Alpha Labs	Reagent 1- 434-91795/ Reagent 2- 436-91995
Plasma Glucose	Beckman Coulter	OSR6121
Plasma triglycerides	Beckman Coulter	OSR61118
D-3-hydroxybutyrate	Randox	RB 1007
Blood Glucose (IPGTT) – Abbott Alphatrak strips	Larkmead Veterinary	N/A

**Deposited Data**		

Reference mouse genome sequence NCBI Mouse Build 38, mm10	Genome Reference Consortium	https://www.ncbi.nlm.nih.gov/assembly/GCF_000001635.20/
WARS2 (5EKD, Human mitochondrial tryptophanyl-tRNA synthetase bound by indolmycin and Mn^∗^ATP. Williams, T.L., Carter Jr., C.W.)	PDB	http://www.rcsb.org/

**Experimental Models: Cell Lines**		

Primary Mouse Embryo Fibroblasts (MEFS), wildtype and *Wars2*^*V117L/V117L*^	This paper	N/A

**Experimental Models: Organisms/Strains**		

Mouse *Wars2*:V117L	This paper and Potter et al., 2016	N/A
Mouse *Wars2:tm1(KOMP)Vlcg*	UCDAVIS KOMP Repository	KOMP: VG15335
C3H/Pde (Pde6b+ repaired mice)	MRC Harwell Institute	N/A
C57BL/6J	MRC Harwell Institute	JAX:000664, RRID:IMSR_JAX:000664

**Oligonucleotides**		

Primers for Genotyping, see Table S2	This Paper	N/A
Wars2 (Exon 2-3) (Mm04208965_m1)	ThermoFisher	CAT#: 4351372
Wars2 (Exon 4-5) (Mm04208967_m1)	ThermoFisher	CAT#: 4351372
Wars2 (Exon 5-6) (Mm00840490_m1)	ThermoFisher	CAT#: 4331182
Pgc1α (Mm01208835_m1)	ThermoFisher	CAT#: 4331182
Atf4 (Mm00515324_m1)	ThermoFisher	CAT#: 4331182
Atf5 (Mm00459515_m1)	ThermoFisher	CAT#: 4331182
Chop (Mm01135937_g1)	ThermoFisher	CAT#: 4331182
Fgf21 (Mm00840165_g1)	ThermoFisher	CAT#: 4331182
Tfam (Mm00447485_m1)	ThermoFisher	CAT#: 4331182
Pparα (Mm00440939_m1)	ThermoFisher	CAT#: 4331182
Ucp1 (Mm01244861_m1)	ThermoFisher	CAT#: 4331182
Dio2 (Mm00515664_m1)	ThermoFisher	CAT#: 4331182
Cidea (Mm00432554_m1)	ThermoFisher	CAT#: 4331182
Pparγ (Mm00440945_m1)	ThermoFisher	CAT#: 4331182
Cox7a1 (Mm00438297_g1)	ThermoFisher	CAT#: 4331182
Cox8b (Mm00432648_m1)	ThermoFisher	CAT#: 4331182

**Software and Algorithms**		

GenotypeCaller tool in the Genome Analysis Toolkit (GATK)	Potter et al., 2016	https://software.broadinstitute.org/gatk/
Phyre2	Kelley et al., 2015	http://www.sbg.bio.ic.ac.uk/∼phyre2/html/page.cgi?id=index
NetGene2	Hebsgaard et al., 1996	http://www.cbs.dtu.dk/services/NetGene2/
PyMOL by schrodinger	Schrodinger	https://pymol.org/2/
ImageJ 1.8.0_172	Schneider et al., 2012	https://imagej.nih.gov/ij/
SPSS	IBM	https://www.ibm.com/uk-en/products/spss-statistics
Graphpad PRISM v7.0d	GraphPad	https://www.graphpad.com/scientific-software/prism/

### Contact for Reagent and Resource Sharing

Further information and requests for resources and reagents should be directed to and will be fulfilled by the Lead Contact, Roger Cox (r.cox@har.mrc.ac.uk).

### Experimental Model and Subject Details

#### Animal Models

All mice used in this study were housed in the Mary Lyon Centre at MRC Harwell. Mice were kept and studied in accordance with UK Home Office legislation and local ethical guidelines issued by the Medical Research Council (Responsibility in the Use of Animals for Medical Research, July 1993; Home Office license 30/3146 and 30/3070). Procedures were approved by the MRC Harwell Animal Welfare and Ethical Review Board (AWERB). Mice were kept under controlled light (light 7am–7pm, dark 7pm–7am), temperature (21 ± 2°C) and humidity (55 ± 10%) conditions. They had free access to water (9–13 ppm chlorine) and were fed *ad libitum* on a commercial diet (SDS Rat and Mouse No. 3 Breeding diet, RM3, 3.6 kcal/g).

MPC-151 pedigree was generated from The Harwell Aging ENU-mutagenesis Screen as documented previously (Potter et al., 2016). These mice are C57BL/6J mutagenized mice crossed with C3H/Pde (Pde6b+ repaired mice) and subsequently maintained by backcrossing to C3H/Pde mice. Age and sex of mice is indicated in the figure legends. Estimates for required cohort sizes were made using GraphPad Statmate using trait data from previous experiments.

Cohorts of male and female mice were bred for longitudinal blood and body composition-based phenotyping tests. Four cohorts of mice *Wars2*^*V117L/V117L*^ mice were generated from *Wars2*^*+/V117L*^ x *Wars2*^*+/V117L*^ matings and were aged to 1- (20 mice total), 3- (58 mice total), 9- (74 mice total) and 12 months (58 mice total) of age before being humanely killed in accordance with Home Office schedule 1 regulations. *Wars2*^*V117L/-*^ mice were generated from *Wars2*^*+/−*^ x *Wars2*^*V117L/+*^ matings (78 mice total). *Ppa2*^*Y123F/Y123F*^ mice were generated from *Ppa2*^*+/Y123F*^ x *Ppa2*^*+/Y123F*^ matings (47 mice total). Mice were randomly assigned to cages at weaning before subsequent genotyping of individual mice. Downstream phenotyping experiments were performed blinded to the genotype of the mice.

For body composition three cohorts were analyzed, two with multiple time points and one at 1 month only. In the first cohort one wild-type mouse was humanely killed because it was sick and one found dead and all data from these animals was excluded. Final cohort sizes were *Wars2*^*+/+*^ n = 11 and 3, *Wars2*^*+/V117L*^ n = 13 and 17, *Wars2*^*V117L/V117L*^ n = 7 and 8, male and female respectively. In the second cohort data from one homozygous mouse was excluded after being found dead before the 6-month time-point and two heterozygous mice humanely killed to reduce cage numbers prior to starting phenotyping. Final cohort sizes were *Wars2*^*+/+*^ n = 13 and 18, *Wars2*^*+/V117L*^ n = 19 and 19, *Wars2*^*V117L/V117L*^ n = 11 and 7, male and female respectively. In cohort 3 there were *Wars2*^*+/+*^ n = 4, *Wars2*^*+/V117L*^ n = 15, *Wars2*^*V117L/V117L*^ n = 9 and none of the differences for body weight, fat mass or lean mass were significant (tested at one month only).

An additional fifth intercross cohort, congenic on C3H/Pde, was generated for additional replication experiments including OXPHOS blots and FGF21 measurements in plasma at 3-4 months.

The NIH KOMP *Wars2*-KO allele (*Wars2*^*tm1(KOMP)Vlcg*^) obtained from the KOMP repository (https://www.komp.org/) comprises a targeting construct integrated into the C57BL/6N ES cell genome by homologous recombination, deleting 46632bp of the *Wars2* gene locus, including coding regions of both *Wars2*-Exon1 and *Wars2*-Exon2, leading to a frameshift and a premature stop codon. *Wars2*^*tm1(KOMP)Vlcg*^ ES cells were micro-injected into C57BL/6N blastocysts generating mosaic C57BL/6N-*Wars2*^*tm1(KOMP)Vlcg*^ offspring. Germ-line transmission (GLT) of the *Wars2*^*tm1(KOMP)Vlcg*^ construct was determined by genotyping C57BL/6N-*Wars2*^*tm1(KOMP)Vlcg*^ x C57BL/6N offspring for the neomycin selection cassette.

#### Primary Cultures

MEFs were harvested from E12.5-14 (dpc) embryos from timed *Wars2*^*+/V117L*^ x *Wars2*^*+/V117L*^ matings and dissected on ice in Dulbeccos PBS (ThermoFischer 14190094). The sex of the embryos was unknown. Head, liver, heart and limbs were placed in 3ml GIBCO 0.25% trypsin (EDTA) (ThermoFischer 25200056) and minced using surgical scissors and then pipetted 10x using a P1000 pipette and sterile filter tip, before transfer to a 15ml Falcon tube and incubated at 37°C for 10 minutes. The trypsin was neutralized with 7ml of culture medium, DMEM (ThermoFischer 31966021) supplemented with 1 X NEAA (Sigma-Aldrich M7145), 1 X Penicillin/Streptomycin (ThermoFischer 15070-063), 50 μM 2-mercaptoethanol (2-mercaptoethanol (ThermoFischer 31350010) and 10% GIBCO FBS (ThermoFischer 10500064). Cells were then plated on a 10cm dish and incubated at 37°C and 5% CO_2_.

### Method Details

#### SNP Mapping and Whole Genome Sequencing

SNP mapping and NGS were performed as described previously (Potter et al., 2016). Briefly Individual mutations were mapped using the Illumina GoldenGate Mouse Medium Density Linkage Panel (Gen-Probe Life Sciences Ltd, UK) that utilizes over 900 SNPs for the C3H/Pde (Pde6b+ repaired mice) and C57BL/6J strains. The genotypes of G3 ‘affected’ (elevated ABR thresholds) MPC-151 mice were compared to ‘non-affected’ (‘normal’ ABR thresholds) littermate controls. This allowed us to identify a 75Mb region within which all ‘affected’ MPC-151 mice were homozygous for C57BL/6J SNPs and ‘non-affected’ MPC-151 littermates were either heterozygous or homozygous for C3H.Pde6b+ SNPs. To identify candidate causal ENU-induced mutations within the mapped region, WGS was performed using DNA from an ‘affected’ G3 MPC-151 mouse. WGS was performed as previously described (Potter et al., 2016). Briefly, following DNA extraction a library was generated and a single lane or paired-end sequencing (100nt) was performed using the Illumina HiSeq platform (Oxford Genomics Centre, Wellcome Centre for Human Genetics). The 100nt paired-end reads were aligned to the reference mouse genome (NCBIM38/mm10) using Burrows-Wheeler Aligner software (Li and Durbin, 2009). Single-nucleotide variants (SNVs) were identified for each alignment using the unified GenotypeCaller tool in the Genome Analysis Toolkit (GATK) as previously described (Potter et al., 2016). Here the mouse dbSNP version 137 was used as the background SNP set using default parameters. Identified SNVs were then given a quality score (Phred scaled quality score, −10 x log(1-p), p is the probability of a SNV being called incorrectly). SNVs with a quality score of < 100 or with a read depth of < 3 reads were removed from all further analysis. All remaining SNVs were termed ‘high-confidence’ mutations and were compared to previously identified SNPs from 17 inbred strains from the Mouse Genome Project (Keane et al., 2011) as well as an in-house library of SNVs. Any overlapping sites were removed leaving the final list of novel ENU-induced SNVs for the ‘affected’ MPC-151 G3 mouse. SNVs were annotated using NGS-SNP to give an indication of the nature of the SNV (e.g., Missense, splice-site variant or intronic). 3 high-confidence, ENU-induced, missense mutations were identified for the MPC-151 G3 ‘affected’ mouse that were located within the 75Mb mapped region as previously identified.

#### Genotyping

Mice were assayed for the presence or absence of ENU-induced mutations *Ppa2*^*A398T*^ and *Wars2*^*G349T*^ by pyrosequencing (Potter et al., 2016). PCR primers were designed to amplify the regions of interest using a biotinylated primer for the Pyrosequencing template strand. Ppa2^A398T^ primers: biotinylated forward (5′- CTCAATCCCATTAAGCAAGATAT-3′), reverse (5′-GGTTTCTGTAGAAGGCATAAAAG-3′) and sequencing reverse (5′-GGGAAGATGTTCGGTG-3′). Wars2^G349T^ primers: forward (5′-GGTCACCTTTCTTTCTCTCC-3′), biotinylated reverse (5′-CAGGTGAGGATCCAACTTAA-3′) and forward sequencing (5′-TTTCTCTCCTTCCTTTTAG-3′).

Mice generated from *Wars2*^*V117L/-*^ x *Wars2*^*+/−*^ matings were genotyped using two strategies. The *Wars2*^*V117L*^ allele was genotyped using the Idaho Technology LightScanner System (Idaho Technology Inc, Utah, USA) and was used in accordance with the manufacturers standard protocols. *Wars2*^*V117L*^ Primers: forward (5′-TCAGCCTATCCCTGTTGTCTA-3′), reverse (5′-TGGTGTAAATGCTGCAATCG-3′) and probe (5′-CCTTCCTTTTAGTTGTCTGAACACACTCAG-3′). The *Wars2*-KO allele was genotyped for the presence of the LacZ reporter cassette using a RT-PCR copy number assay using FAM-labeled taqman probes. Assays were performed using FAM-labeled TaqMan probes for LacZ and *Wars2*-WT DNA sequences as controls. Each assay was performed along with an additional VIC-labeled TaqMan probe designed to Dot1l that acted as an internal controls. Wars2^WT^ primers: forward (5′-GCCCAGCACTTGGGATGT-3′) and reverse (5′-GCAGCCAGCTCACCAATG-3′), FAM labeled probe (5′-TCCCTTCACTTTCCTGTCTCCGTTTC-3′). LacZ primers: forward (5′-CTCGCCACTTCAACATCAAC-3′), reverse (5′-TTATCAGCCGGAAAACCTACC-3′), FAM labeled probe (5′-TCGCCATTTGACCACTACCATCAATCC-3′). Dot1l primers: forward (5′-GCCCCAGCACGACCATT-3′), reverse (5′-TAGTTGGCATCCTTATGCTTCATC-3′) and VIC labeled probe (5′-CCAGCTCTCAAGTCG-3′).

#### Auditory phenotyping

Click box protocol as previously described (Hardisty-Hughes et al., 2010). Briefly, mice were placed on the operator’s palm and hearing was tested using a purpose built frequency calibrated click box (CB) that emits a 90 dB SPL tone at 20 kHz (CB apparatus was obtained from MRC Institute of Hearing Research, Nottingham, UK). The CB emits a tone that elicits a Preyer reflex from the mouse as seen by a visible flick of the pinna or a startle response if the mouse can hear. The presence or absence of a Preyer reflex is then scored as follows: 2 – normal startle response, 1 – reduced startle, 0 – no startle. CB testing was performed blinded and away from the home cage to prevent littermates from becoming attenuated to the CB tone. Auditory-evoked brainstem response (ABR) testing was performed as previously described (Hardisty-Hughes et al., 2010). Briefly, mice were anaesthetized via administration of an intra-peritoneal injection of anesthetic (1 mL Ketamine, 0.5 mL Xylasine, 8.5 mL sterile H_2_O) at a rate of 0.1 ml/10 g of body mass. Once unconscious, the mouse was placed on a heated mat in a sound proof booth. Electrodes were then placed sub-dermally below the right pinna (reference), into the muscle mass below the left ear (ground) and on the midline of the skull (active). Mice were placed with their auditory canal 1 cm from the speaker and were exposed to a broadband ‘click’ stimulus, followed by tones at 8, 16 and 32 kHz. The electrodes recorded the auditory brainstem responses to the tones. The recorded data was calibrated, generated and processed using the Tucker Davies Technology (TDT) system III. Following the ABR testing an IP injection of Antipamezole (0.1 mL in 9.9 mL of sterile water) at a rate of 0.1 mL < 50 g or 0.2 mL > 50 total body mass was administered to reverse the anesthetic.

#### Body weight and composition analysis

Body mass was measured monthly on scales calibrated to 0.01 g. Body composition was measured monthly using an Echo-MRI quantitative NMR machine (Echo-MRI-100, Echo-MRI, Texas, U.S.A.).

#### Echocardiograms

Mice were placed under general anesthetic using 4% isoflurane using an anesthetic chamber. Once unconscious the mouse was placed on an ECG platform (Visualsonics heatpad / ECG platform) and mouse limbs are taped to ECG probes to allow heart rate monitoring. Anesthesia was maintained using a nose cone and 1.5% (or as appropriate to maintain a heart rate < 400 bpm) isoflurane. Hair was removed from the mouse chest using hair clippers followed by hair removal cream. A rectal thermometer was inserted and used to monitor core body temperature throughout the procedure. Contact gel was applied to the shaven mouse chest and a 707B probe was lowered to the mouse chest locating the mouse heart left ventricle until contractions of the left ventricle could be monitored on the Visualsonics Vevo 770 high resolution *in vivo* micro imaging system. Several images of mouse heart were taken in M-mode and were analyzed using the Vevo 770 software. Following successful data capture, the rectal probe, contact gel and limb tape was removed and the mouse was placed in a heat box to recover from the anesthetic.

#### Comprehensive Laboratory Animal Monitoring System

The Comprehensive lab animal monitoring system (CLAMS) was used to measure mice energy expenditure at home cage temperature (22°C) according to standard protocols. Briefly mice were placed in individual cages for a total of 72 hours. Measurements of oxygen (O_2_) and carbon-dioxide (CO^2^) in-flow and out-flow concentrations were automatically monitored and recorded along with food consumption and water intake throughout the 72-hour period. Data from the first 24 hours was removed from the analysis as this period was used to allow the mice to acclimatize to their new environment. Data collected from the second 24-hour period was used for all subsequent analysis. Energy expenditure was calculated as follows EE = CV x VO_2_ (where CV = Calorific value = 3.815 + 1.232 x RER and VO_2_ = ViO_2_i - VoO_2_o (o = outflow, i = inflow). EE values were normalized to lean mass using multiple linear regression analysis (ANCOVA) as described previously (McMurray et al., 2013).

#### Intraperitoneal Glucose Tolerance Test (IPGTT)

Mice were fasted overnight and IPGTT were performed the following morning. On the morning of the IPGTT, mice were weighed and a local anesthetic was administered to the mouse tail (EMLA cream, Eutectic mixture of Local Anesthetics Lidocaine / Prilocaine, AstraZeneca, UK). A blood sample was collected from the mouse tail at time point zero in Lithium-Heparin microvette tubes (CB30, Sarstedr, Numbrecht, Germany) to establish a baseline blood glucose level. Mice were then administered an intra-peritoneal injection of 2 g glucose / kg body weight (20% glucose in 0.9% NaCl). Blood samples were then taken 60 and 120 mins post-injection. At each time point, blood glucose levels were measured using the handheld Alphatrak (Abbott) glucose monitor with a fresh Alphatrak strip (Abbott) being used for every reading.

#### Tissue collection

Mice were humanely killed at 1-, 3-, 9- and 12 months of age and tissues were harvested for analysis. Following confirmation of death: cochlea, heart, liver, kidney, iWAT, gonadal which adipose tissue, BAT and skeletal muscle were dissected. For subsequent protein, RNA and DNA analysis tissues were placed in cryotubes (Nunc, Thermo Fisher Scientific-Heraeus) and snap frozen in liquid nitrogen. Tissue samples were stored long-term at −70°C.

#### Blood Biochemistry and ELISA analysis

Food was withdrawn and mice were fasted at 8:00 AM. 4hrs later, mice were humanely killed by administration of an over-dose of anesthetic (0.2 mL of pentobarbitone) via intra-peritoneal injection in accordance with home office procedures. Once the mouse was fully anaesthetized a glass capillary is inserted into the anterior corner of the mouse eye to puncture the membrane of the retro-orbital sinus. Blood was collected from the capillary in Lithium-Heparin microvette tubes (CB30, Sarstedr, Numbrecht, Germany). Blood samples were centrifuged for 10 mins at 8000 x g at 8°C. The supernatant blood plasma was removed and analyzed on a Beckman Coulter AU680 clinical chemistry analyzer using reagents and settings recommended by the manufacturer. Plasma FGF21 proteins levels were assayed using Quantikine ELISA Mouse / Rat FGF-21 Immunoassays (R&D Systems) according to manufacturer’s instructions.

#### Mitochondrial stress test in MEFs

Oxygen consumption rate (OCR) and extracellular acidification rate (ECAR) were measured in MEFs using the Seahorse XF24 flux analyzer (Seahorse Bioscience). Primary MEFs were seeded at a density of 40000 cells/well on XF-24 tissue culture plate and left to adhere overnight. The following day MEFs media was replaced with XF Assay Media supplemented with L-glutamine 2 mM, sodium pyruvate 2 mM, and glucose 10 mM (pH 7.4) and were incubated for 1 hour at 37°C in a CO_2_ free incubator before being placed in the XF24 analyzer. OCR and ECAR measurements were measured under basal conditions and following administration of mitochondrial inhibitors oligomycin (1 μM), antimycin (1 μM) and rotenone (1 μM) or in the presence of the mitochondrial uncoupler FCCP (1 μM) (Seahorse XF Cell Mito Stress Test Kit, Agilent). Oxygen consumption rates were normalized to the number of live cells using the LIVE/DEAD Viability/Cytotoxicity kit (ThermoFisher) according to the manufactures instructions.

#### Respiratory chain complex activities

The activities of individual respiratory chain complex activities and citrate synthase, a mitochondrial matrix marker, were determined in skeletal muscle and cardiac muscle homogenates as previously described (Kirby et al., 2007).

#### Western blots analysis

Proteins were extracted from snap frozen mouse tissues using CelLyic MT Mammalian Tissue Lysis Buffer (Sigma- Aldrich) supplemented with 1 X complete protease inhibitor cocktail (1 μL / 100 μL lysis buffer, Sigma- Aldrich) and 1 X PhosStop phosphatase inhibitor cocktail (1 μL / 100 μL lysis buffer, Sigma Aldrich). Tissues were homogenized using the Precellys-24 automated homogenizer (Bertin Technologies). Tissue homogenates were centrifuged at 13,000 rpm for 15 mins at 4°C to pellet cell debris. The supernatant tissue lysates were isolated and protein concentrations were determined using the BCA (bicinchoninic acid) Protein Assay Reagent (BioRad). Samples were diluted to 4 μg / μL in lysis buffer and supplemented with NuPAGE LDS Sample Buffer (4X) and NuPage Reducing Agent (10X) and were denatured by heating to 70°C for 10mins. Protein samples were separated using 4%–12% linear gradient Bis-Tris ready polyacrylamide gels with 1 X MOPS electrophoresis running buffer (Invitrogen) using the XCell Surelock Mini Cell tanks (Invitrogen) at 200 V for 50 mins. Protein samples were electrotransferred from the gels onto PVDF membrane (Hybond – P, GE Healthcare Amersham) using a XCell II Blot Module (Invitrogen). Protein membranes were blocked in 5% non-fat milk Tris Buffered Saline with Tween 20 (TBST, Merk) (non-phosphor antibodies) or 5% Bovine Serum Albumin TBST (phosphor-antibodies) at room temperature for an hour or overnight at 4°C before being incubated with primary antibodies overnight at 4°C. Protein membranes were washed 3-5 times in TBST for 10mins at room-temperature. Secondary antibodies were diluted in 5% non-fat milk TBST. Membranes were incubated with species-specific secondary horseradish peroxidase (HRP) conjugated antibodies for 4 hr at room-temperature. Membranes were washed 5 times in TBST for 10 mins. Immunolabelled membrane were treated with Enhanced Chemiluminesence Plus (ECL plus; Amersham, GE Healthcare) and were imaged using the ChemiDoc UV chemiluminescent imager or exposure to X-ray film.

Primary antibodies used in this study: WARS2, at a 1:500 dilution (custom, Covalab); NDUFB8, at a 1:2,000 dilution (ab110242, Abcam); SDHA, at a 1:10,000 dilution (ab14715, Abcam); UQCRC2, at a 1:3,000 dilution (ab14745, Abcam); MTCO1, at a 1:2,000 dilution (ab14705, Abcam); ATP5A, at a 1:5,000 dilution (ab14748, Abcam); eIF2α, at a 1:1000 dilution (#5324, Cell signaling); phosphor-Ser51-eIF2α, at a 1:1,000 dilution (#1090-1, Epitomics); ATF4, at a 1:500 dilution (sc-22800, Santa Cruz); LONP1, at a 1:1,000 dilution (ab103809, Abcam); CLPP, at a 1:5,000 dilution (ab124822, Abcam); HSP60, at a 1:10,000 dilution (ab46798, Abcam); HSP70, at a 1:1,000 dilution (ab2799, Abcam); UCP1, at a 1:200 dilution (sc-6529, Santa Cruz); Actin, at a 1:5,000 dilution (MAB1501, Millipore); α-Tubulin, at a 1:5,000 dilution (#2144, Cell Signaling); and GAPDH, at a 1:10,000 dilution (ab8245, Abcam).

In subsequent OXPHOS blot experiments (Figure S5) a total OXPHOS rodent WB antibody cocktail was used at a 1:1,000 dilution (ab110413, Abcam).

#### Real-Time Quantitative PCR

Total RNA was extracted from MEFs and mouse tissues using the RNeasy Mini Plus Kit (QIAGEN) according to the manufacture’s protocol. RNA concentrations were determined using a NanoDrop spectrophotometer (Thermo Scientific). RNA samples were diluted to 200 ng/μl and reverse transcription reactions were performed using Super Script III reverse transcriptase (Invitrogen) following the manufacturer’s protocol to generate 2 μg of cDNA. mRNA gene expression analysis was performed using the TaqMan system. TaqMan Gene Expression Assay reagents and TaqMan FAM dye-labeled probes (Applied Biosystems, Invitrogen, U.S.A.) were used according to the manufacturers protocol and assays were performed using an ABIPRISM 7500 Fast Real-Time PCR System (Applied Biosystems). Data was normalized to house-keeping genes specific to the tissue / cell line being used. GeNORM analysis was performed for each cell / tissue used to determine the most suitable housekeeping gene. Data were analyzed using the comparative ΔΔCT method in order to determine the difference in sample groups relative to control samples. Taqman probes used in this study: *Wars2 (Exon 2-3) (*Mm04208965_m1), *Wars2 (Exon 4-5) (*Mm04208967_m1), *Wars2 (Exon 5-6) (*Mm00840490_m1), *Pgc1α (*Mm01208835_m1), *Atf4 (*Mm00515324_m1), *Atf5 (*Mm00459515_m1), *Chop (*Mm01135937_g1), *Fgf21 (*Mm00840165_g1), *Tfam (*Mm00447485_m1), *Pparα (*Mm00440939_m1), *Ucp1* (Mm01244861_m1), *Dio2 (*Mm00515664_m1), *Cidea (*Mm00432554_m1), *Pparγ (*Mm00440945_m1), *Cox7a1 (*Mm00438297_g1) and *Cox8b (*Mm00432648_m1).

#### Prediction of WARS2 3D structure

The crystal structure of human WARS2 (PDB: 5EKD, Human mitochondrial tryptophanyl-tRNA synthetase bound by indolmycin and Mn^∗^ATP. Williams, T.L., Carter Jr., C.W.) was downloaded from the PDB database (PDB; http://www.rcsb.org/). The predicted protein structure of human Wars2 was generated using PHYRE2 Protein fold recognition server (Kelley et al., 2015). The alignment and visualization of the protein structures was performed by PyMOL by Schrödinger (https://pymol.org/2/).

### Quantification and Statistical Analysis

Statistical tests in GraphPad Prism are indicated in the figure legends and were selected depending on whether data was normally distributed as assessed by the D’Agostino & Pearson omnibus normality test in Prism. Equal variance was assessed by an F-test in Prism and non-parametric tests used if this test was failed. Where necessary AUC’s were calculated using Prism to allow analysis of longitudinal data. Number of animals and cellular assay replicates are indicated in the figure legends.

Western blot bands were analyzed and quantified using ImageJ (Schneider et al., 2012).

Energy Expenditure adjustment for lean mass by ANCOVA using SPSS.

## Supplemental Information

Document S1. Figures S1–S7 and Tables S1 and S2

Document S2. Article plus Supplemental Information
