# Supplementary material for: A Wars2 Mutant Mouse Model Displays OXPHOS Deficiencies and Activation of Tissue-Specific Stress Response Pathways
Source: Cell Rep. 2018 Dec 18;25(12):3315–3328.e6. doi: 10.1016/j.celrep.2018.11.080 (PMC6315286; doi:10.1016/j.celrep.2018.11.080)
Supplement: Document S1. Figures S1–S7 and Tables S1 and S2 [file mmc1.pdf]

Cell Reports, Volume *25*

Supplemental Information

A Wars2 Mutant Mouse Model Displays OXPHOS Deficiencies and Activation of Tissue-Specific Stress Response Pathways

Thomas Agnew, Michelle Goldsworthy, Carlos Aguilar, Anna Morgan, Michelle Simon, Helen Hilton, Chris Esapa, Yixing Wu, Heather Cater, Liz Bentley, Cheryl Scudamore, Joanna Poulton, Karl J. Morten, Kyle Thompson, Langping He, Steve D.M. Brown, Robert W. Taylor, Michael R. Bowl, and Roger D. Cox

Supplementary Information

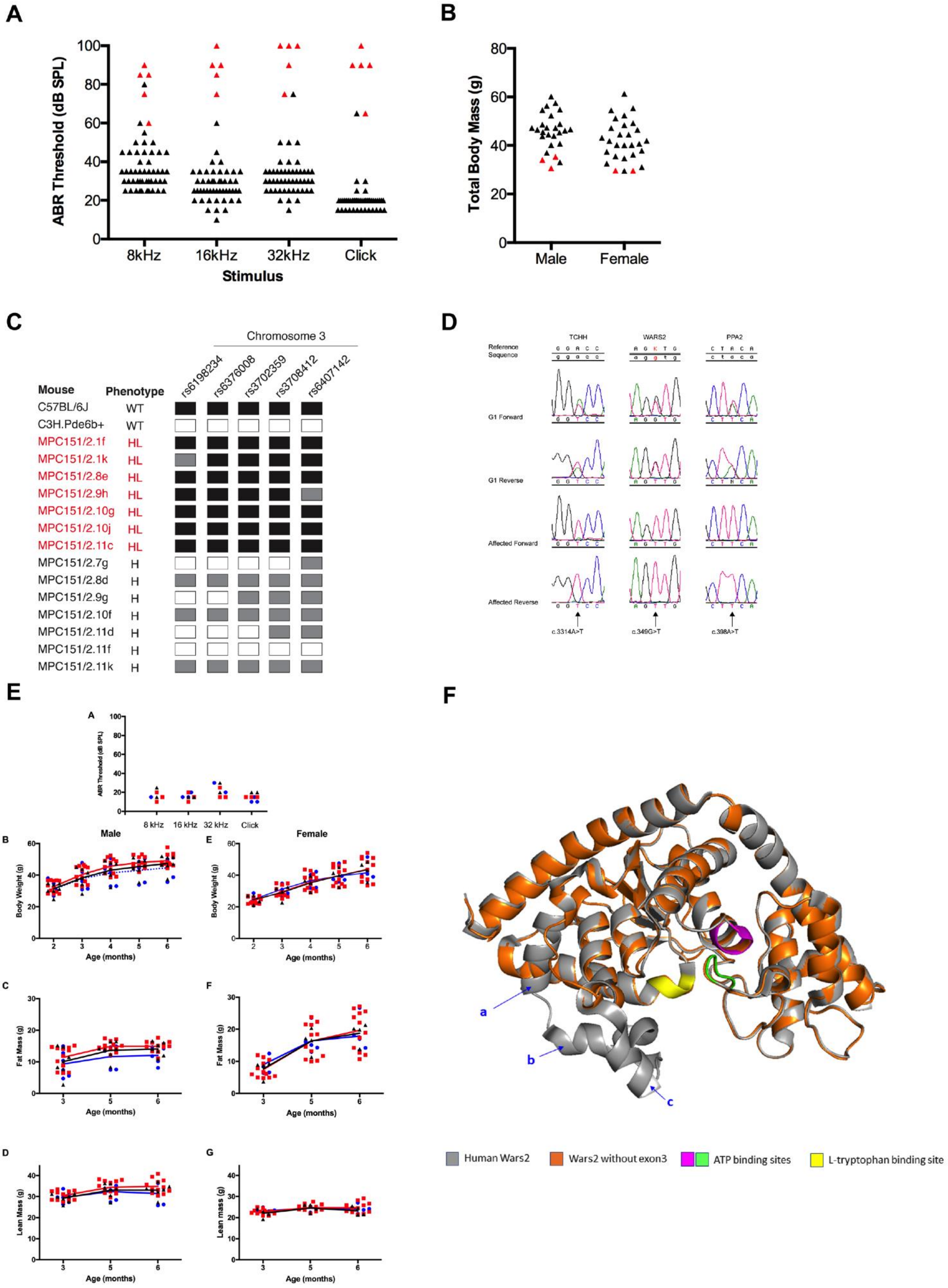

Supplementary Figure 1. Related to Figure 1. Auditory phenotyping, SNP mapping, whole genome sequencing of mouse pedigree MPC151, non-segregation of the phenotype in PP2A mice and the crystal structure of WARS2.

A) Auditory brainstem response (ABR) phenotyping of pedigree MPC151 at 12-months of age showed 5 mice with elevated hearing thresholds (red triangles) at all the frequencies tested (8, 16 and 32 kHz) and for the click stimulus, compared to their normal-hearing littermates (n=48, black triangles).

B) Body weight phenotyping of male and female pedigree MPC-151 at 12-months of age shows that 5 mice with elevated hearing thresholds (male n=3, female n=2, red triangles) also exhibit low body weight, compared to normal-hearing littermates (male n=23, female n=26, black triangles).

C) DNA from the 7 mice exhibiting hearing loss (HL) at 9-months of age and 7 normal-hearing (H) littermates was analyzed by whole genome SNP mapping. The first column indicates the mouse identification numbers and the second column their respective phenotype. The genotype of each mouse is either homozygous for C57BL/6J (black) or C3H (white) or heterozygous (grey) for each marker. The analysis defined a ~73.3Mb critical interval on Chromosome 3 between markers rs6198234 and rs6407142 (Chr3:70361430-143619317, GRCm38).

D) Sanger sequencing of MPC-151 G1 founder and MPC-151 ‘affected’ G3 (MPC151/2.10g) at the shown locations corresponding to the three identified ENU-induced missense mutations on Chr 3: *Tchh*-c.3314A>T, *Wars2*-c.349G>T and *Ppa2*-c.398A>T. The MPC-151 G1 founder is heterozygous for all 3 missense mutations. The MPC-151 ‘affected’ G3 is homozygous for the three missense mutations.

E) Intercross cohort mice segregating the PPA2 Y123F mutation do not exhibit a phenotype. In subpanels (A) ABR at 6 months of age and (D-G) phenotyping over a 6 month time-course in male and female mice respectively for (B,E) body weight, (C,F) fat mass and (D,G) lean mass. In subpanel (A) PPA2$^{Y123F/Y123F}$ n=4, PPA2$^{Y123F/+}$ n=3, PP2A$^{+/+}$ n=2, and in subpanels (B-G) male and female for PPA2$^{Y123F/Y123F}$ n=6 and n=5, for PPA2$^{Y123F/+}$ n=12 and n=12 and for PP2A$^{+/+}$ n=7 and n=4 respectively. Time course data were analyzed with a 2-way ANOVA and Bonferroni correction for multiple testing and all comparisons were non-significant. Homozygous PPA2$^{Y123F/Y123F}$ are blue circles, heterozygous PPA2$^{Y123F/+}$ are red squares and wildtype colony-mates are black triangles.

F) The crystal structure of Human WARS2 protein (pdb id: 5ekd) colored grey aligned to the predicted protein structure of WARS2 *without* exon 3 colored orange. The structural prediction of the deletion of exon 3 shows that three α -helices (a, b & c) are removed in the resulting predicted Wars2 protein structure. The two exon 3 encoded alpha helices are a and b. The catalytic domain, which comprises a Rossmann fold, holds three active sites; Class I PxxxxHIGH and KMSKS active-site catalytic (ATP binding) motifs, colored magenta and green respectively, and the L-tryptophan binding site, in yellow. The catalytic motifs are not directly affected by the mutation. However, these three 3 α –helices are part of the AARs catalytic domain which synthesizes aminoacyl adenylate and moves the amino acid to the anticodon binding domain (not shown).

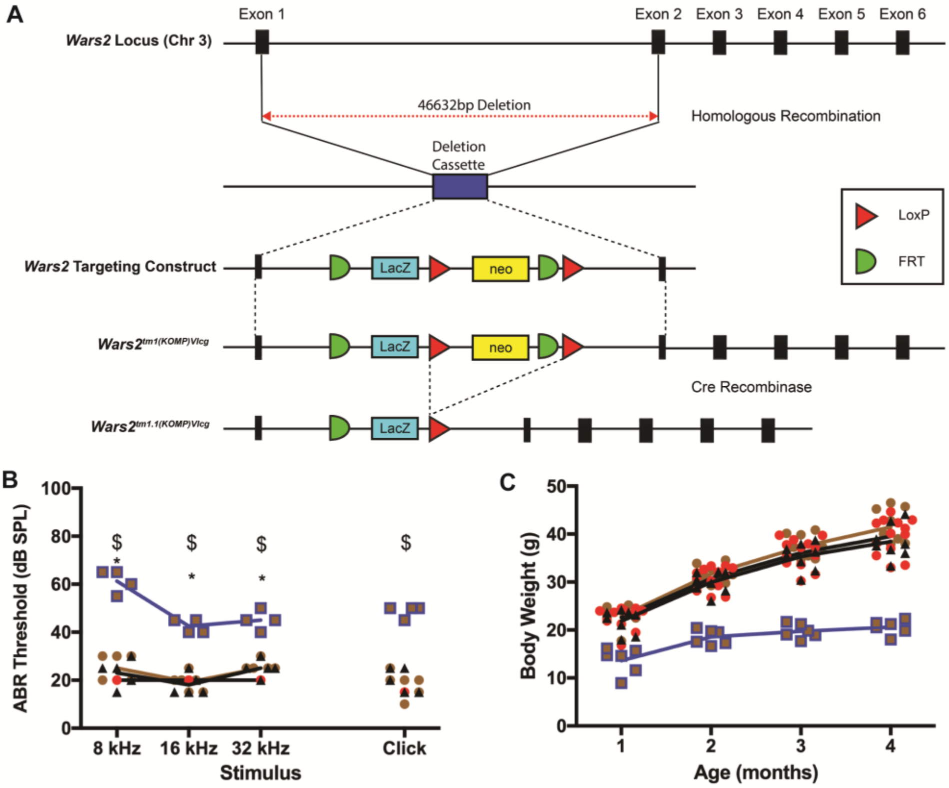

	1 month	2 month	3 month	4 month
Wars2$^{+/+}$ to *Wars2*$^{V117L/-}$	0.0519	0.0554	0.0387	0.0728
Wars2$^{V117L/+}$ to *Wars2*$^{V117L/-}$	0.0026	0.0030	0.0041	0.0043
Wars2$^{+/-}$ to *Wars2*$^{V117L/-}$	0.0016	0.0011	0.0011	0.0004

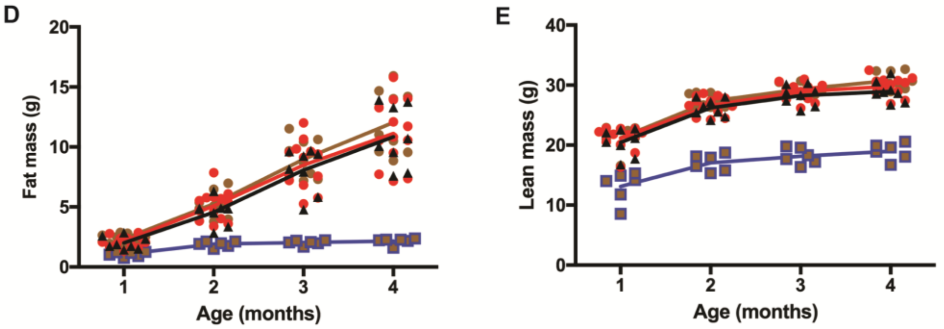

	1 month	2 month	3 month	4 month
Wars2$^{+/+}$ to *Wars2*$^{V117L/-}$	0.053	0.0691	0.0224	0.0285
Wars2$^{V117L/+}$ to *Wars2*$^{V117L/-}$	0.0050	0.0022	0.0041	0.0051
Wars2$^{+/-}$ to *Wars2*$^{V117L/-}$	0.0005	0.0013	0.0021	0.0012

	1 month	2 month	3 month	4 month
Wars2$^{+/+}$ to *Wars2*$^{V117L/-}$	0.0349	0.0649	0.0649	0.1589
Wars2$^{V117L/+}$ to *Wars2*$^{V117L/-}$	0.0022	0.0039	0.0021	0.0027
Wars2$^{+/-}$ to *Wars2*$^{V117L/-}$	0.0031	0.0006	0.0016	0.0003

Supplementary Figure 2. Related to Figure 1. *Wars2$^{V117L/-}$* compound heterozygous knockout phenotyping data.

A) Schematic diagram illustrating the generation of the KOMP *Wars2*-KO allele (*Wars2$^{tm1(KOMP)Vlcg}$*). The targeting construct integrated into the C57BL/6N ES cell genome by homologous recombination, deleting 46632bp of the *Wars2* gene locus, including coding regions of both *Wars2*-Exon1 and *Wars2*-Exon2, leading to a frame-shift and a premature stop codon. *Wars2$^{tm1(KOMP)Vlcg}$* ES cells were micro-injected into C57BL/6N blastocysts generating mosaic C57BL/6N-*Wars2$^{tm1(KOMP)Vlcg}$* offspring. Germ-line transmission (GLT) of the *Wars2$^{tm1(KOMP)Vlcg}$* construct was determined by genotyping C57BL/6N-*Wars2$^{tm1(KOMP)Vlcg}$* x C57BL/6N offspring for the neomycin selection cassette (data not shown). Once GLT was achieved C57BL/6N-*Wars2$^{tm1(KOMP)Vlcg}$* mice were crossed with cre-recombinase expressing mice to remove the neomycin selection cassette. *Wars2$^{+/-}$* indicates heterozygous mice with one wildtype allele (+) and one deleted allele (-).

B) Auditory brainstem response thresholds at 4-months of age were recorded at single frequencies: 8, 16 and 32kHz, and a click stimulus. *Wars2$^{+/+}$*,*Wars2$^{V117L/+}$* (*Tchh$^{D1105V/+}$*), *Wars2$^{+/-}$* and *Wars2$^{V117L/-}$* (*Tchh$^{D1105V/+}$*) animal numbers 5, 1, 6 and 4 respectively. Data were analyzed using a 1-way ANOVA non-parametric Kruskal-Wallis test and Dunns multiple comparison test between *Wars2$^{+/+}$* compared to *Wars2$^{V117L/-}$* (*Tchh$^{D1105V/+}$*) and *Wars2$^{+/-}$* compared to *Wars2$^{V117L/-}$*, (*Tchh$^{D1105V/+}$*) shown as * or \$ P<0.05 respectively. Wildtype colony-mate *Wars2$^{+/+}$* black triangles, heterozygote point-mutation *Wars2$^{V117L/+}$*(*Tchh$^{D1105V/+}$*) red filled circles, heterozygous knockout *Wars2$^{+/-}$* brown filled circle and compound heterozygote *Wars$^{V117L/-}$* blue square filled with brown.

C) Body weight, **D)** Fat mass, **E)** Lean Mass were recorded from male mice 1- to 4-months of age. *Wars2$^{+/+}$*,*Wars2$^{V117L/+}$* (*Tchh$^{D1105V/+}$*), *Wars2$^{+/-}$* and *Wars2$^{V117L/-}$* (*Tchh$^{D1105V/+}$*) animal numbers 8, 13, n=9 and 6 respectively. AUCs were calculated baselined to zero and *Wars2$^{+/+}$*compared to *Wars2$^{V117L/-}$*, *Wars2$^{+/V117L}$* compared to *Wars2$^{V117L/-}$* (*Tchh$^{D1105V/+}$*) and *Wars2$^{+/-}$* compared to *Wars2$^{V117L/-}$* (*Tchh$^{D1105V/+}$*), using a 1-way ANOVA non-parametric Kruskal-Wallis test and Dunns multiple comparison test giving p values of 0.0691, 0.0038 and 0.006 respectively. Significance at specific time-points was calculated with a 1-way ANOVA non-parametric Kruskal-Wallis test and Dunn's multiple comparison test and is shown in table below each figure. Wildtype colony-mate *Wars2$^{+/+}$* black triangles, heterozygote point-mutation *Wars2$^{V117L/+}$*(*Tchh$^{D1105V/+}$*) red filled circles, heterozygous knockout *Wars2$^{+/-}$* brown filled circle and compound heterozygote *Wars$^{V117L/-}$* blue square filled with brown.

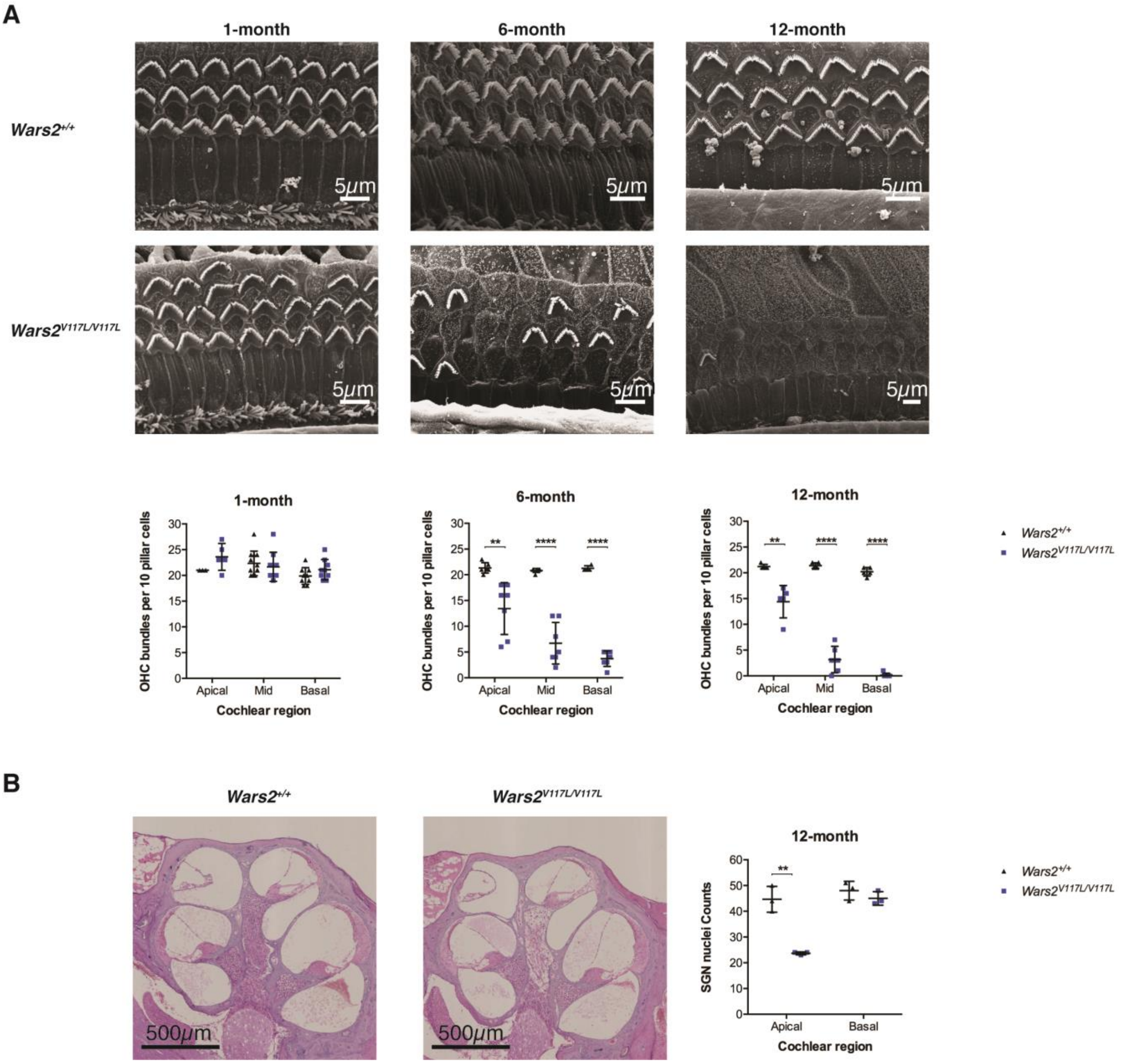

A
1-month
6-month
12-month
Wars2+/+
Wars2V117L/V117L
5μm
OHC bundles per 10 pillar cells
Cochlear region
Apical
Mid
Basal
**
****
B
Wars2+/+
Wars2V117L/V117L
500μm
12-month
SGN nuclei Counts

Supplementary Figure 3. Related to Figure 1. Ultrastructural analyses reveal progressive loss of outer hair cell bundles, and histology shows reduced spiral ganglion neuron number, in *Wars2*$^{V117L/V117L}$ mutant mice.

A) Scanning Electron Micrographs of the mid-coil of the cochlear sensory epithelium from *Wars2*$^{+/+}$ and *Wars2*$^{V117L/V117L}$ mice at 1-, 6-, and 12-months of age. At 1-month of age, the number and appearance of the outer hair cell stereocilia bundles are as expected and similar across genotypes. At 6-months, there is loss of outer hair cell bundles in the *Wars2*$^{V117L/V117L}$ mutant mice, which is not observed in the *Wars2*$^{+/+}$ control mice. By 12-months of age, there is a near complete loss of outer hair cell bundles in the *Wars2*$^{V117L/V117L}$ mutant mice, which is not observed in the *Wars2*$^{+/+}$ control mice. Shown are representative images from the mid region of the cochlear spiral, at least three cochleae from independent mice were imaged per region for each genotype. Scale bar 5µm. To assess the loss of outer hair cell bundles in the apical, mid and basal turns of the cochlear coil counts were undertaken to determine the number of bundles adjacent to ten pillar cells. At 1-month of age *Wars2*$^{+/+}$ (apex n=3, Mid n=12, base n=9) and *Wars2*$^{V117L/V117L}$ (apex n=5, Mid n=9, base n=9) mice have similar numbers of OHC bundles. However, by 6-months of age *Wars2*$^{V117L/V117L}$ (apex n=7, Mid n=7, base n=7) mice have a reduced number of OHC bundles in all cochlear regions compared to *Wars2*$^{+/+}$ (apex n=6, Mid n=6, base n=4) mice. At 12-months of age Wars2$^{V117L/V117L}$ (apex n=5, Mid n=6, base n=7) mice show a further loss of OHC bundles in all cochlear regions compared to *Wars2*$^{+/+}$ (apex n=5, Mid n=5, base n=5) mice. While *Wars2*$^{V117L/V117L}$ mice show a progressive loss of OHC bundles throughout the cochlear spiral, no significant OHC bundle loss is observed in the *Wars2*$^{+/+}$ mice up to 12-months of age. Mean ± SD. Homozygous *Wars2*$^{V117L/V117L}$ are blue squares and *Wars2*$^{+/+}$ black triangles.

B) H&E-stained mid-modiolar cochlear sections from *Wars2*$^{+/+}$ and *Wars2*$^{V117L/V117L}$ mice at 12-months of age. Visual assessment of the sections shows the number of SGN nuclei to be similar from base-to-apex in *Wars2*$^{+/+}$ mice, but there appears fewer nuclei from base-to-apex in *Wars2*$^{V117L/V117L}$ mice. Shown are representative sections from one *Wars2*$^{+/+}$ and one *Wars2*$^{V117L/V117L}$ mouse. Scale bar 500µm. To assess this apparent reduced number of nuclei, counts were undertaken to determine the number of spiral ganglion neuron nuclei within a set 5000µm2 area within the apical and basal turns.This shows that in the basal turn *Wars2*$^{+/+}$ and *Wars2*$^{V117L/V117L}$ mice have comparable numbers of nuclei. In addition, *Wars2*$^{+/+}$ mice have comparable numbers of nuclei in the apical and basal turns. However, *Wars2*$^{V117L/V117L}$ mice do not have comparable numbers of nuclei in the apical and basal turns. Three sections per genotype were used for counts, obtained from independent mice, mean ± SD. Homozygous *Wars2*$^{V117L/V117L}$ are blue squares and *Wars2*$^{+/+}$ black triangles. Significance was determined using an unpaired t test: ** P<0.01, *** P<0.001, **** P<0.0001.

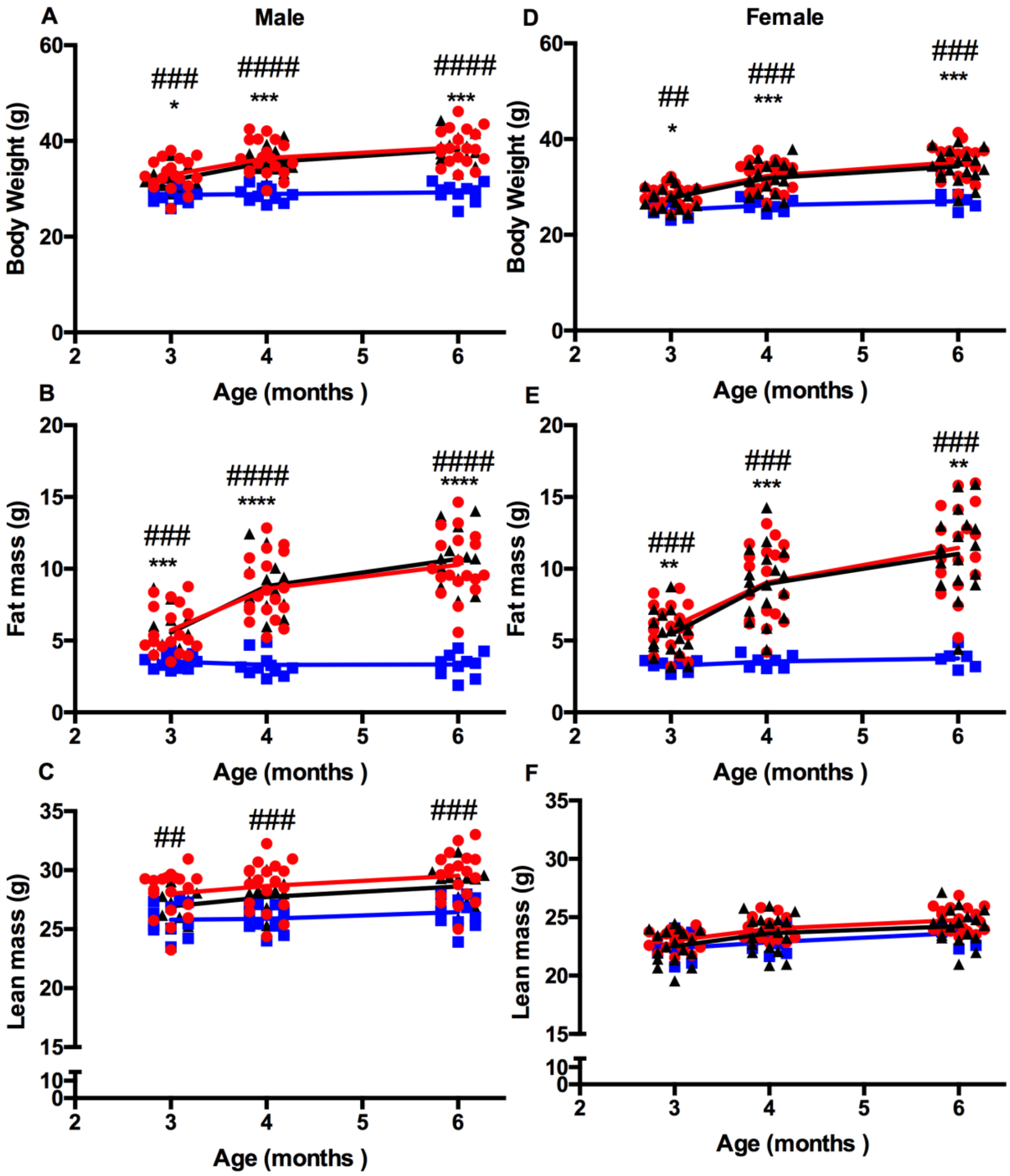
A
Male
D
Female
Body Weight (g)
Age (months)
B
E
Fat mass (g)
C
F
Lean mass (g)
###
*
####
***
##
****
**

Supplementary Figure 4. Related to Figure 2. ***Wars2$^{V117L/V117L}$* mice have reduced body weight due to reduced adiposity.** Male and female **A and D)** Body weight **B and E)** Fat mass and **C and F)** Lean mass in cohort 2 mice. *Wars2$^{V117L/V117L}$*,*Wars2$^{V117L/+}$*, *Wars2$^{+/+}$* animal numbers, male and female, were 11 and 6-7, 19 and 19 and 13 and 18, respectively. AUC calculated with zero baselines and compared using a 1-way ANOVA non-parametric Kruskal-Wallis test and Dunns multiple comparison test. For AUC comparing *Wars2$^{+/+}$* and *Wars2$^{V117L/V117L}$*, *Wars2$^{V117L/+}$* and *Wars2$^{V117L/V117L}$*, and *Wars2$^{+/+}$* and *Wars2$^{V117L/+}$*: Male body weight 0.0003, <0.0001 and >0.999; fat mass <0.0001, <0.0001 and >0.999; lean mass 0.0847, 0.0005 and 0.4131 respectively; Female body weight 0.0020, 0.0005 and >0.999; fat mass 0.0016, 0.0005 and >0.999; lean mass 0.8539, 0.2825 and >0.999 respectively. Significance at specific time-points was also calculated using a 1-way ANOVA non-parametric Kruskal-Wallis test and Dunns multiple comparison test, significance between *Wars2$^{V117L/V117L}$* and *Wars2$^{+/+}$* shown as * P<0.05, ** P<0.01, *** P<0.001, **** P<0.0001 and significant differences between *Wars2$^{V117L/V117L}$* and *Wars2 $^{V117L/+}$*shown as # P<0.05, ## P<0.01, ### P<0.001, #### P<0.0001. Homozygous *Wars2$^{V117L/V117L}$* are blue squares, heterozygous *Wars2$^{V117L/+}$* are red circles and wildtype colony-mate *Wars2$^{+/+}$* black triangles.

Quantification of Western blots at 12 months of age Figure 4 D to J

A

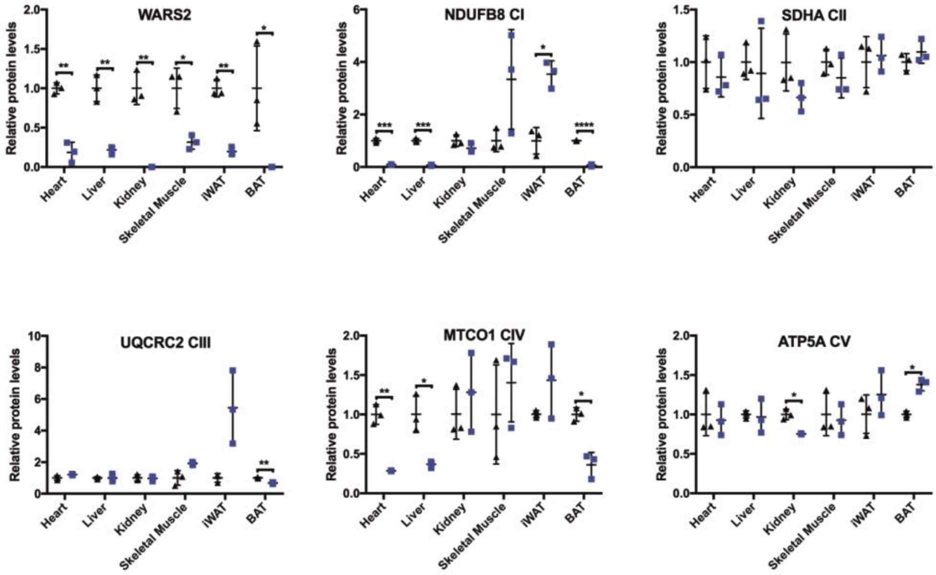

Respiratory chain complex activities

B

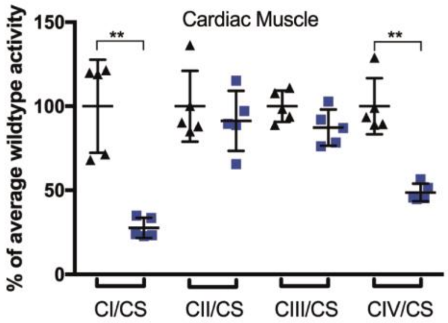

C

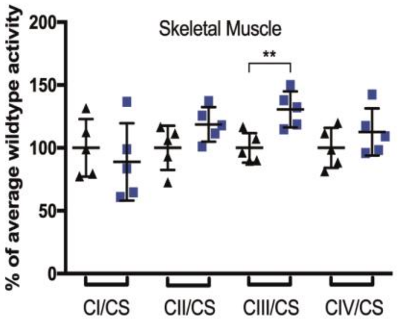

Brain Western blots 3-5 months of age

D

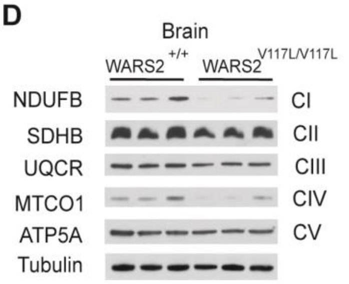

E

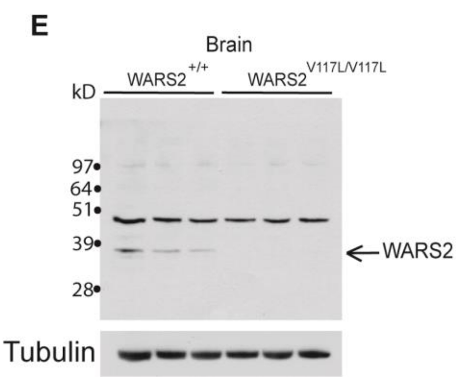

F

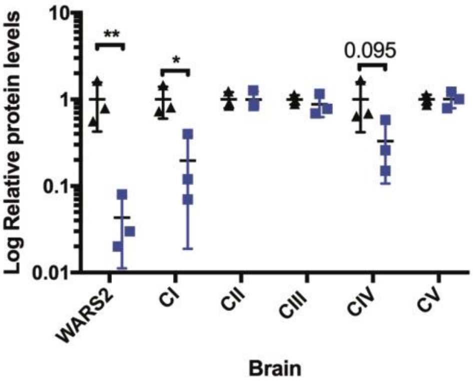

Supplementary Figure 5. Related to Figure 4. (A) Quantification of Western blots from Figures 4 D to J. (B and C) Reduced complex I and IV activities in cardiac muscle and increased CIII activity in skeletal muscle of *Wars2*$^{V117L/V117L}$ mice by 12 months of age. (D, E and F) Reduced WARS2 and complex I and IV deficiencies in whole brain of mice at 3-5 months of age.

A) Quantification of 12 month of age WARS2 and respiratory chain complex subunits in multiple tissues shown in main **Figure 4** western blots. *Wars2*$^{V117L/V117L}$ and *Wars2*$^{+/+}$ animal numbers were 3 each. Statistical analysis was done by multiple t-tests using the Holm-Sidak method and without assuming consistent standard deviation. Adjusted p values are shown * P<0.05, ** P<0.01, *** P<0.001, **** P<0.0001.

B and C) Quantified respiratory chain complex activities in **B)** cardiac muscle and **C)** skeletal muscle, normalized to citrate synthase activity and expressed as a percentage of average wildtype values**.** *Wars2*$^{+/+}$ and *Wars2*$^{V117L/V117L}$ animal numbers 5 each. Cardiac muscle CI and CIV data was analyzed using a Mann-Whitney t-test and all other data with an unpaired two-tailed t-test.

D) Immunoblot analysis of WARS2 and **E)** mitochondrial respiratory chain sub-unit protein levels protein in *Wars2*$^{V117L/V117L}$ *Wars2*$^{+/+}$ (C3H/Pde) whole brain samples from male mice aged between approximately 3 and 5 months of age. *Wars2*$^{V117L/V117L}$ and *Wars2*$^{+/+}$ animal numbers were 3 each. **F)** Quantification of protein blots in **D** and **E**, data plotted as Log10 relative to widltype. For statistical analysis raw data was square root transformed and analyzed by an unpaired t-test for each probe. * P<0.05, ** P<0.01. Homozygous *Wars2*$^{V117L/V117L}$ are shown as blue squares and wildtype colony-mate *Wars2*$^{+/+}$ as black triangles.

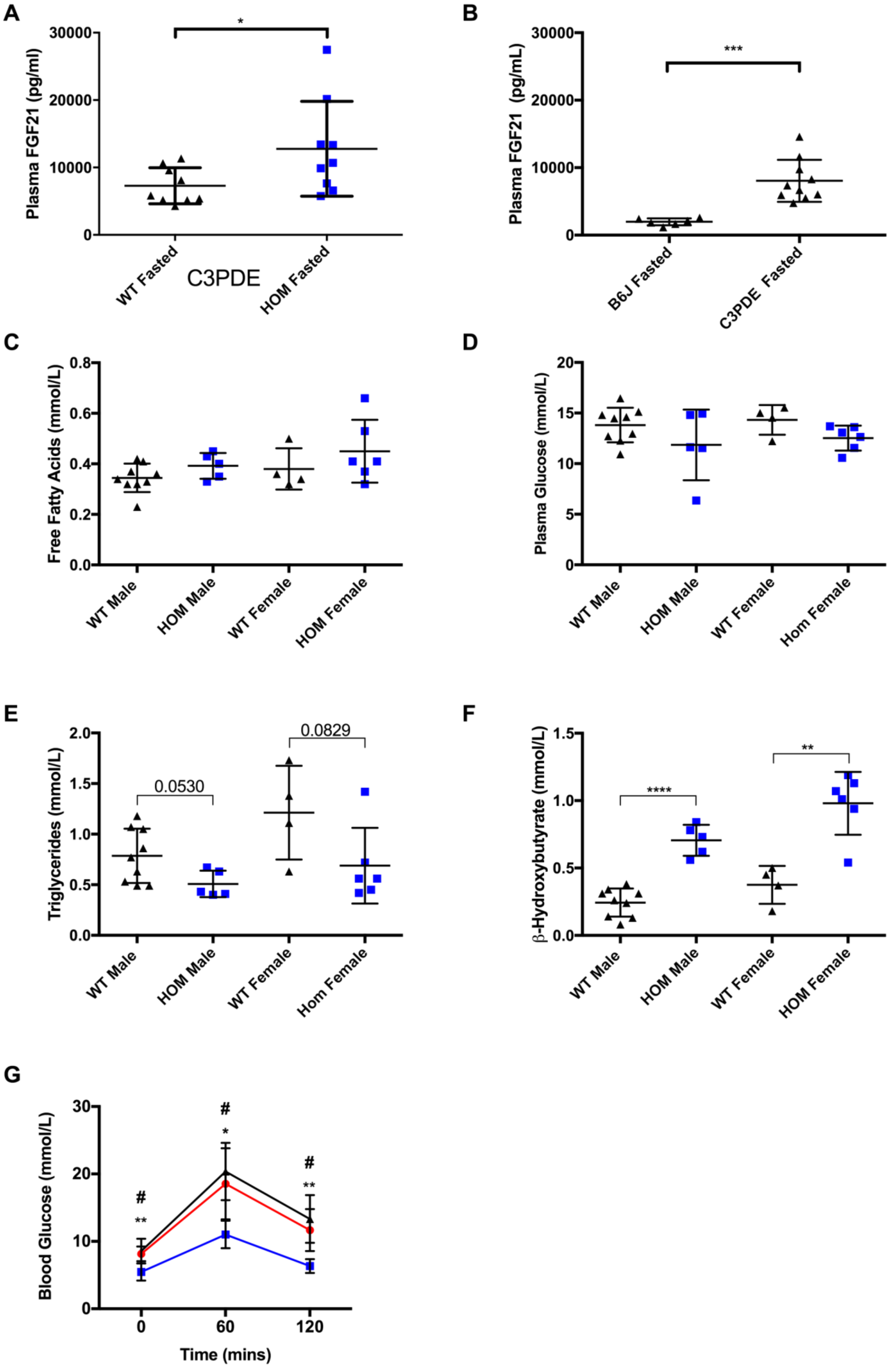
A
Plasma FGF21 (pg/ml)
30000
20000
10000
0
*
WT Fasted
HOM Fasted
C3PDE
B
Plasma FGF21 (pg/mL)
***
B6J Fasted
C3PDE Fasted
C
Free Fatty Acids (mmol/L)
0.8
0.6
0.4
0.2
0.0
WT Male
HOM Male
WT Female
HOM Female
D
Plasma Glucose (mmol/L)
20
15
10
5
0
WT Male
HOM Male
WT Female
Hom Female
E
Triglycerides (mmol/L)
2.0
1.5
1.0
0.5
0.0
0.0530
0.0829
WT Male
HOM Male
WT Female
Hom Female
F
β-Hydroxybutyrate (mmol/L)
1.5
1.0
0.5
0.0
****
**
WT Male
HOM Male
WT Female
HOM Female
G
Blood Glucose (mmol/L)
30
20
10
0
#
**
#
*
#
**
0
60
120
Time (mins)

Supplementary Figure 6. Related to Figure 7. Increased plasma FGF21 is linked with systemic changes in metabolism in *Wars2$^{V117L/V117L}$* mice.

A and B) Plasma FGF21. A) Fasted plasma FGF21 in 4-month old overnight fasted male mice. *Wars2$^{V117L/V117L}$* and *Wars2$^{+/+}$* animal numbers were 9 each, mean ± SD. **B)** Fasted plasma FGF21 in 3-4-month old overnight fasted male mice C57BL/6J (B6J) and C3H/Pde (C3PDE) B6J and C3PDE animal numbers were 6 and 10 respectively, mean ± SD. Significance in **A)** and **B)** calculated using a Mann Whitney 2-tailed t-test, * <0.05 and *** <0.001. Note that the C3PDE background on which the *Wars2^{V117L}* mutation is maintained shows strain specific differences in FGF21 concentration. Homozygous *Wars2$^{V117L/V117L}$* are blue squares and wildtype colony-mate *Wars2$^{+/+}$* black triangles. Significant differences between groups shown as * P<0.05, *** P<0.001.

C, D, E and F) plasma clinical chemistry. C) Free fatty acids (FFA), **D)** glucose, **E)** triacylglycerides (TAG) and **F)** β-hydroxybutyrate levels were analysed in plasma samples collected from *Wars2$^{V117L/V117L}$* and *Wars2$^{+/+}$* mice at 12-months of age. *Wars2$^{V117L/V117L}$* and *Wars2$^{+/+}$* male and female animal numbers were 5, 6 and 9,4 respectively, mean ± SD. Data A and B analysed by Mann-Whitney test and data C and D by unpaired two-tailed t-test. Homozygous *Wars2$^{V117L/V117L}$* are blue squares and wildtype colony-mate *Wars2$^{+/+}$* black triangles. Significant differences between groups shown as * P<0.05, **** P<0.0001

G) Intraperitoneal glucose tolerance tests (IPGTT) were performed in male *Wars2$^{V117L/V117L}$*, *Wars2$^{V117L/+}$* and *Wars2$^{+/+}$* mice at 6-months of age. *Wars2$^{V117L/V117L}$*, *Wars2$^{V117L/+}$* and *Wars2$^{+/+}$* animal numbers were 5,7 and 5 respectively, mean ± SD. IPGTT were performed early in the morning following over-night fasting and tail blood glucose levels were taken 0, 60 and 120 mins after an intraperitoneal glucose injection. AUC was calculated with zero baselines and compared using a one-way ANOVA with Tukey's multiple comparison test. For AUC comparisons between *Wars2$^{+/+}$* and *Wars2$^{V117L/V117L}$*, *Wars2$^{V117L/+}$* and *Wars2$^{V117L/V117L}$, Wars2$^{+/+}$* and *Wars2$^{V117L/+}$,* 0.0143, 0.0474 and 0.6545. Significance at specific time-points was calculated with a 1-way ANOVA with Tukey's multiple comparison test. Significance between *Wars2$^{V117L/V117L}$* and *Wars2$^{+/+}$* shown as * P<0.05, ** P<0.01, and significant differences between *Wars2$^{V117L/V117L}$* and *Wars2$^{V117L/+}$* shown as # P<0.05, ## P<0.01. Homozygous *Wars2$^{V117L/V117L}$* are blue squares, heterozygous *Wars2$^{V117L/+}$* are red circles and wildtype colony-mate *Wars2$^{+/+}$* black triangles.

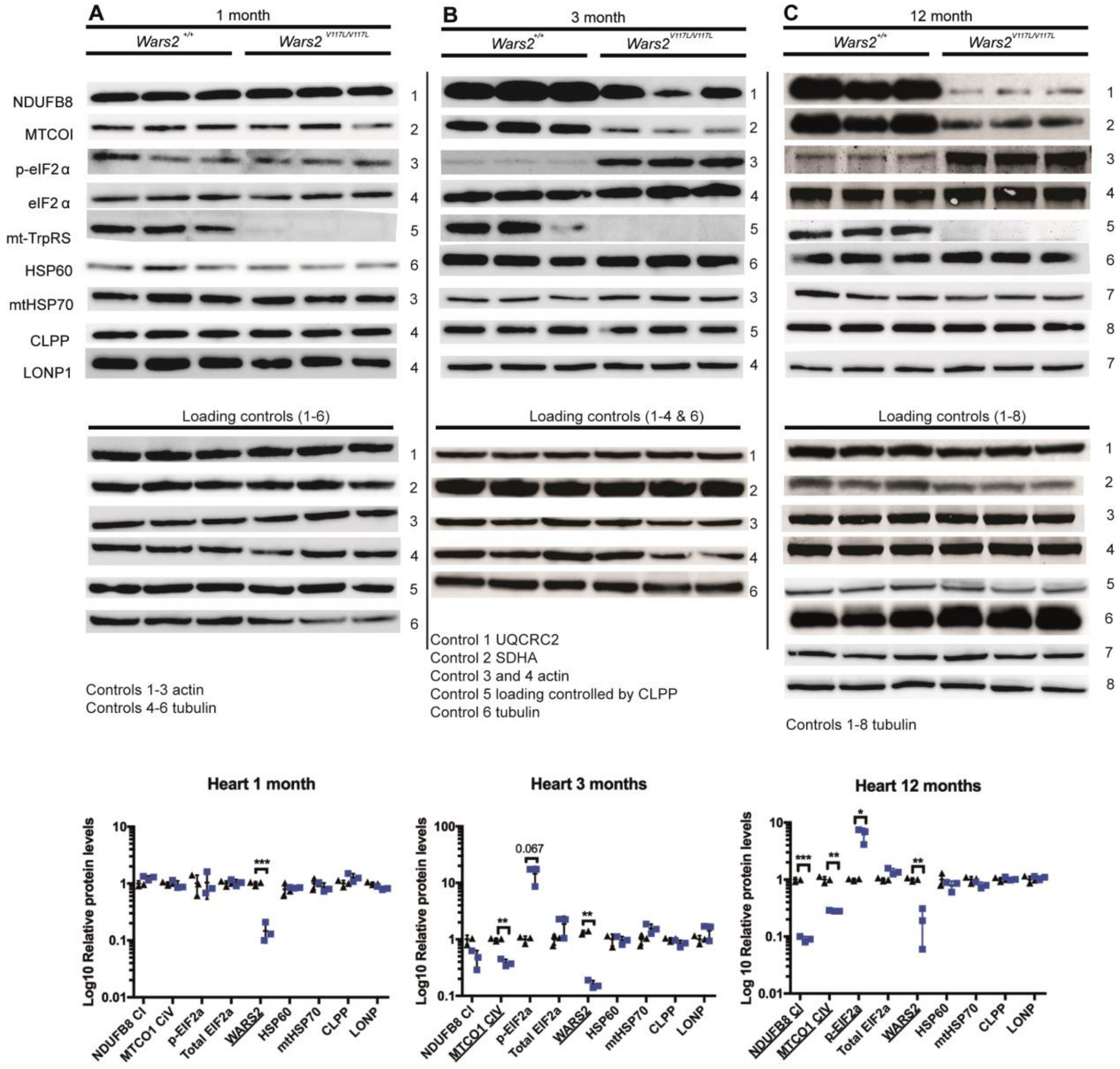
A
1 month
Wars2 +/+
Wars2 V117L/V117L
NDUFB8
MTCOI
p-eIF2α
eIF2α
mt-TrpRS
HSP60
mtHSP70
CLPP
LONP1
Loading controls (1-6)
Controls 1-3 actin
Controls 4-6 tubulin
B
3 month
Loading controls (1-4 & 6)
Control 1 UQCRC2
Control 2 SDHA
Control 3 and 4 actin
Control 5 loading controlled by CLPP
Control 6 tubulin
C
12 month
Loading controls (1-8)
Controls 1-8 tubulin
Heart 1 month
Heart 3 months
Heart 12 months
Log10 Relative protein levels
Log 10 Relative protein levels
0.067
NDUFB8 CI
MTCO1 CIV
p-EIF2a
Total EIF2a
WARS2
HSP60
mtHSP70
CLPP
LONP

Supplementary Figure 7. Related to Figure 7. Activation of the ISR is progressive with age, co-incident with mitochondrial respiratory chain deficiencies and independent of disrupted mitochondrial proteostasis in *Wars2*$^{V117L/V117L}$ heart. Immunoblot analysis of LONP1, CLPP, mtHsp70, mtHsp60, p-eIF2α, (total)eIF2α, COXI (CIV) and NDUFB8 (CI) protein levels in *Wars2*$^{V117L/V117L}$ and *Wars2*$^{+/+}$ heart tissue collected from female mice at **A)** 1-month, **B)** 3-months and **C)** 12-months of age. *Wars2*$^{V117L/V117L}$ and *Wars2*$^{+/+}$ animal numbers were 3 each. Note that some 12-month blots are reproduced here from display figures for ease of comparison. The controls for each gel are indicate by numbers on the right of each panel and then shown in the loading control below each panel, note that some blots are re-probed and therefore share controls. Quantification plots are shown directly below the corresponding western blot panels. Statistical analysis was done by multiple t-test (PRISM) using the Holm-Sidak method and without assuming consistent standard deviation. Adjusted p values are shown * P<0.05, ** P<0.01, *** P<0.001. Homozygous *Wars2*$^{V117L/V117L}$ are shown as blue squares and wildtype colony-mate *Wars2*$^{+/+}$ as black triangles.

Supplementary Table 1, Related to Figure 2.

***Wars2$^{-/-}$* causes embryonic lethality.** Number of mice born per genotype from three inter-crosses: **A)** *Wars2$^{V117L/+}$* x *Wars2$^{+/-}$*, **B)** *Wars2$^{V117L/+}$* x *Wars2$^{V117L/+}$* and **C)** *Wars2$^{+/-}$* x *Wars2$^{+/-}$*. Data were analysed for deviance from expected Hardy-Weinberg ratios using a Chi-squared test.

**A) Inter-cross of *Wars2$^{V117L/+}$* x *Wars2$^{+/-}$* mice**		
**Genotype**	**Expected**	**Observed**
Wars2$^{+/+}$	19.5	23
Wars$^{V117L/+}$	19.5	26
Wars2$^{+/-}$	19.5	21
Wars$^{V117L/-}$	19.5	8
Total	78	78

Chi-squared test p=0.1103

**B) Inter-cross of *Wars2$^{V117L/+}$* x *Wars2$^{V117L/+}$* mice**		
**Genotype**	**Expected**	**Observed**
Wars2$^{+/+}$	51.5	56
Wars$^{V117L/+}$	103	101
Wars$^{V117L/V117L}$	51.5	49
Total	*206*	*206*

Chi-squared test p=0.8647

**C) Inter-cross of *Wars2$^{+/-}$* x *Wars2$^{+/-}$* mice**		
**Genotype**	**Expected**	**Observed**
Wars2$^{+/+}$	44.5	53
Wars$^{+/-}$	89	125
Wars$^{-/-}$	44.5	**0**
Total	*178*	*178*

Chi-squared test p=<0.0001

Supplementary Table 2, Related to Star Methods

Oligonucleotide primer sequences

**Primers for genotyping**	
**Name**	**Sequence**
CTCAATCCCATTAAGCAAGATAT	Ppa2^{A398T} primers: biotinylated forward
GGTTTCTGTAGAAGGCATAAAAG	Ppa2^{A398T} primers: biotinylated reverse
GGGAAGATGTTCGGTG	Ppa2^{A398T} primers: sequencing reverse
GGTCACCTTTCTTTCTCTCC	Wars2^{G349T} primers: forward
CAGGTGAGGATCCAACTTAA	Wars2^{G349T} primers: reverse
TTTCTCTCCTTCCTTTTAG	Wars2^{G349T} primers: forward sequencing
TCAGCCTATCCCTGTTGTCTA	*Wars2*^{V117L} Primers: forward
TGGTGTAAATGCTGCAATCG	*Wars2*^{V117L} Primers: reverse
CCTTCCTTTTAGTTGTCTGAACACACTCAG	*Wars2*^{V117L} Primers: probe
GCCCAGCACTTGGGATGT	Wars2^{WT} primers: forward
GCAGCCAGCTCACCAATG	Wars2^{WT} primers: reverse
TCCCTTCACTTTCCTGTCTCCGTTTC	Wars2^{WT} primers: FAM labeled probe
CTCGCCACTTCAACATCAAC	LacZ primers: forward
TTATCAGCCGGAAAACCTACC	LacZ primers: reverse
TCGCCATTTGACCACTACCATCAATCC	LacZ primers: FAM labelled probe
GCCCCAGCACGACCATT	Dot1l primers: forward
TAGTTGGCATCCTTATGCTTCATC	Dot1l primers: reverse
CCAGCTCTCAAGTCG	Dot1l primers: VIC labelled probe
