# Supplementary material for: A Wars2 Mutant Mouse Model Displays OXPHOS Deficiencies and Activation of Tissue-Specific Stress Response Pathways
Source: Cell Rep. 2018 Dec 18;25(12):3315–3328.e6. doi: 10.1016/j.celrep.2018.11.080 (PMC6315286; doi:10.1016/j.celrep.2018.11.080)
Supplement: Document S2. Article plus Supplemental Information [file mmc2.pdf]

Cell Reports

Article

A Wars2 Mutant Mouse Model Displays OXPHOS Deficiencies and Activation of Tissue-Specific Stress Response Pathways

Graphical Abstract

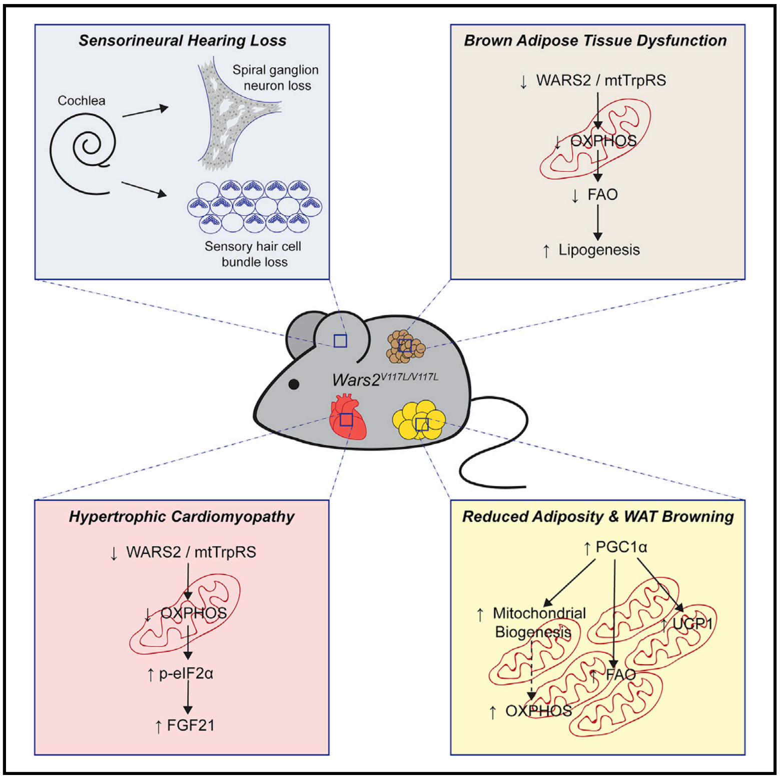

Highlights

- A hypomorphic point mutation in the *Wars2* gene was identified
- Mutant mice exhibit progressive tissue-specific pathologies
- Variable activation of stress response pathways
- Demonstrating pleiotropic effects

Authors

Thomas Agnew, Michelle Goldsworthy, Carlos Aguilar, ..., Robert W. Taylor, Michael R. Bowl, Roger D. Cox

Correspondence

m.bowl@har.mrc.ac.uk (M.R.B.),
r.cox@har.mrc.ac.uk (R.D.C.)

In Brief

A reduced-function mutation in the nuclear-encoded, mitochondrial-localized *Wars2* gives rise to deafness, reduced and abnormal fat, and hypertrophic cardiomyopathy. Agnew et al. show that the different tissue effects of this mutation arise from variable activation of stress response pathways and tissue-specific responses to impaired mitochondrial function.

Agnew et al., 2018, Cell Reports *25*, 3315–3328
December 18, 2018 © 2018 The Author(s).
https://doi.org/10.1016/j.celrep.2018.11.080

OPEN ACCESS
CellPress

A Wars2 Mutant Mouse Model Displays OXPHOS Deficiencies and Activation of Tissue-Specific Stress Response Pathways

Thomas Agnew,[1] Michelle Goldsworthy,[1] Carlos Aguilar,[1] Anna Morgan,[1] Michelle Simon,[1] Helen Hilton,[1] Chris Esapa,[1] Yixing Wu,[1] Heather Cater,[1] Liz Bentley,[1] Cheryl Scudamore,[1] Joanna Poulton,[2] Karl J. Morten,[2] Kyle Thompson,[3] Langping He,[3] Steve D.M. Brown,[1] Robert W. Taylor,[3] Michael R. Bowl,[1,4,*] and Roger D. Cox[1,4,5,*]

[1]MRC Harwell Institute, Mammalian Genetics Unit and Mary Lyon Centre, Harwell Campus, Oxfordshire OX11 0RD, UK
[2]Nuffield Department of Obstetrics and Gynaecology, University of Oxford, Level 3 The Women's Centre, John Radcliffe Hospital, Headington, Oxford OX3 9DU, UK
[3]Wellcome Centre for Mitochondrial Research, Institute of Neuroscience, The Medical School, Newcastle University, Newcastle upon Tyne NE2 4HH, UK
[4]These authors contributed equally
[5]Lead Contact
*Correspondence: m.bowl@har.mrc.ac.uk (M.R.B.), r.cox@har.mrc.ac.uk (R.D.C.)
https://doi.org/10.1016/j.celrep.2018.11.080

SUMMARY

Mutations in genes essential for mitochondrial function have pleiotropic effects. The mechanisms underlying these traits yield insights into metabolic homeostasis and potential therapies. Here we report the characterization of a mouse model harboring a mutation in the tryptophanyl-tRNA synthetase 2 (*Wars2*) gene, encoding the mitochondrial-localized WARS2 protein. This hypomorphic allele causes progressive tissue-specific pathologies, including hearing loss, reduced adiposity, adipose tissue dysfunction, and hypertrophic cardiomyopathy. We demonstrate the tissue heterogeneity arises as a result of variable activation of the integrated stress response (ISR) pathway and the ability of certain tissues to respond to impaired mitochondrial translation. Many of the systemic metabolic effects are likely mediated through elevated fibroblast growth factor 21 (FGF21) following activation of the ISR in certain tissues. These findings demonstrate the potential pleiotropy associated with *Wars2* mutations in patients.

INTRODUCTION

Mitochondrial diseases are a heterogeneous group of disorders caused by mutations in mitochondrial proteins encoded by either the mitochondrial genome (mtDNA) or the nuclear genome (genomic DNA [gDNA]). The nuclear-encoded mitochondrial aminoacyl-tRNA synthetase (mt-aaRS) proteins catalyze the aminoacylation of mitochondrial tRNAs with their cognate amino acid. Mitochondrial tRNA aminoacylation is fundamental to mitochondrial translation and synthesis of mtDNA-encoded respiratory chain subunits and the supply of ATP to the cell. The mt-aaRS proteins are encoded by separate nuclear genes with the exception of glycine- and lysine-tRNA synthetase (*GARS* and *KARS*), which function in both the mitochondria and the cytoplasm. With the description of families with compound heterozygous variants in the tryptophanyl-tRNA synthetase 2 (*WARS2*) gene (Burke et al., 2018; Musante et al., 2017; Theisen et al., 2017; Vantroys et al., 2018; Wortmann et al., 2017), patients have been reported with biallelic, pathogenic mutations in all 19 nuclear-encoded mt-aaRS genes (Oprescu et al., 2017).

Surprisingly, given their common function within mitochondrial translation and ubiquitous expression, mt-aaRS mutations cause distinct tissue-specific pathologies and respiratory chain deficiencies in a gene-dependent manner (Konovalova and Tyynismaa, 2013). For example, pathogenic histidyl-tRNA synthetase 2 (*HARS2*) (Pierce et al., 2011) and leucyl-tRNA synthetase 2 (*LARS2*) (Soldà et al., 2016) mutations cause Perrault syndrome (sensorineural hearing loss and ovarian dysgenesis), glutamyl-tRNA synthetase 2 (*EARS2*) (Steenweg et al., 2012) mutations cause leukoencephalopathy with thalamus and brainstem involvement with high lactate (LTBL), and seryl-tRNA synthetase 2 (*SARS2*) (Belostotsky et al., 2011; Rivera et al., 2013) mutations cause hyperuricemia, pulmonary hypertension, renal failure, and alkalosis (HUPRA) syndrome with hypertrophic cardiomyopathy. The underlying mechanisms dictating the pleiotropic effects and tissue-specific penetrance and variability among individuals with mt-aaRS mutations are unknown and are a major challenge in the understanding and developing therapies for mitochondrial disease (Nunnari and Suomalainen, 2012).

Global mt-aaRS knockout animal models are heterozygous haploinsufficient and homozygous lethal (http://www.mousephenotype.org/) (Dickinson et al., 2016). A heart and skeletal muscle-specific aspartyl-tRNA synthetase 2 (*Dars2*) knockout (Dars2-KO^{Ckmm}) mouse with fatal cardiomyopathy has been reported (Dogan et al., 2014). Complete loss of *Dars2* function caused disrupted mitochondrial proteostasis and activating transcription factor 4 (ATF4)-dependent fibroblast growth factor 21 (FGF21) expression specifically in the heart, but not in skeletal muscle, suggesting tissue-specific differences in mitochondrial proteostatic buffering capacity. However, residual mt-aaRS activity is retained in human patients with mt-aaRS

This is an open access article under the CC BY license (http://creativecommons.org/licenses/by/4.0/).

mutations; thus, animal models with global-hypomorphic mt-aaRS alleles are vital to investigating tissue-specific penetrance.

Hypomorphs from *N*-ethyl-*N*-nitrosourea (ENU) mutagenesis screens in the mouse allow the pleiotropic effects of a mutation to be identified. We have incorporated aging as a sensitizing factor to assess recessive pedigrees for late-onset and progressive phenotypes in metabolism and other body systems (Potter et al., 2016). We report here the identification of an ENU-induced mouse mutant harboring a recessive hypomorphic point mutation in the *Wars2* gene, *Wars2*^{V117L}, which causes a complex tissue-specific pathology, including hearing loss, reduced adiposity, adipose tissue dysfunction, and hypertrophic cardiomyopathy. We demonstrate that reduced WARS2 levels causes tissue-specific respiratory chain deficiencies, modeling human mt-aaRS patients. We demonstrate that tissue-specific upregulation of mitochondrial biogenesis is coincident with respiratory chain deficiencies in *Wars2*$^{V117L/V117L}$ mice, likely contributing to the tissue-specific respiratory chain deficiencies observed. We also show that activation of the integrated stress response (ISR) is a heart-specific response to inhibition of mitochondrial translation that contributes to increased FGF21 levels and systemic changes in metabolism.

RESULTS

We applied high-throughput broad-based phenotyping to pedigrees of mutagenized mice to investigate the pleiotropic effects of the mutations identified (Potter et al., 2016).

Wars2-V117L ENU-Induced Mutation Causal for Hearing Loss and Reduced Adiposity

Auditory phenotyping of one of these pedigrees (MPC151) identified progressive hearing loss. At 3 months of age, all mice displayed a normal response to a clickbox stimulus. However, at 6 months of age, 2 of 58 mice had a reduced response, increasing to 7 animals (12%) by 9 months of age. At 12 months of age, the pedigree was assessed using auditory brainstem response (ABR) testing, which showed that 5 of the 53 surviving mice exhibited elevated hearing thresholds at all frequencies tested (Figure S1A). In addition, the hearing-impaired mice were found to have reduced body weight (Figure S1B).

A genome scan of G_3 mice showed linkage to a ~73.3 Mb region on chromosome 3 containing 1,298 genes (Figure S1C). DNA from an affected G_3 mouse (MPC151/2.10 g) underwent whole-genome sequencing, and analysis of the data identified only three high-confidence non-synonymous coding changes within the mapped interval. These consisted of Chr3:93446568A>T at nucleotide 3314 of the trichohyalin (*Tchh*) gene (Ensembl: ENSMUST00000064257), causing an aspartate-to-valine substitution at residue 1105 (*Tchh*^{D1105V}); Chr3:99204536G>T at nucleotide 349 of the *Wars2* gene (Ensembl: ENSMUST00000004343), causing a valine-to-leucine substitution at residue 117 (*Wars2*^{V117L}); and Chr3:133330454A>T at nucleotide 368 of the pyrophosphatase (inorganic) 2 (*Ppa2*) gene (Ensembl: ENSMUST00000029644), causing a tyrosine-to-phenylalanine substitution at residue 123 (*Ppa2*^{Y123F}). The presence of the three lesions was confirmed using Sanger sequencing, and only mice showing hearing impairment were homozygous for these ENU-induced lesions (Figure S1D).

To segregate the mutations, the offspring were backcrossed for three generations to C3H.Pde6b+ mice. The *Ppa2*^{Y123F} allele was segregated from the *Tchh*^{D1105V} and *Wars2*^{V117L} alleles at backcross 2. However, the *Tchh*^{D1105V} and *Wars2*^{V117L} alleles remained linked due to their proximity. Auditory phenotyping of *Ppa2*$^{Y123F/Y123F}$ mice at 6 months of age showed they had similar ABR thresholds to their wild-type and heterozygous littermates (Figure S1E, A). In addition, the body, fat, and lean mass of animals for each genotype were not significantly different (Figure S1E, B–G). Thus, the *Ppa2*^{Y123F} lesion was excluded as being causative of the phenotypes.

The *Tchh* gene encodes a protein for hair shaft formation, and a patient with a homozygous nonsense *TCHH* mutation and uncombable hair syndrome has been described (Ü Basmanav et al., 2016). We did not observe a hair phenotype in *Wars2*$^{V117L/V117L}$ mice. However, to determine which of the two lesions, *Wars2*^{V117L} or *Tchh*^{D1105V}, is causal, we undertook a genetic complementation test, crossing *Wars2*$^{V117L/+}$:*Tchh*$^{D1105V/+}$ mice with mice heterozygous for a *Wars2* knockout (*Wars2*$^{+/-}$) allele (Figure S2A). This generated offspring that are compound heterozygotes for *Wars2*, but heterozygous for *Tchh* (*Wars2*$^{V117L/-}$:*Tchh*$^{D1105V/+}$). These mice displayed elevated ABR thresholds at 4 months of age and reduced weight, total fat, and lean mass compared to their colony mates, which had normal hearing and weight (*Wars2*$^{+/+}$:*Tchh*$^{+/+}$, *Wars2*$^{V117L/+}$: *Tchh*$^{D1105V/+}$, and *Wars2*$^{+/-}$:*Tchh*$^{+/+}$) (Figures S2B–S2E). Failure of the *Wars2* alleles to complement confirms the *Wars2*^{V117L} lesion as the causal mutation underlying the observed phenotypes. Homozygous null (*Wars2*$^{-/-}$) mice were embryonic lethal, and *Wars2*$^{V117L/-}$ and *Wars2*$^{V117L/V117L}$ mice were subviable and viable, respectively; thus, the *Wars2*^{V117L} allele is hypomorphic, rather than a complete loss of function (Table S1).

To further characterize the phenotypes and establish underlying mechanisms, we bred additional cohorts of mice.

Hearing Loss in *Wars2*$^{V117L/V117L}$ Mice Was Progressive

To investigate progression of the auditory phenotype, ABR was measured at 1, 3, 6, 10, and 12 months of age. The hearing thresholds of *Wars2*$^{+/+}$ and *Wars2*$^{+/V117L}$ mice were comparable and within the normal range at all ages tested. In contrast, *Wars2*$^{V117L/V117L}$ mice display an age-related increase in hearing thresholds at all tested frequencies (Figure 1). Investigation of the cochlear sensory epithelia using scanning electron microscopy showed a progressive loss of outer hair cell stereocilia bundles in the homozygous mutants, with an apical-to-basal increase in severity (Figure S3A). In addition, assessment of cochlear histological sections identified a reduced number of spiral ganglion neurons in the cochlear apex of 12-month-old mutant mice (Figure S3B). The mutant mice showed no overt vestibular dysfunction (e.g., circling, head bob, or abnormal swim), and no craniofacial dysmorphology was observed.

Wars2$^{V117L/V117L}$ Mice Failed to Gain Fat Mass

To refine the reduced body weight phenotype, we analyzed body composition at monthly intervals and found reduced total body weight from 2 months of age, reduced fat mass from 2 months

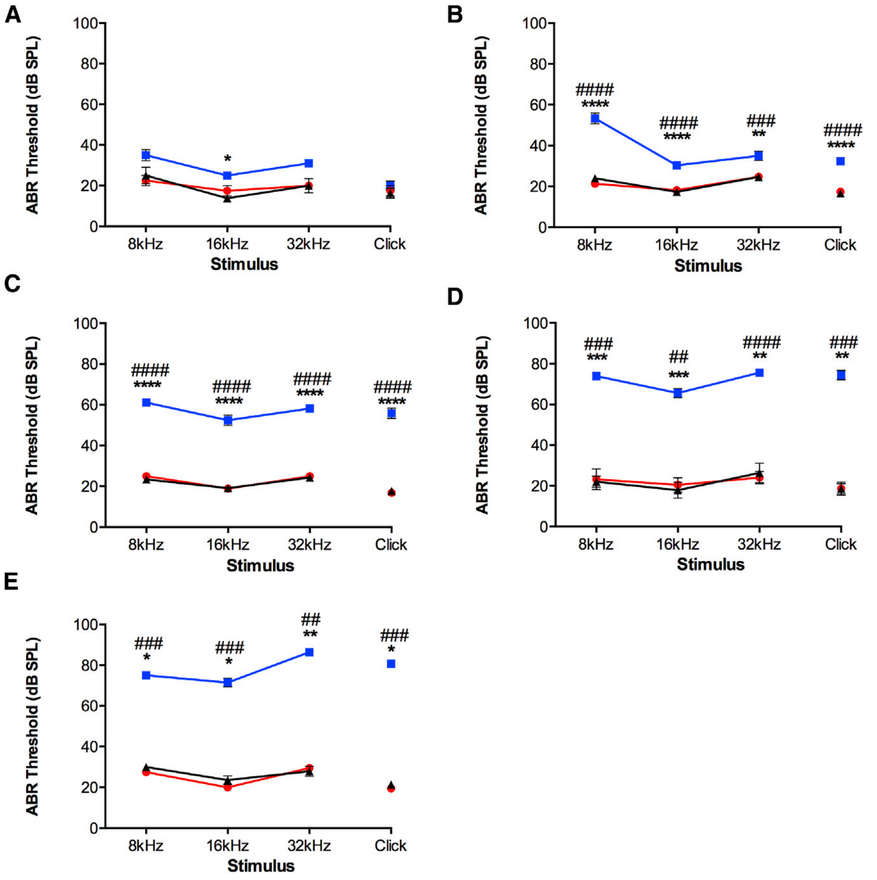

Figure 1. ABR Phenotyping

Minimum auditory detection thresholds (decibel sound pressure level, dB SPL) were determined using auditory brainstem response (ABR) at (A) 1 month, (B) 3 months, (C) 6 months, (D) 10 months, and (E) 12 months of age. *Wars2*$^{V117L/V117L}$, *Wars2*$^{V117L/+}$, and *Wars2*$^{+/+}$ littermate numbers (males and females pooled) were (A) 5, 2, and 4; (B) 15, 19, and 15; (C) 19, 19, and 22; (D) 9, 11, and 7; and (E) 7, 10, and 7, respectively; mean ± SEM. Significance was determined using a one-way ANOVA Kruskal-Wallis test with Dunn's multiple comparisons test. Significance between *Wars2*$^{V117L/V117L}$ and *Wars2*$^{+/+}$ and between *Wars2*$^{+/V117L}$ and *Wars2*$^{+/+}$ is shown as * and #p < 0.05, ** and ##p < 0.01, *** and ###p < 0.001, and **** and ####p < 0.0001, respectively. *Wars2*$^{V117L/V117L}$ mice are blue squares, *Wars2*$^{V117L/+}$ mice are red circles, and *Wars2*$^{+/+}$ mice are black triangles. See also Figures S1–S3 and Table S1.

(female) or 3 months (male) of age, and lean mass from 3 months (male) or 5 months (female, in cohort 1 only) of age (Figures 2A–2C, male; Figures 2D–2F, female cohort 1; Figures S4A–S4C, male; Figures S4D–S4F, female cohort 2). Thus, demonstration of the reduction in total mass was primarily due to decreased adiposity and a failure to increase fat mass. We further investigated whether these differences were the result of specific organ weight changes in *Wars2*$^{V117L/V117L}$ mice dissected at 6 months of age. Visceral gonadal white adipose tissue (gWAT), subcutaneous inguinal WAT (iWAT), and brown adipose tissue (BAT) normalized to body weight were all significantly reduced in *Wars2*$^{V117L/V117L}$ mice compared to wild-type colony mates (Figure 3A), consistent with reduced adiposity. Strikingly, heart weight was increased in *Wars2*$^{V117L/V117L}$ mice (Figure 3A). No significant differences in liver or kidney weight (Figure 3A) were observed, demonstrating organ specificity and that the changes in adipose tissues and heart weight were not because of global growth or development impairment.

Wars2$^{V117L/V117L}$ Mice Showed Hypertrophic Cardiomyopathy

To determine the cause of increased heart weight, *Wars2*$^{V117L/V117L}$ cardiac morphology was assessed by echocardiogram at 5 months of age (Figure 3B). We found significantly increased left ventricular anterior wall (LVAW) diameter and left ventricular (LV) mass in *Wars2*$^{V117L/V117L}$ mice relative to wild-type colony mates, showing that the increase in heart weight was due to hypertrophic cardiomyopathy (Figure 3C). Consistent with this, the LV stroke volume (SV) and cardiac output (CO) were significantly reduced (Figure 3C). These differences were also observed in compound heterozygote *Wars2*$^{V117L/-}$ mice, which showed increased LVAW and LV mass and decreased CO relative to *Wars2*$^{+/+}$, *Wars2*$^{+/V117L}$, and *Wars2*$^{+/-}$ mice at the same age, regardless of *Tchh* genotype (Figure 3D), confirming that the *Wars2*^{V117L} allele was the causal mutation for hypertrophic cardiomyopathy.

Wars2$^{V117L/V117L}$ Mice Did Not Show Gross Brain Pathology

We carried out additional pathology screens to investigate whether there were neurological abnormalities, as reported in patients. On light microscopic examination of the brain (multiple sections of cerebrum and cerebellum) (data not shown) there were no detectable morphological differences between homozygote and wild-type animals (n = 3 of each) of the same age (approximately 7 months) and sex (male). In particular, there was no evidence of myelin deficits. There was also no evidence from visual welfare observation of an *in vivo* neurological phenotype (seizures, tremors, or changes in locomotion), the detection of which often precedes detectable morphological changes at the light-microscopy level. Detection of potential subtle neurological changes would require behavioral or neurophysiological testing, which was beyond the scope of this study.

The *Wars2* c.349G>T Mutation Disrupted Exon Splicing and Caused Tissue-Specific WARS2 Deficiencies

The *Wars2* c.349G>T lesion causes a missense substitution (p.V117L) in the encoded protein. *In silico* prediction of the functional effects of the p.V117L missense substitution did not

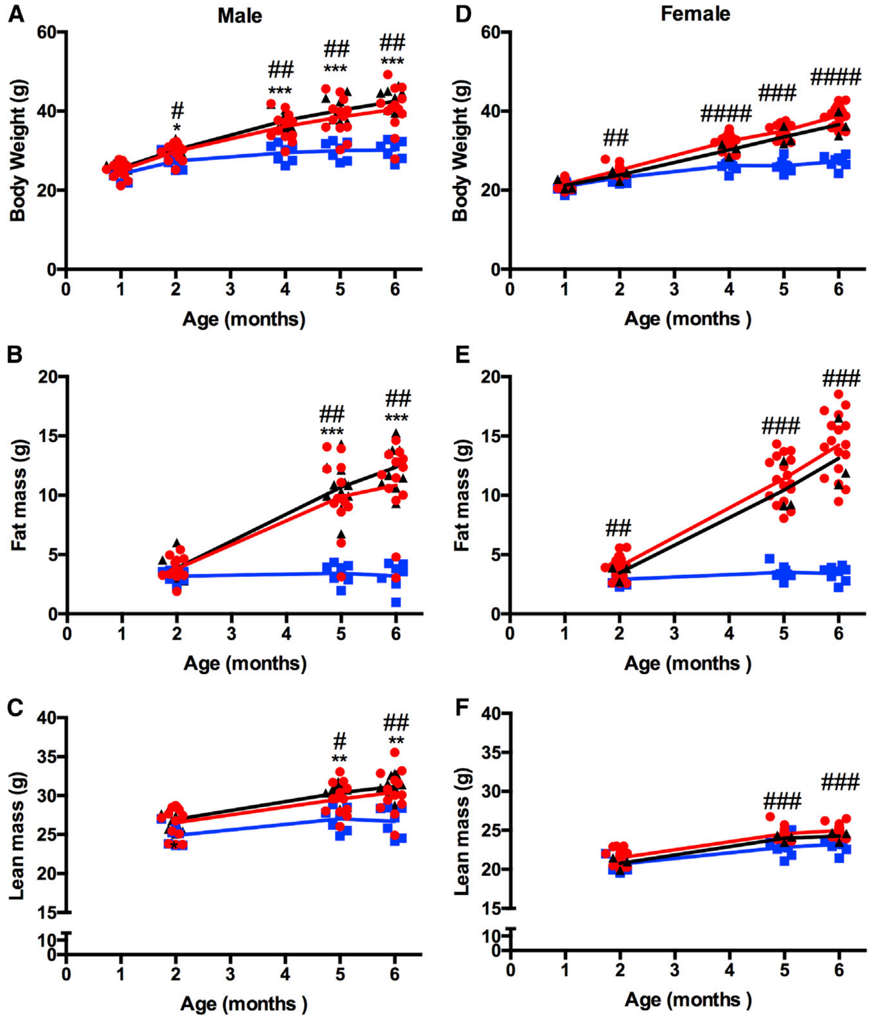

Figure 2. *Wars2$^{V117L/V117L}$* Mice Have Reduced Body Weight due to Reduced Adiposity

Male and female cohort 1 mice: (A and D) body weight, (B and E) fat mass, and (C and F) lean mass, respectively. *Wars2$^{V117L/V117L}$*, *Wars2$^{V117L/+}$*, and *Wars2$^{+/+}$* littermate numbers were 7 and 8, 13 and 17–21, and 11 and 3 male and female, respectively; mean ± SD. Areas under the curve (AUCs) were compared for males using an ordinary one-way ANOVA with Tukey's multiple comparison test and for females using a one-way ANOVA non-parametric Kruskal-Wallis test and Dunn's multiple comparison test. For AUC for *Wars2$^{+/+}$* and *Wars2$^{V117L/V117L}$*, *Wars2$^{+/V117L}$* and *Wars2$^{V117L/V117L}$*, and *Wars2$^{+/+}$* and *Wars2$^{V117L/+}$*, male body weight was $p < 0.0001$, $p = 0.0002$, and $p > 0.5057$; fat mass was $p < 0.0001$, $p < 0.0001$, and $p = 0.6505$; and lean mass was $p = 0.0063$, $p = 0.0008$, and $p = 0.5314$. Female body weight for *Wars2$^{+/V117L}$* and *Wars2$^{V117L/V117L}$* was $p < 0.0001$, fat mass was $p = 0.0001$, and lean mass was $p = 0.0055$ (wild-type [WT] comparisons not shown, because n = 3). Significance at specific time points was calculated with a one-way ANOVA non-parametric Kruskal-Wallis test and Dunn's multiple comparison test. Significance between *Wars2$^{+/+}$* and *Wars2$^{V117L/V117L}$* and between *Wars2$^{+/V117L}$* and *Wars2$^{V117L/V117L}$* is shown as * and #$p < 0.05$, ** and ##$p < 0.01$, and *** and ###$p < 0.001$, respectively. *Wars2$^{V117L/V117L}$* mice are blue squares, *Wars2$^{V117L/+}$* mice are red circles, and *Wars2$^{+/+}$* mice are black triangles. See also Figure S4.

predict that it was deleterious. However, the mutated nucleotide is the first coding nucleotide of exon 3, and the NetGene2 splice site prediction program indicated that substitution of G to T at the first nucleotide of the third exon of *Wars2* would affect the efficiency of exon 3 splicing (Figure 4A) (Hebsgaard et al., 1996). We modeled the predicted consequence of exon 3 skipping and found that three α helices, required for substrate binding and release, are missing, which would likely lead to a loss of WARS2 function (Figure S1F). To test the prediction of exon skipping *in vivo*, RT-PCR analysis of cochlear RNA derived from wild-type, heterozygous mutant, and homozygous mutant mice was undertaken (Figure 4B). This showed the *Wars2^{V117L}* allele, c.349G > T, caused in-frame skipping of exon 3. However, the mutation does not abolish normal splicing, and some full-length transcript is still produced (Figure 4B). Although the full-length transcript was severely decreased in homozygotes, the small amount still produced would generate mitochondrial *Wars2* tryptophanyl-tRNA synthetase 2 protein (mtTrpRS) (with the p.V117L substitution) and explains why *Wars2$^{V117L/V117L}$* mutants were viable, unlike *Wars2$^{-/-}$* nulls (Table S1).

We then investigated changes in RNA splicing using three TaqMan probes targeting *Wars2* 2/3, 4/5, and 5/6 exon-exon boundaries in tissues (Figure 4C). Exon 4/5 and exon 5/6 probes, present in all transcripts, were significantly reduced in the heart, kidney, and BAT and were unchanged in the other tissues (Figure 4C). Exon 2/3 junctions, present only in full-length transcript (*Wars2^{FL}*), were significantly reduced in all *Wars2$^{V117L/V117L}$* tissues (Figure 4C). To determine the effect of these differences on WARS2 steady-state protein levels, tissues from *Wars2$^{V117L/V117L}$* mice were analyzed by immunoblotting. Consistent with the RNA results, WARS2 protein was significantly decreased in heart, liver, kidney, skeletal muscle, iWAT, and BAT of *Wars2$^{V117L/V117L}$* mice (Figure 4D; Figure S5A).

The *Wars2* c.349G>T Mutation Caused Tissue-Specific OXPHOS Deficiencies

To determine the functional effects of reduced WARS2 protein in the mitochondria, steady-state levels of mitochondrial oxidative phosphorylation (OXPHOS) components (complex I–CV) were quantified by immunoblotting in each tissue at 12 months of age (Figures 4E–4J; Figure S5A). Consistent with decreased WARS2 protein, steady-state CI (NADH:ubiquinone

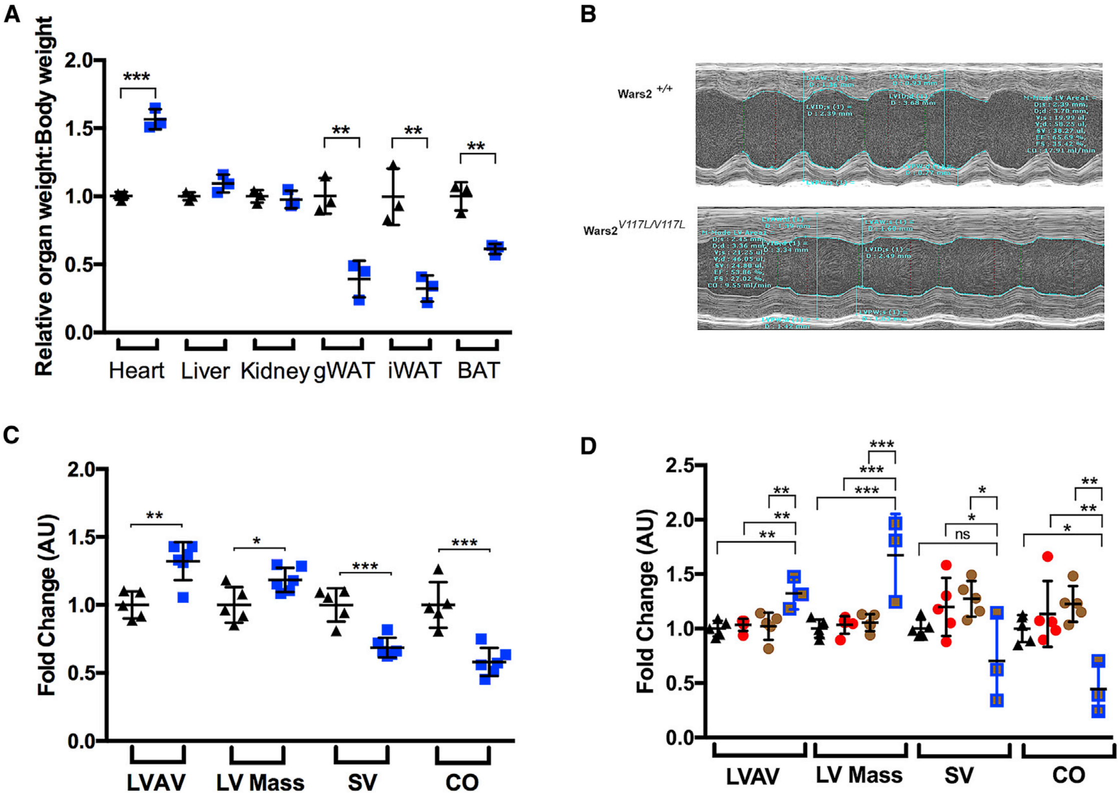

Figure 3. The *Wars2*^{V117L} Allele Causes Hypertrophic Cardiomyopathy

(A) Organ weight divided by body weight at 6 months of age in male mice. *Wars2*$^{V117L/V117L}$ and *Wars2*$^{+/+}$ animal numbers were 3 and 3, respectively; mean ± SD. Data were analyzed with multiple t tests by the Holm-Sidak method.

(B) Representative echocardiogram images of the left ventricle in male *Wars2*$^{V117L/V117L}$ and *Wars2*$^{+/+}$ mice at 6 months of age.

(C) Functional analysis of images for left ventricle anterior wall (LVAW) diameter, left ventricle (LV) mass, stroke volume (SV), and cardiac output (CO). *Wars2*$^{V117L/V117L}$ and *Wars2*$^{+/+}$ male mice animal numbers were 6 and 5, respectively; mean ± SD. Data were analyzed using an ordinary one-way ANOVA with Tukey's post hoc test to correct for multiple comparisons.

(D) Echocardiogram analysis in *Wars2*$^{V117L/-}$ male mice at 6 months of age. *Wars2*$^{V117L/-}$, *Wars2*$^{V117L/+}$, *Wars2*$^{+/-}$ and *Wars2*$^{+/+}$ animal numbers were 3, 5, 5, and 5, respectively; mean ± SD. Data were analyzed using an ordinary one-way ANOVA with Tukey's post hoc test to correct for multiple comparisons.

*$p < 0.05$, **$p < 0.01$, ***$p < 0.001$. *Wars2*$^{V117L/V117L}$ mice are blue squares, *Wars2*$^{+/+}$ mice are black triangles, *Wars2*$^{V117L/+}$ mice are red filled circles, compound heterozygote *Wars2*$^{V117L/-}$ mice are brown filled blue squares, and *Wars2*$^{+/-}$ mice are brown circles.

oxidoreductase subunit B8 [NDUFB8]) and CIV (mitochondrially encoded cytochrome *c* oxidase I [MTCO1]) protein levels were significantly lower in *Wars2*$^{V117L/V117L}$ heart (Figure 4E; Figure S5A), liver (Figure 4F; Figure S5A), and BAT (Figure 4G; Figure S5A). Furthermore, respiratory complex activity measurements in heart showed decreased CI and CIV activities (Figures S5B and S5C). In addition, CIII (ubiquinol:cytochrome *c* reductase core protein 2 [UQCRC2]) steady-state protein levels were decreased in *Wars2*$^{V117L/V117L}$ BAT, showing more profound inhibition of mitochondrial translation in BAT compared to heart or liver (Figure 4G; Figure S5A). By comparison, no differences in CII (succinate:ubiquinone oxidoreductase complex flavoprotein subunit A [SDHA]), CIII, and CV (ATP synthase F1 subunit alpha [ATP5A]) subunit protein levels were observed in *Wars2*$^{V117L/V117L}$ heart or liver; CV subunit levels were mildly increased in *Wars2*$^{V117L/V117L}$ BAT relative to wild-type controls (Figure S5A).

Despite a severe loss of WARS2 protein, OXPHOS subunit steady-state protein levels remained largely unchanged in the *Wars2*$^{V117L/V117L}$ kidney, with only a significant reduction in CV and a trend to mildly reduce CI observed at 12 months of age (Figure 4H; Figure S5A). Furthermore, immunoblot analysis showed a significant increase in CI and a trend toward increased CIII (unadjusted $p = 0.029$) steady-state OXPHOS protein levels in *Wars2*$^{V117L/V117L}$ iWAT, despite decreased WARS2 protein (Figure 4I; Figure S5A). *Wars2*$^{V117L/V117L}$ skeletal muscle showed no consistent respiratory chain deficiencies (Figure 4J; Figure S5A), although CIII steady-state protein levels appeared to be increased in skeletal muscle. This was confirmed in skeletal muscle by measurement of respiratory chain complex activities, which also showed a significant increase in CIII activity (Figure S5C).

Given the observation of brain pathology in patients, we also determined steady-state WARS2 and OXPHOS components in brains of mice 3–5 months of age and observed clear reduction of WARS2 protein and complex I subunit deficiency and a trend toward CIV deficiency (Figures S5D–S5F), indicating that the brain is not spared.

OPEN
ACCESS
CellPress

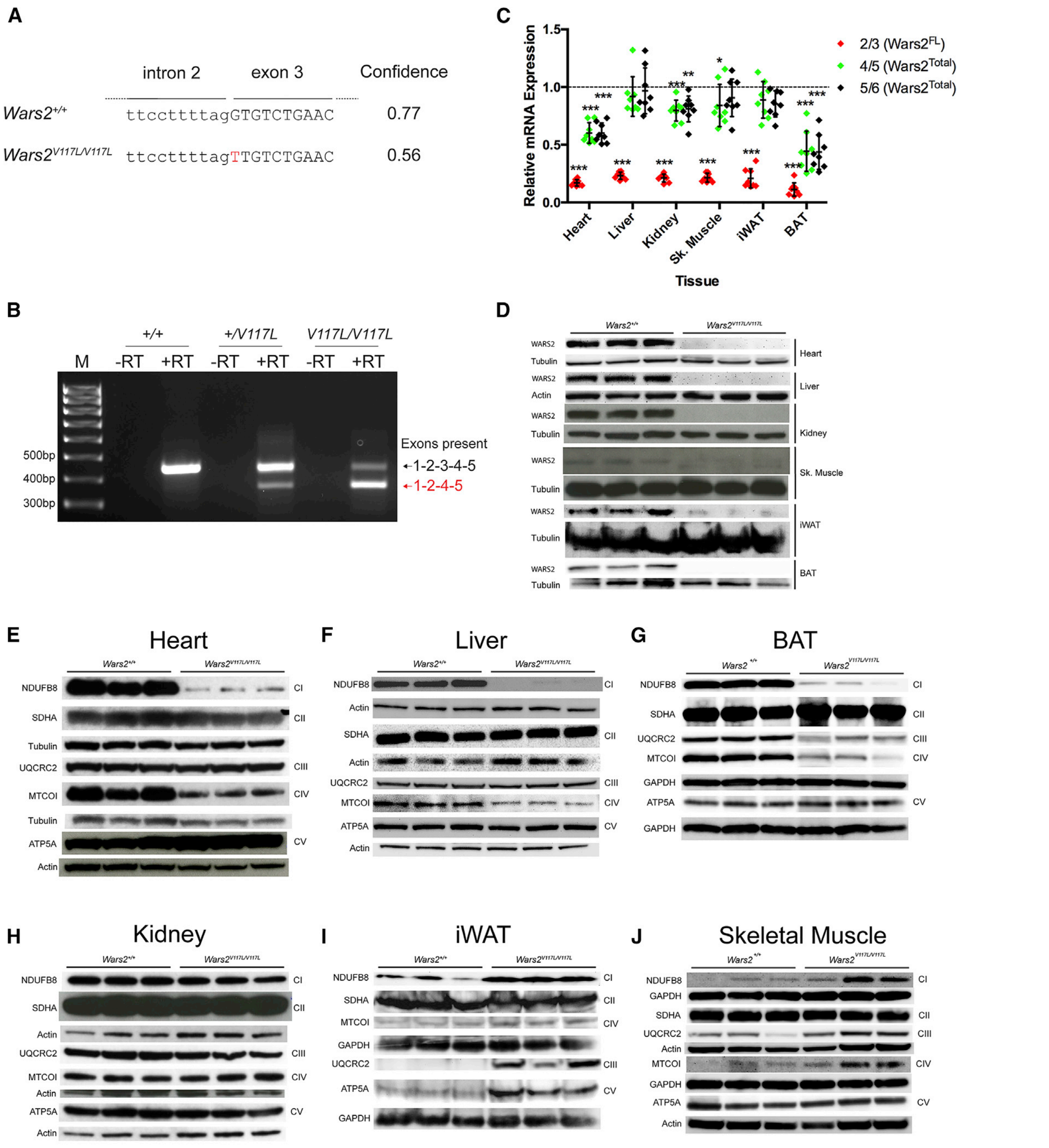

Antibody probe key:
Complex I (CI) - NADH:ubiquinone oxidoreductase Subunit B8 (NDUFB8)
Complex II (CII) - Succinate Dehydrogenase Complex Flavoprotein Subunit A (SDHA);
Complex III (CIII) - Ubiquinol-cytochrome c oxidoreductase Core Protein II (UQCRC2)
Complex IV (CIV) - mitochondrially-encoded cytochrome c oxidase I (MTC01)
Complex V (CV) - ATP Synthase, H+ Transporting, Mitochondrial F1 Complex, Alpha Subunit 1, Cardiac Muscle (ATP5A)

Figure 4. *Wars2*^{V117L} Allele Increases Exon Skipping, Causing Tissue-Specific *WARS2* and Mitochondrial Respiratory Chain Deficiencies
(A) *Wars2* c.349G > T NetGene2 splice site prediction.
(B) RT-PCR of RNA extracted from cochleae of *Wars2*$^{+/+}$, *Wars2*$^{V117L/+}$, and *Wars2*$^{V117L/V117L}$ mice using oligonucleotide primer pairs designed to exons 1 and 5. Products were sequenced and contain the exons indicated.

(legend continued on next page)

Wars2$^{V117L/V117L}$ Mice Show Browning of WAT and Dysfunctional BAT Pathology

To investigate the functional consequences of the contrasting differences in iWAT and BAT for CI, CIII, and CIV subunit steady-state levels, we carried out histological analysis at 12 months of age in males. iWAT showed qualitatively higher multi-locular lipid droplet formation indicative of browning, although this was also observed to a lesser extent in wild-type mice. Visceral gWAT appeared relatively normal (Figures 5A and 5B). Similar patterns were seen in females at 3 months of age (data not shown). Gene expression analysis showed significant upregulation of the key browning markers uncoupling protein 1 (*Ucp1*), iodothyronine deiodinase 2 (*Dio2*), and cell death-inducing DNA fragmentation factor subunit alpha (DFFA)-like effector a (*Cidea*) (Figure 5D) and immunoblot analysis showed increased UCP1 protein levels (Figure 5E) in *Wars2*$^{V117L/V117L}$ iWAT, showing activation of browning pathways. Furthermore, nuclear-encoded mitochondrial respiratory chain cytochrome *c* oxidase subunit 7B (*Cox7b*) and cytochrome *c* oxidase subunit 8A (*Cox8a*) mRNA were significantly increased in *Wars2*$^{V117L/V117L}$ iWAT (Figure 5D), showing transcriptional upregulation, consistent with the increased respiratory chain subunit protein levels shown earlier (Figure 4I).

Conversely, in BAT, males at 12 months (Figure 5C) and females at 3 months (data not shown) showed strikingly increased unilocular lipid droplet formation, indicative of inhibition of lipolysis and β-oxidation, and reduced BAT thermogenic function. In keeping with these observations, gene expression analysis showed downregulation of browning markers *Ucp1*, *Dio2*, and *Cidea*; and peroxisome proliferator-activated receptor gamma (*Pparγ*) (Figure 5F) and immunoblot analysis showed reduced UCP1 protein (Figure 5G) in males at 12 months, consistent with BAT dysfunction. Nuclear-encoded mitochondrial respiratory chain subunit *Cox7b* and *Cox8a* mRNA expression levels were also decreased (Figure 5F), showing transcriptional downregulation, consistent with the respiratory chain dysfunction shown earlier (Figure 4G).

Given the abnormal BAT pathology and tissue-specific respiratory chain dysfunction observed, we carried out indirect calorimetry using a comprehensive laboratory animal monitoring system (CLAMS) at 4 months of age at 22°C (home cage temperature well below ∼28°C thermoneutrality). Energy expenditure (EE) was significantly reduced in female *Wars2*$^{V117L/V117L}$ mice (Figure 5H), consistent with the observed abnormal BAT pathology and tissue-specific respiratory chain dysfunction.

Upregulation of Mitochondrial Biogenesis Ameliorated Mitochondrial Respiratory Chain Dysfunction in *Wars2*$^{V117L/V117L}$ MEFs, Skeletal Muscle, and iWAT

We further examined the effects of *Wars2*-V117L on mitochondrial function in *Wars2*$^{V117L/V117L}$ mouse embryonic fibroblasts (MEFs), which were cultured and assayed using microscale oxygraphy (Figures 6A and 6B). Unexpectedly, *Wars2*$^{V117L/V117L}$ MEFs showed significantly increased basal respiration and ATP production compared to wild-type MEFs (Figures 6A and 6C), indicative of increased mitochondrial respiratory chain function. There was no difference in glycolysis as measured by extracellular acidification rate (ECAR) (Figure 6B). We hypothesized that this could be due to increased mitochondrial mass and upregulation of mitochondrial biogenesis. To directly measure mitochondrial mass, MEFs were stained with MitoTracker green, which localizes to mitochondria in live cells independent of mitochondrial membrane potential, and the average fluorescence per cell (30,000 cells per sample) was quantified by fluorescence-activated cell sorting (Figure 6D). We found that the average fluorescence intensity increased 40% in *Wars2*$^{V117L/V117L}$ MEFs relative to *Wars2*$^{+/+}$ controls showing increased mitochondrial mass (Figure 6D). Consistent with this, the master regulator of mitochondrial biogenesis, peroxisome proliferator-activated receptor gamma coactivator 1-alpha (*Pgc1α*), was significantly upregulated in *Wars2*$^{V117L/V117L}$ MEFs (Figure 6E).

MEFs are derived from the mesenchyme stem cell lineage. We hypothesized that mature tissues composed primarily of cells derived from the mesenchymal stem cell lineage, such as myocytes and adipocytes (skeletal muscle and iWAT, respectively), upregulate *Pgc1α* and mitochondrial biogenesis to prevent respiratory chain dysfunction in *Wars2*$^{V117L/V117L}$ mice. Gene expression analysis showed *Pgc1α* increased, on average, 3.3- and 4.3-fold in *Wars2*$^{V117L/V117L}$ skeletal muscle and iWAT, respectively, at 12 months of age (Figure 6F), showing transcriptional upregulation of mitochondrial biogenesis, consistent with the increased respiratory chain subunits observed previously (Figures 4I and 4J). No significant differences in *Pgc1α* expression were observed in other tissues (Figure 6F).

These data show *Pgc1α* is upregulated in *Wars2*$^{V117L/V117L}$ tissues displaying increased respiratory chain subunit levels, such as iWAT, indicating upregulation of mitochondrial biogenesis prevented respiratory chain dysfunction. Furthermore, *Pgc1α* is not upregulated in *Wars2*$^{V117L/V117L}$ heart or BAT, in which respiratory chain dysfunction and disease pathology were observed, or in kidney, in which respiratory chain subunit levels are comparable with controls. Transcription factor A, mitochondrial (*Tfam*), required for transcription and associated with mtDNA copy number, was reduced in heart and BAT (Figure 6G).

(C) To quantify *Wars2* mRNA missplicing *in vivo*, RNA was extracted from tissues from mice at 12 months of age and qRT-PCR was performed using 3 TaqMan probes targeted to alternate *Wars2* exon-exon boundaries and expressed relative to *Wars2*$^{+/+}$: boundaries 2/3 (Wars2^{FL}), 4/5 (Wars2^{Total}), and 5/6 (*Wars2*^{Total}). *Wars2*$^{V117L/V117L}$ and *Wars2*$^{+/+}$ littermate numbers were 8 and 8 (5 male and 3 female), respectively; mean ± SEM. Data were analyzed using a Mann-Whitney two-tailed t test. Significance differences between *Wars2*$^{V117L/V117L}$ and *Wars2*$^{+/+}$ samples are shown as *p < 0.05, **p < 0.01, and ***p < 0.001.

(D–J) Immunoblot analysis of (D) WARS2 protein levels in multiple tissues and (E–J) mitochondrial respiratory chain subunit protein levels in (E) heart, (F) liver, (G) brown adipose tissue (BAT), (H) kidney, (I) inguinal white adipose tissue (iWAT), and (J) skeletal muscle from female mice at 12 months of age. *Wars2*$^{V117L/V117L}$ and *Wars2*$^{+/+}$ littermate numbers were 3 and 3, respectively.

See also Figure S5.

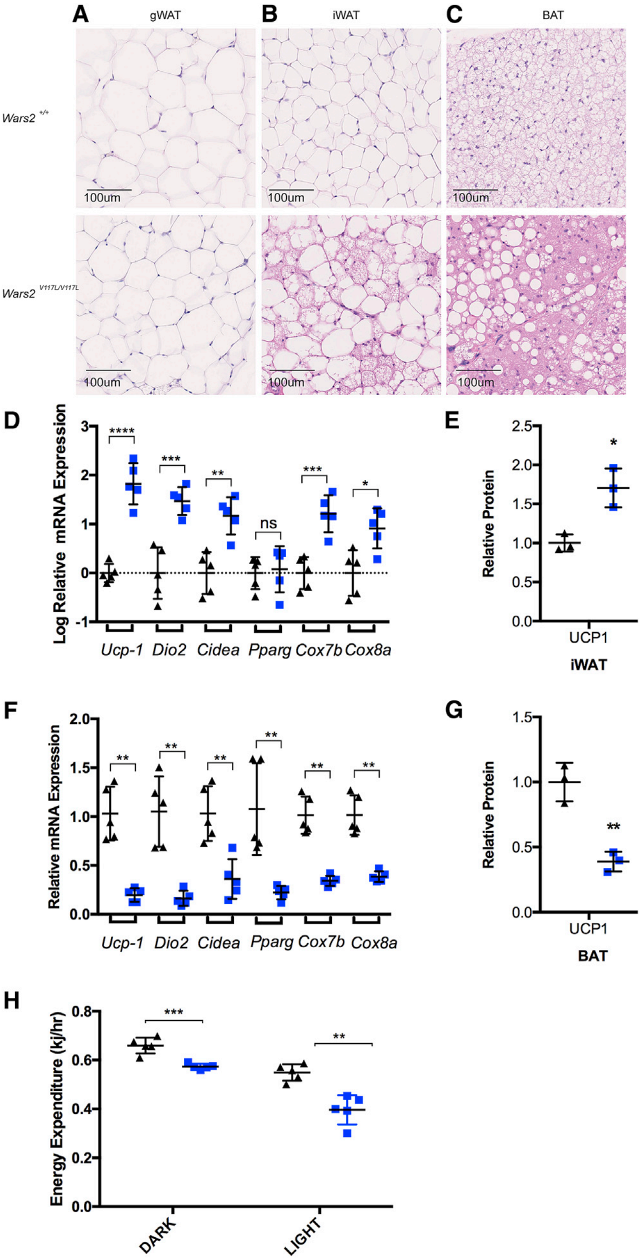

Figure 5. *Wars2*$^{V117L/V117L}$ Mice Show Browning of WAT and Dysfunctional BAT

(A–C) Representative images of H&E-stained sections of adipose tissue depots at 12 months of age from male mice. (A) Gonadal WAT (gWAT), (B) iWAT, and (C) BAT. Scale bar, 100 μm. *Wars2*$^{V117L/V117L}$ and *Wars2*$^{+/+}$ animal numbers were 3 and 3, respectively.

(D) Relative mRNA expression analysis of browning markers in iWAT from male mice at 12 months of age. *Wars2*$^{V117L/V117L}$ and *Wars2*$^{+/+}$ animal numbers were 5 and 5, respectively; mean ± SD. Data were log transformed and analyzed using an unpaired two-tailed t test with equal SD. *p < 0.05, **p < 0.01, ***p < 0.001.

(E) Relative UCP1 protein levels in iWAT from female mice at 12 months of age. *Wars2*$^{V117L/V117L}$ and *Wars2*$^{+/+}$ animal numbers were 3 and 3, respectively; mean ± SD. Data were analyzed with an unpaired t test. *p < 0.05.

(F) Relative mRNA expression analysis of browning markers in BAT from male mice at 12 months of age. *Wars2*$^{V117L/V117L}$ and *Wars2*$^{+/+}$ animal numbers were 8 and 8, respectively; mean ± SD. Data were analyzed with a Mann-Whitney two-tailed t test. **p < 0.01.

(G) Relative UCP1 protein levels in BAT from female mice at 12 months of age. *Wars2*$^{V117L/V117L}$ and *Wars2*$^{+/+}$ animal numbers were 3 and 3, respectively; mean ± SD. Data were analyzed with an unpaired t test. **p < 0.01.

(H) Energy expenditure (EE) normalized to lean mass by multiple linear regression (analysis of covariance [ANCOVA]) measured in female mice at 4 months of age. *Wars2*$^{V117L/V117L}$ and *Wars2*$^{+/+}$ animal numbers were 5 and 5, respectively; mean ± SD analyzed with an unpaired two-tailed t test. **p < 0.01, ***p < 0.001.

Wars2$^{V117L/V117L}$ mice are blue squares, and *Wars2*$^{+/+}$ mice are black triangles.

In addition, peroxisome proliferator-activated receptor alpha (*Pparα*) expression was increased in skeletal muscle and iWAT of *Wars2*$^{V117L/V117L}$ mice, consistent with increased *Pgc1α* expression (Figure 6H). Conversely, *Pparα* was significantly decreased in the heart and BAT of *Wars2*$^{V117L/V117L}$ mice and was unchanged in liver and kidney (Figure 6H). Overall, these data show that the tissue-specific respiratory chain dysfunction observed is partly because of the tissue-specific capacity for upregulation of *Pgc1α* and compensatory mitochondrial biogenesis.

Heart-Specific Activation of the ISR Caused Increased Plasma FGF21 and Systemic Changes in Metabolism

Fasted plasma FGF21 protein levels showed a trend toward elevation in male *Wars2*$^{V117L/V117L}$ mice relative to controls at 12 months (Figure 7A) and similarly, but reaching significance, at 4 months of age in males only (Figures S6A and S6B). Plasma clinical chemistry analysis showed unchanged plasma free fatty acid levels and plasma glucose levels (Figures S6C and S6D), trends for reduced plasma triglycerides (Figure S6E), and markedly increased plasma ketone bodies (β-hydroxybutyrate) (Figure S6F) in *Wars2*$^{V117L/V117L}$ mice. Furthermore, intraperitoneal glucose tolerance tests (IPGTTs) demonstrated increased glucose tolerance relative to wild-type controls (Figure S6G). FGF21 has previously been shown to reduce body weight by stimulating WAT lipolysis, induce temperature-dependent browning of WAT (Fisher et al., 2012), increase glucose tolerance by increasing insulin-independent glucose uptake in WAT and skeletal muscle (Kharitonenkov et al., 2005; Mashili et al., 2011), and increase hepatic ketogenesis (Inagaki et al., 2007). Thus, our findings, together with the reduced adiposity phenotype (Figure 2) and increased WAT browning observed previously (Figures 5A and 5B), align with the known effects of FGF21 on systemic metabolism and implicate FGF21 as the cause of metabolic phenotypes observed in *Wars2*$^{V117L/V117L}$ mice.

FGF21 has previously been shown to be transcriptionally regulated via independent pathways governed by ATF4 and PPARα (Inagaki et al., 2007; Kim et al., 2013). To determine the mechanism of increased plasma FGF21, gene expression analysis was performed in *Wars2*$^{V117L/V117L}$ mice at 12 months of age. *Fgf21* expression was significantly increased in heart, skeletal muscle, and iWAT (Figure 7B). No difference was observed in other tissues (Figure 7B). A significant reduction in *Atf4* expression was observed in BAT, but not in other tissues (Figure 7C). However, regulation of ATF4 at the protein level is key in *Fgf21* regulation. In other tissues, such as skeletal muscle and iWAT, an alternate

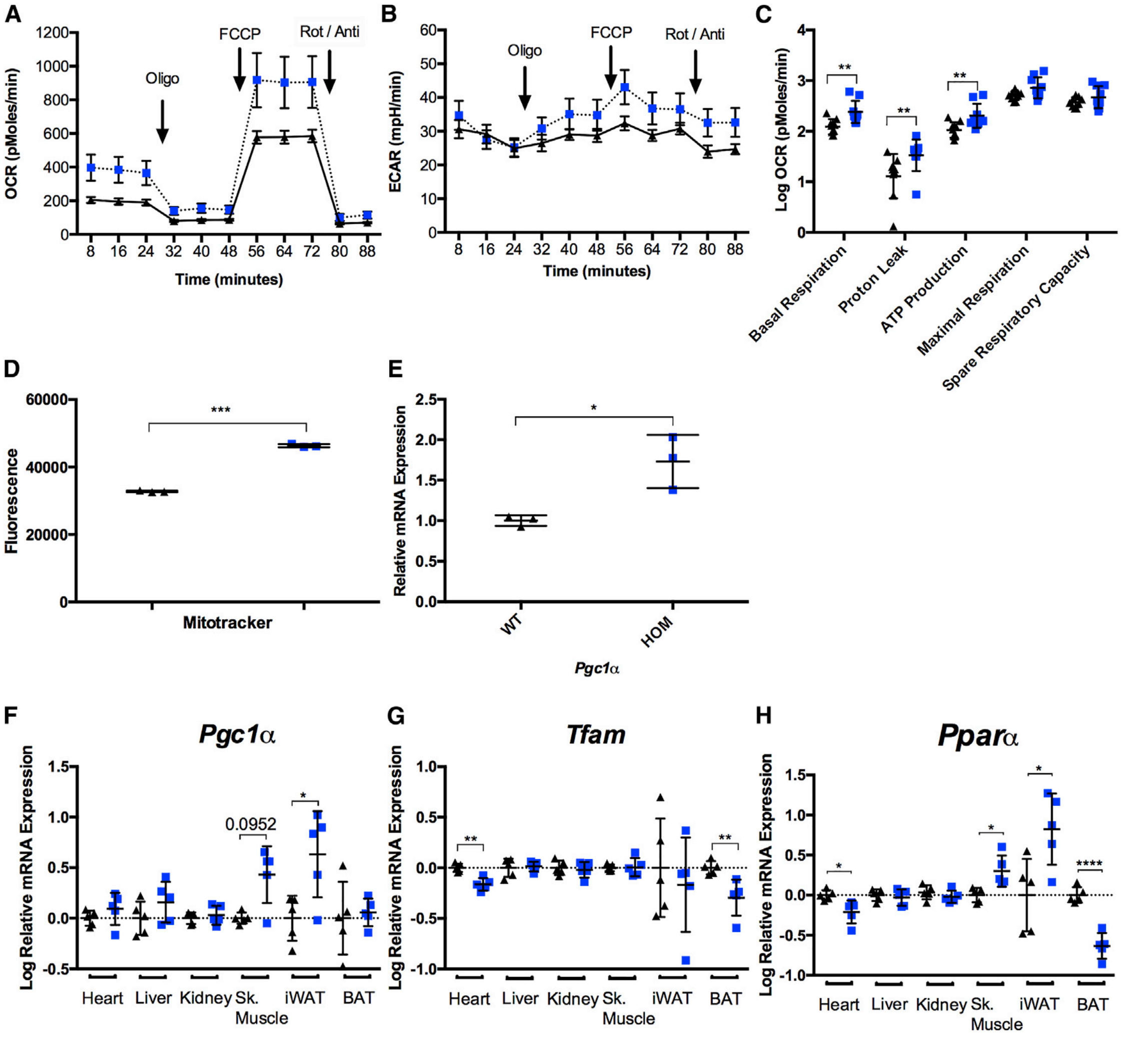

Figure 6. Upregulation of Mitochondrial Biogenesis Prevents Mitochondrial Respiratory Chain Dysfunction in *Wars2*$^{V117L/V117L}$ MEFs, Skeletal Muscle, and iWAT

(A and B) Oxygen consumption rate (OCR) (A) and extracellular acidification rate (ECAR) (B) were measured in cultured primary mouse embryonic fibroblasts harvested from *Wars2*$^{V117L/V117L}$ and *Wars2*$^{+/+}$ embryos using a Seahorse XF24 analyzer. OCR and ECAR measurements were taken at baseline and following oligomycin (Oligo), carbonyl cyanide 4-(trifluoromethoxy)phenylhydrazone (FCCP), and rotenone and antimycin (Rot/Anti) treatment. OCR and ECAR measurements were normalized to live cell number.

(C) Relative oxygen consumption rates of basal respiration, proton leak, ATP production, maximal respiration, and spare respiratory capacity in *Wars2*$^{+/+}$ and *Wars2*$^{V117L/V117L}$ MEF cultures. There were 9 replicates of each genotype; mean ± SD. Data were log transformed and analyzed using an unpaired two-tailed t test or a Mann-Whitney t test.

(D) MEFs were stained with MitoTracker green, and fluorescence in 30,000 cells per sample was quantified by fluorescence-activated cell sorting (FACS). There were 3 replicates of each genotype; mean fluorescence ± SD. Data were analyzed using an unpaired t test.

(E) Relative mRNA expression analysis of *Pgc1α* in *Wars2*$^{+/+}$ and *Wars2*$^{V117L/V117L}$ MEF cultures. There were 3 replicates of each genotype; mean fluorescence ± SD. Data were analyzed using an unpaired t test.

(F–H) Relative mRNA expression analysis of (F) *Pgc1α*, (G) *Tfam*, and (H) *Pparα* in tissues harvested from *Wars2*$^{V117L/V117L}$ and *Wars2*$^{+/+}$ male mice at 12 months of age. *Wars2*$^{V117L/V117L}$ and *Wars2*$^{+/+}$ animal numbers were 5 and 5, respectively; mean ± SD. Data are shown as log transformed and analyzed using an unpaired two-tailed t test or a Mann-Whitney t test (skeletal muscle and iWAT for *Pgc1α*).

*p < 0.05, **p < 0.01, ***p < 0.001, ****p < 0.0001. *Wars2*$^{V117L/V117L}$ mice are blue squares, and *Wars2*$^{+/+}$ mice are black triangles.

mechanism governed by *Pparα* could contribute to the increased plasma FGF21 observed in *Wars2*$^{V117L/V117L}$ mice (Figure 6H).

Upon various cellular stresses, the ISR is activated by phosphorylation of eukaryotic translation initiation factor 2A (eIF2α), resulting in reduced cytoplasmic 5′ cap-dependent protein synthesis and preferential translation of mRNAs that contain upstream open reading frames in their 5′ UTR, such as ATF4 (Lu et al., 2004). ATF4 has been shown to transcriptionally regulate stress response genes, including *Atf5*, DNA damage-inducible

OPEN ACCESS
CellPress

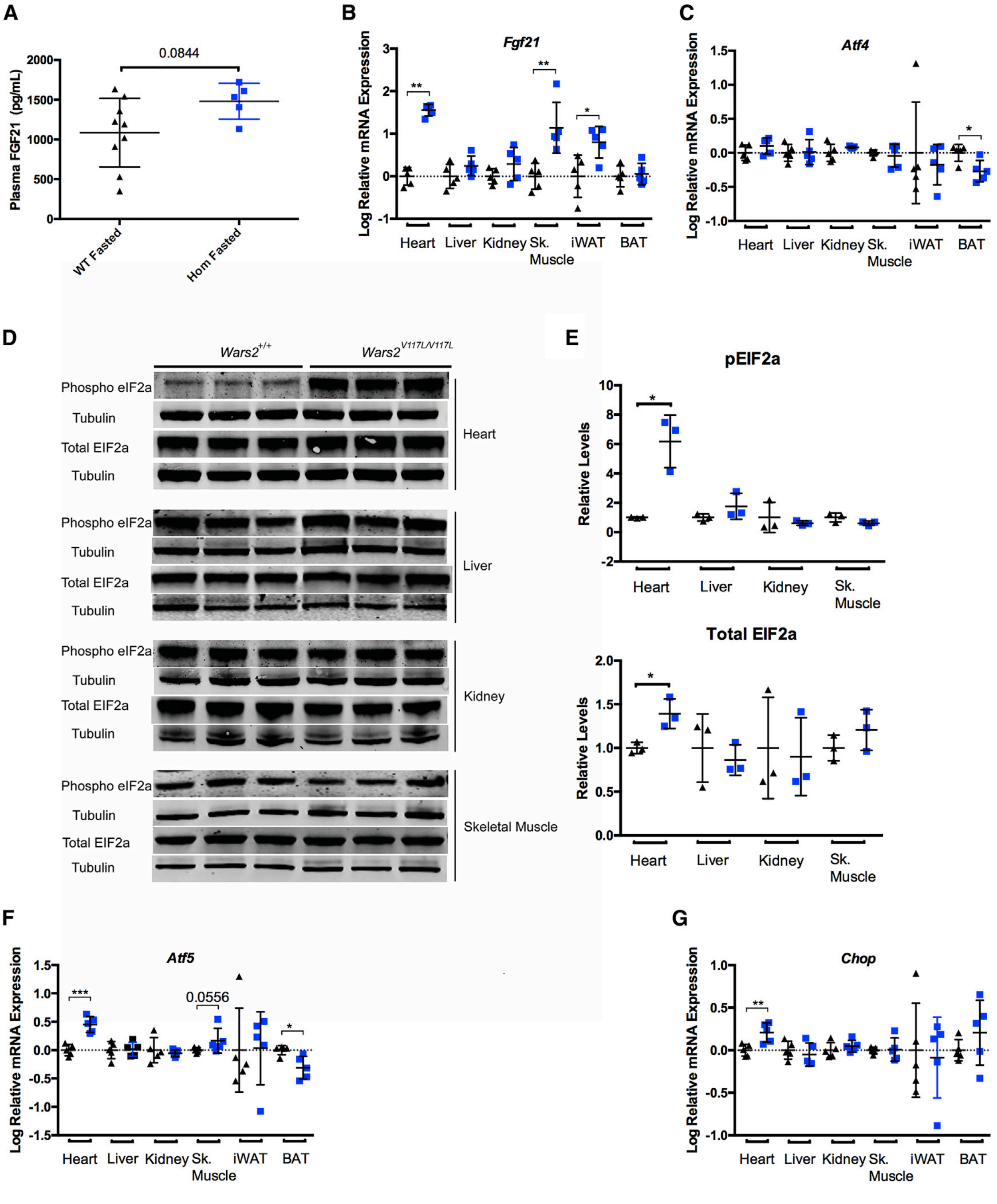

Figure 7. Heart-Specific Activation of the Integrated Stress Response Causes Increased Plasma FGF21 and Systemic Changes in Metabolism

(A) Relative plasma FGF21 protein levels in male mice at 12 months of age. *Wars2*$^{V117L/V117L}$ and *Wars2*$^{+/+}$ animal numbers were 5 and 9, respectively; mean ± SD. Unpaired t test.

(B and C) Relative mRNA expression levels of (B) *Fgf21* and (C) *Atf4* in tissues from male mice at 12 months of age. *Wars2*$^{V117L/V117L}$ and *Wars2*$^{+/+}$ animal numbers were 5 and 5, respectively; mean ± SD. Data were log transformed and analyzed using t tests or a Mann-Whitney test (*Fgf21* in heart and *Atf4* in skeletal muscle and iWAT). *Fgf21* RNA expression is very low in kidney and in wild-type skeletal muscle: mean $C_T > 33$.

(D and E) Immunoblot analysis (D) and quantification (E) of p-eIF2α and total EIF2α protein levels in heart, liver, kidney, and skeletal muscle from female mice at 12 months of age. *Wars2*$^{V117L/V117L}$ and *Wars2*$^{+/+}$ animal numbers were 3 and 3, respectively; bands were normalized to tubulin and expressed relative to wild-type as the mean ± SD. Significance was determined using an unpaired t test with Welch's correction.

(legend continued on next page)

transcript 3 (C/EBP homologous protein [*Chop*]), and *Fgf21* (De Sousa-Coelho et al., 2012). We hypothesized that the *Wars2*-V117L allele caused cardiac-specific activation of the ISR pathway, leading to increased *Fgf21* expression and activation of stress response pathways. To assess activation of the ISR, we performed immunoblot analysis of phosphorylated eukaryotic translation initiation factor 2A (p-eIF2α)/eIF2α steady-state protein levels in *Wars2*$^{V117L/V117L}$ heart, liver, kidney, and skeletal muscle at 12 months of age (Figures 7D and 7E). We found that p-eIF2α levels are significantly increased in heart (average of 6.1-fold) compared to controls (Figure 7E). Conversely, no significant differences in p-eIF2α levels were observed in kidney, liver, or skeletal muscle (Figure 7E). Furthermore, ISR pathway genes such as *Atf5* and *Chop* were significantly increased at the mRNA level in the heart of *Wars2*$^{V117L/V117L}$ mice (Figures 7F and 7G). However, no significant differences in *Atf5* or *Chop* were observed in liver, kidney, skeletal muscle, or iWAT (Figures 7F and 7G). Altogether, these data show robust tissue-specific activation of the ISR in the heart of *Wars2*$^{V117L/V117L}$ mice.

Progressive Activation of the ISR Is Coincident with CI Deficiency and Independent of Disrupted Mitochondrial Proteostasis in the Heart of *Wars2*$^{V117L/V117L}$ Mice

Disrupted mitochondrial proteostasis, rather than respiratory chain dysfunction, was the primary stress caused by inhibition of mitochondrial translation, leading to ATF4-dependent FGF21 expression and systemic changes in metabolism in the heart of *Dars2*-KO^{Ckmm} mice (Dogan et al., 2014). To characterize activation of the ISR in the heart, we performed time course immunoblot analysis of the ISR marker p-eIF2α, CI and CIV OXPHOS subunits, and mitochondrial proteostasis markers caseinolytic mitochondrial matrix peptidase proteolytic subunit (CLPP), Lon peptidase 1, mitochondrial (LONP1), heat shock protein family D (Hsp60) member 1 (HSP60), and mitochondrial heat shock protein family A (Hsp70) member 9 (mtHSP70) at 1, 3, and 12 months of age (Figures S7A–S7C). At 1 month, no differences in p-eIF2α, CI (NDUFB8), or CIV (COXI) steady-state protein levels were observed (Figure S7A). At 3 and 12 months of age, p-eIF2α levels were increased (Figure 7E; Figures S7B and S7C). At 3 and 12 months of age, CI and CIV levels were decreased (Figures S7B and S7C). Altogether, these data show that activation of the ISR and respiratory chain dysfunction occurred after 1 month of age in the heart of *Wars2*$^{V117L/V117L}$ mice and that activation of the ISR was progressive, with age from 3 to 12 months, and coincident with progressive CI deficiency. CIV deficiencies were comparable between 3 and 12 months of age, showing no progressive further deficiency.

Finally, we found no significant differences in the steady-state protein levels of LONP1, CLPP, HSP60, or mtHSP70 at any time point measured in the heart of *Wars2*$^{V117L/V117L}$ mice with the exception of a mild reduction in LONP1 protein at only 1 month of age (Figures S7A–S7C).

DISCUSSION

Sensorineural hearing loss is a common feature of human mitochondrial disease, and mutations in *LARS2*, *HARS2*, and *NARS2* have been shown to cause sensorineural hearing loss (Pierce et al., 2011, 2013; Simon et al., 2015). Reduced body mass is also associated with mitochondrial diseases (Wolny et al., 2009). Furthermore, mutations in genes encoding critical proteins of the mitochondrial translation system, including *MTO1*, *GTBP3*, and *ELAC2* (Baruffini et al., 2013; Haack et al., 2013; Kopajtich et al., 2014) and several mt-aaRS mutations in *AARS2*, *PARS2*, *SARS2*, and *YARS2*, have been shown to cause hypertrophic cardiomyopathy (Belostotsky et al., 2011; Götz et al., 2011; Riley et al., 2010; Rivera et al., 2013; Shahni et al., 2013; Sofou et al., 2015). Individuals with compound heterozygous *WARS2* mutations showing neurological problems have been reported (Burke et al., 2018; Musante et al., 2017; Theisen et al., 2017; Vantroys et al., 2018; Wortmann et al., 2017). We demonstrate that hypomorphic *Wars2* alleles, *Wars2*$^{V117L/-}$ and *Wars2*$^{V117L/V117L}$, which do not have direct genocopies in humans, cause sensorineural hearing loss, reduced adiposity, and hypertrophic cardiomyopathy in mice. However, we have not observed gross neurological effects during welfare observations or morphological differences using light microscopy of brain sections. This may reflect the severity of the alleles described so far in patients, in comparison with this mouse hypomorphic allele, and possible species differences.

Genetic mapping in the rat for coronary flow and capillary density traits in the heart identified a causal missense variant in *Wars2* that reduced WARS2 activity by $\sim$40% (Wang et al., 2016). It was also shown that the mutation reduced endothelial cell proliferation and activated pro-apoptotic pathways, as well as impairing BAT function (Pravenec et al., 2017; Wang et al., 2016). Finally, Wang et al. (2016) demonstrated that Wars2 is a critical pro-angiogenic factor in zebrafish. We have not carried out an analysis of coronary vasculature in the *Wars2*$^{V117L/V117L}$ model, although we observed BAT dysfunction.

As in our model, human hypomorphic mt-aaRS mutations cause tissue-specific pathology and respiratory chain dysfunction in humans, although the tissue-specific mechanisms remain unknown. However, increased respiratory chain subunits observed in iWAT of *Wars2*$^{V117L/V117L}$ were associated with upregulation of *Pgc1α* mRNA expression. Furthermore, we showed increased *Pgc1α* expression, mitochondrial mass, and function in *Wars2*$^{V117L/V117L}$ MEFs. We suggest that *Pgc1α* is upregulating mitochondria mass, preventing impaired respiratory chain function. In support of this, targeting *Pgc1α* to upregulate mitochondrial biogenesis, via therapeutic administration or genetic manipulation, can alleviate disease traits and increase mitochondrial respiratory capacity in human patient cell lines and mouse models (Bastin et al., 2008; Khan et al., 2014). Altogether, these data indicate that tissue-specific upregulation of

(F and G) Relative mRNA expression levels of (F) *Atf5* and (G) *Chop* in male *Wars2*$^{V117L/V117L}$ and *Wars2*$^{+/+}$ tissues harvested at 12 months of age. *Wars2*$^{V117L/V117L}$ and *Wars2*$^{+/+}$ animal numbers were 8 and 8, respectively; mean ± SD. Data were log transformed and analyzed using a t test or a Mann-Whitney test (*Atf5* and *Chop* in kidney and skeletal muscle).

*$p < 0.05$, **$p < 0.01$, ***$p < 0.001$. *Wars2*$^{V117L/V117L}$ mice are blue squares, and *Wars2*$^{+/+}$ mice are black triangles. See also Figures S6 and S7.

mitochondrial biogenesis explains the pattern of respiratory chain deficiencies observed in *Wars2*$^{V117L/V117L}$ mice. However, it remains unclear how some tissues in *Wars2*$^{V117L/V117L}$ mice upregulate *Pgc1α* and are protected while other tissues are not. We speculate that this is explained partly by exogenous FGF21, because studies have shown that FGF21 regulates browning of WAT in response to adaptive thermogenesis and that this effect is partly because of increased PGC1a protein levels (Fisher et al., 2012). In addition, FGF21, which potentially signals metabolic demands from stressed mitochondria to other tissues in the body, was shown to regulate mitochondrial mass in BAT of polymerase gamma mutator (POLG) mice following high fat diet (HFD) administration (Wall et al., 2015). Some effects of FGF21, such as in fat, could also be through an autocrine or paracrine mechanism, as reported in thermogenic recruitment of WAT (Fisher et al., 2012). FGF21, a biomarker of mitochondrial translation defects in human, likely has a beneficial role in tissues such as WAT by upregulating browning and mitochondrial biogenesis, providing some explanation for the tissue-specific respiratory chain deficiencies observed in *Wars2*$^{V117L/V117L}$ mice.

Common single-nucleotide polymorphisms, such as rs984222, with an effect allele frequency of 0.635, are associated with a 45% reduction in *WARS2* RNA expression in multiple tissues, including adipose (GTExPortal, http://www.gtexportal.org/home/). These single-nucleotide polymorphisms (SNPs) are associated with the waist-hip ratio in human genome-wide association studies, which are explained by changes in adipose tissue distribution (Heid et al., 2010). Our studies support the possibility that *WARS2* is one of the effector genes in this association locus (Pravenec et al., 2017).

Oxidative stress, mitochondrial unfolded protein response (UPR^{mt}), inhibition of mitochondrial translation, and respiratory chain dysfunction are linked to activation of the ISR (Baker et al., 2012; Kim et al., 2013; Michel et al., 2015; Rath et al., 2012). Dogan et al. (2014) showed that knocking out *Dars2* caused tissue-specific activation of the UPR^{mt}, leading to ATF4-dependent *Fgf21* expression in the heart of *Dars2*^{Ckmm} mice before respiratory chain dysfunction and concluding that mitochondrial proteostasis was the primary stress. We also demonstrated that activation of the ISR was a cardiac-specific response to inhibition of mitochondrial translation in *Wars2*$^{V117L/V117L}$ mice, resulting in increased *Fgf21* gene expression. However, in contrast with the Dogan et al. (2014) findings, we showed that activation of the ISR was independent of UPR^{mt} activation, was progressive with age, and was coincident with progressive CI respiratory chain deficiency. Several studies have demonstrated activation of the ISR upon progressive respiratory chain deficiency independent of activation of the UPR^{mt}. Inhibition of expression of the mitochondrial genome via mtDNA depletion or inhibition of mitochondrial translation through doxycycline treatment caused respiratory complex deficiencies and activation of the ISR independent of UPR^{mt} activation *in vitro* (Michel et al., 2015). Furthermore, activation of the ISR due to doxycycline treatment depended on the eIF2α kinase GCN2 (Michel et al., 2015). Activation of the ISR in the heart of *Wars2*$^{V117L/V117L}$ mice is thus due to progressive respiratory chain dysfunction, is independent of the UPR^{mt}, and may occur via GCN2-dependent phosphorylation of eIF2α.

We conclude that inhibition of mitochondrial translation can cause ISR activation via alternate mechanisms that depend upon the degree of mitochondrial translation inhibition. We speculate that complete inhibition of mitochondrial translation, e.g., via *Dars2*-KO, results in the accumulation of unassembled nuclear-encoded respiratory chain subunits, causing severe proteostatic stress and UPR^{mt}-dependent ISR activation. In contrast, partial inhibition of mitochondrial translation, e.g., *Wars2*$^{V117L/V117L}$ heart, causes activation of the ISR due to respiratory chain dysfunction and loss of mitochondrial membrane potential. The failed ability of the ISR to attenuate mitochondrial proteostatic stress likely explains the increased severity of the cardiac phenotype observed in *Dars2*-KO^{Ckmm} mice that cannot survive beyond 6 weeks of age compared to *Wars2*$^{V117L/V117L}$ mice.

In summary, we have generated a key mouse model for studying tissue-specific deficits in mitochondrial protein translation, linking phenotypes and mechanisms and offering the potential for therapeutic testing.

STAR★METHODS

Detailed methods are provided in the online version of this paper and include the following:

- KEY RESOURCES TABLE
- CONTACT FOR REAGENT AND RESOURCE SHARING
- EXPERIMENTAL MODEL AND SUBJECT DETAILS
  - Animal Models
  - Primary Cultures
- METHOD DETAILS
  - SNP Mapping and Whole Genome Sequencing
  - Genotyping
  - Auditory phenotyping
  - Body weight and composition analysis
  - Echocardiograms
  - Comprehensive Laboratory Animal Monitoring System
  - Intraperitoneal Glucose Tolerance Test (IPGTT)
  - Tissue collection
  - Blood Biochemistry and ELISA analysis
  - Mitochondrial stress test in MEFs
  - Respiratory chain complex activities
  - Western blots analysis
  - Real-Time Quantitative PCR
  - Prediction of WARS2 3D structure
- QUANTIFICATION AND STATISTICAL ANALYSIS

SUPPLEMENTAL INFORMATION

Supplemental Information includes seven figures and two tables and can be found with this article online at https://doi.org/10.1016/j.celrep.2018.11.080.

ACKNOWLEDGMENTS

We thank Gavin Falkous for technical assistance. T.A. is supported by a Medical Research Council UK (MRC) doctoral training studentship. M.G., C.A., A.M., M.S., H.H., C.E., Y.W., H.C., L.B., C.S., S.D.M.B., M.R.B., and R.D.C. are supported by MRC funding (MC_U142661184 and MC_U142684175). R.W.T. is supported by the Wellcome Centre for Mitochondrial Research (203105/Z/16/Z), the Medical Research Council (MRC) Centre for Translational Research in Neuromuscular Disease, Mitochondrial Disease Patient Cohort

(UK) (G0800674), the MRC/EPSRC Molecular Pathology Node, and the UK NHS Highly Specialised Service for Rare Mitochondrial Disorders of Adults and Children. R.W.T. and K.T. receive additional funding support from the Lily Foundation.

AUTHOR CONTRIBUTIONS

Conceptualization, T.A., M.G., J.P., K.J.M., S.D.M.B., R.W.T., M.R.B., and R.D.C.; Investigation, T.A., M.G., C.A., A.M., K.T., L.H., C.S., H.C., L.B., H.H., C.E., and Y.W.; Validation, Y.W.; Formal Analysis, T.A., M.S., R.D.C., and M.R.B.; Writing, Reviewing, and Editing, T.A., R.W.T., M.R.B., and R.D.C.

DECLARATION OF INTERESTS

The authors declare no competing interests.

Received: April 18, 2018
Revised: July 6, 2018
Accepted: November 21, 2018
Published: December 18, 2018

REFERENCES

Baker, B.M., Nargund, A.M., Sun, T., and Haynes, C.M. (2012). Protective coupling of mitochondrial function and protein synthesis via the eIF2α kinase GCN-2. PLoS Genet. *8*, e1002760.

Baruffini, E., Dallabona, C., Invernizzi, F., Yarham, J.W., Melchionda, L., Blakely, E.L., Lamantea, E., Donnini, C., Santra, S., Vijayaraghavan, S., et al. (2013). MTO1 mutations are associated with hypertrophic cardiomyopathy and lactic acidosis and cause respiratory chain deficiency in humans and yeast. Hum. Mutat. *34*, 1501–1509.

Bastin, J., Aubey, F., Rötig, A., Munnich, A., and Djouadi, F. (2008). Activation of peroxisome proliferator-activated receptor pathway stimulates the mitochondrial respiratory chain and can correct deficiencies in patients' cells lacking its components. J. Clin. Endocrinol. Metab. *93*, 1433–1441.

Belostotsky, R., Ben-Shalom, E., Rinat, C., Becker-Cohen, R., Feinstein, S., Zeligson, S., Segel, R., Elpeleg, O., Nassar, S., and Frishberg, Y. (2011). Mutations in the mitochondrial seryl-tRNA synthetase cause hyperuricemia, pulmonary hypertension, renal failure in infancy and alkalosis, HUPRA syndrome. Am. J. Hum. Genet. *88*, 193–200.

Burke, E.A., Frucht, S.J., Thompson, K., Wolfe, L.A., Yokoyama, T., Bertoni, M., Huang, Y., Sincan, M., Adams, D.R., Taylor, R.W., et al. (2018). Biallelic mutations in mitochondrial tryptophanyl-tRNA synthetase cause Levodopa-responsive infantile-onset Parkinsonism. Clin. Genet. *93*, 712–718.

De Sousa-Coelho, A.L., Marrero, P.F., and Haro, D. (2012). Activating transcription factor 4-dependent induction of FGF21 during amino acid deprivation. Biochem. J. *443*, 165–171.

Dickinson, M.E., Flenniken, A.M., Ji, X., Teboul, L., Wong, M.D., White, J.K., Meehan, T.F., Weninger, W.J., Westerberg, H., Adissu, H., et al.; International Mouse Phenotyping Consortium; Jackson Laboratory; Infrastructure Nationale PHENOMIN, Institut Clinique de la Souris (ICS); Charles River Laboratories; MRC Harwell; Toronto Centre for Phenogenomics; Wellcome Trust Sanger Institute; RIKEN BioResource Center (2016). High-throughput discovery of novel developmental phenotypes. Nature *537*, 508–514.

Dogan, S.A., Pujol, C., Maiti, P., Kukat, A., Wang, S., Hermans, S., Senft, K., Wibom, R., Rugarli, E.I., and Trifunovic, A. (2014). Tissue-specific loss of DARS2 activates stress responses independently of respiratory chain deficiency in the heart. Cell Metab. *19*, 458–469.

Fisher, F.M., Kleiner, S., Douris, N., Fox, E.C., Mepani, R.J., Verdeguer, F., Wu, J., Kharitonenkov, A., Flier, J.S., Maratos-Flier, E., and Spiegelman, B.M. (2012). FGF21 regulates PGC-1α and browning of white adipose tissues in adaptive thermogenesis. Genes Dev. *26*, 271–281.

Götz, A., Tyynismaa, H., Euro, L., Ellonen, P., Hyötyläinen, T., Ojala, T., Hämäläinen, R.H., Tommiska, J., Raivio, T., Oresic, M., et al. (2011). Exome sequencing identifies mitochondrial alanyl-tRNA synthetase mutations in infantile mitochondrial cardiomyopathy. Am. J. Hum. Genet. *88*, 635–642.

Haack, T.B., Kopajtich, R., Freisinger, P., Wieland, T., Rorbach, J., Nicholls, T.J., Baruffini, E., Walther, A., Danhauser, K., Zimmermann, F.A., et al. (2013). ELAC2 mutations cause a mitochondrial RNA processing defect associated with hypertrophic cardiomyopathy. Am. J. Hum. Genet. *93*, 211–223.

Hardisty-Hughes, R.E., Parker, A., and Brown, S.D. (2010). A hearing and vestibular phenotyping pipeline to identify mouse mutants with hearing impairment. Nat. Protoc. *5*, 177–190.

Hebsgaard, S.M., Korning, P.G., Tolstrup, N., Engelbrecht, J., Rouzé, P., and Brunak, S. (1996). Splice site prediction in *Arabidopsis thaliana* pre-mRNA by combining local and global sequence information. Nucleic Acids Res. *24*, 3439–3452.

Heid, I.M., Jackson, A.U., Randall, J.C., Winkler, T.W., Qi, L., Steinthorsdottir, V., Thorleifsson, G., Zillikens, M.C., Speliotes, E.K., Mägi, R., et al.; MAGIC (2010). Meta-analysis identifies 13 new loci associated with waist-hip ratio and reveals sexual dimorphism in the genetic basis of fat distribution. Nat. Genet. *42*, 949–960.

Inagaki, T., Dutchak, P., Zhao, G., Ding, X., Gautron, L., Parameswara, V., Li, Y., Goetz, R., Mohammadi, M., Esser, V., et al. (2007). Endocrine regulation of the fasting response by PPARalpha-mediated induction of fibroblast growth factor 21. Cell Metab. *5*, 415–425.

Keane, T.M., Goodstadt, L., Danecek, P., White, M.A., Wong, K., Yalcin, B., Heger, A., Agam, A., Slater, G., Goodson, M., et al. (2011). Mouse genomic variation and its effect on phenotypes and gene regulation. Nature *477*, 289–294.

Kelley, L.A., Mezulis, S., Yates, C.M., Wass, M.N., and Sternberg, M.J. (2015). The Phyre2 web portal for protein modeling, prediction and analysis. Nat. Protoc. *10*, 845–858.

Khan, N.A., Auranen, M., Paetau, I., Pirinen, E., Euro, L., Forsström, S., Pasila, L., Velagapudi, V., Carroll, C.J., Auwerx, J., and Suomalainen, A. (2014). Effective treatment of mitochondrial myopathy by nicotinamide riboside, a vitamin B3. EMBO Mol. Med. *6*, 721–731.

Kharitonenkov, A., Shiyanova, T.L., Koester, A., Ford, A.M., Micanovic, R., Galbreath, E.J., Sandusky, G.E., Hammond, L.J., Moyers, J.S., Owens, R.A., et al. (2005). FGF-21 as a novel metabolic regulator. J. Clin. Invest. *115*, 1627–1635.

Kim, K.H., Jeong, Y.T., Oh, H., Kim, S.H., Cho, J.M., Kim, Y.N., Kim, S.S., Kim, D.H., Hur, K.Y., Kim, H.K., et al. (2013). Autophagy deficiency leads to protection from obesity and insulin resistance by inducing Fgf21 as a mitokine. Nat. Med. *19*, 83–92.

Kirby, D.M., Thorburn, D.R., Turnbull, D.M., and Taylor, R.W. (2007). Biochemical assays of respiratory chain complex activity. Methods Cell Biol. *80*, 93–119.

Konovalova, S., and Tyynismaa, H. (2013). Mitochondrial aminoacyl-tRNA synthetases in human disease. Mol. Genet. Metab. *108*, 206–211.

Kopajtich, R., Nicholls, T.J., Rorbach, J., Metodiev, M.D., Freisinger, P., Mandel, H., Vanlander, A., Ghezzi, D., Carrozzo, R., Taylor, R.W., et al. (2014). Mutations in GTPBP3 cause a mitochondrial translation defect associated with hypertrophic cardiomyopathy, lactic acidosis, and encephalopathy. Am. J. Hum. Genet. *95*, 708–720.

Li, H., and Durbin, R. (2009). Fast and accurate short read alignment with Burrows-Wheeler transform. Bioinformatics *25*, 1754–1760.

Lu, P.D., Harding, H.P., and Ron, D. (2004). Translation reinitiation at alternative open reading frames regulates gene expression in an integrated stress response. J. Cell Biol. *167*, 27–33.

Mashili, F.L., Austin, R.L., Deshmukh, A.S., Fritz, T., Caidahl, K., Bergdahl, K., Zierath, J.R., Chibalin, A.V., Moller, D.E., Kharitonenkov, A., and Krook, A. (2011). Direct effects of FGF21 on glucose uptake in human skeletal muscle: implications for type 2 diabetes and obesity. Diabetes Metab. Res. Rev. *27*, 286–297.

McMurray, F., Church, C.D., Larder, R., Nicholson, G., Wells, S., Teboul, L., Tung, Y.C., Rimmington, D., Bosch, F., Jimenez, V., et al. (2013). Adult onset

global loss of the fto gene alters body composition and metabolism in the mouse. PLoS Genet. *9*, e1003166.

Michel, S., Canonne, M., Arnould, T., and Renard, P. (2015). Inhibition of mitochondrial genome expression triggers the activation of CHOP-10 by a cell signaling dependent on the integrated stress response but not the mitochondrial unfolded protein response. Mitochondrion *21*, 58–68.

Musante, L., Püttmann, L., Kahrizi, K., Garshasbi, M., Hu, H., Stehr, H., Lipkowitz, B., Otto, S., Jensen, L.R., Tzschach, A., et al. (2017). Mutations of the aminoacyl-tRNA-synthetases SARS and WARS2 are implicated in the etiology of autosomal recessive intellectual disability. Hum. Mutat. *38*, 621–636.

Nunnari, J., and Suomalainen, A. (2012). Mitochondria: in sickness and in health. Cell *148*, 1145–1159.

Oprescu, S.N., Griffin, L.B., Beg, A.A., and Antonellis, A. (2017). Predicting the pathogenicity of aminoacyl-tRNA synthetase mutations. Methods *113*, 139–151.

Pierce, S.B., Chisholm, K.M., Lynch, E.D., Lee, M.K., Walsh, T., Opitz, J.M., Li, W., Klevit, R.E., and King, M.C. (2011). Mutations in mitochondrial histidyl tRNA synthetase HARS2 cause ovarian dysgenesis and sensorineural hearing loss of Perrault syndrome. Proc. Natl. Acad. Sci. USA *108*, 6543–6548.

Pierce, S.B., Gersak, K., Michaelson-Cohen, R., Walsh, T., Lee, M.K., Malach, D., Klevit, R.E., King, M.C., and Levy-Lahad, E. (2013). Mutations in LARS2, encoding mitochondrial leucyl-tRNA synthetase, lead to premature ovarian failure and hearing loss in Perrault syndrome. Am. J. Hum. Genet. *92*, 614–620.

Potter, P.K., Bowl, M.R., Jeyarajan, P., Wisby, L., Blease, A., Goldsworthy, M.E., Simon, M.M., Greenaway, S., Michel, V., Barnard, A., et al. (2016). Novel gene function revealed by mouse mutagenesis screens for models of age-related disease. Nat. Commun. *7*, 12444.

Pravenec, M., Zídek, V., Landa, V., Mlejnek, P., Šilhavý, J., Šimáková, M., Trnovská, J., Škop, V., Marková, I., Malínská, H., et al. (2017). Mutant Wars2 gene in spontaneously hypertensive rats impairs brown adipose tissue function and predisposes to visceral obesity. Physiol. Res. *66*, 917–924.

Rath, E., Berger, E., Messlik, A., Nunes, T., Liu, B., Kim, S.C., Hoogenraad, N., Sans, M., Sartor, R.B., and Haller, D. (2012). Induction of dsRNA-activated protein kinase links mitochondrial unfolded protein response to the pathogenesis of intestinal inflammation. Gut *61*, 1269–1278.

Riley, L.G., Cooper, S., Hickey, P., Rudinger-Thirion, J., McKenzie, M., Compton, A., Lim, S.C., Thorburn, D., Ryan, M.T., Giegé, R., et al. (2010). Mutation of the mitochondrial tyrosyl-tRNA synthetase gene, YARS2, causes myopathy, lactic acidosis, and sideroblastic anemia—MLASA syndrome. Am. J. Hum. Genet. *87*, 52–59.

Rivera, H., Martín-Hernández, E., Delmiro, A., García-Silva, M.T., Quijada-Fraile, P., Muley, R., Arenas, J., Martín, M.A., and Martínez-Azorín, F. (2013). A new mutation in the gene encoding mitochondrial seryl-tRNA synthetase as a cause of HUPRA syndrome. BMC Nephrol. *14*, 195.

Schneider, C.A., Rasband, W.S., and Eliceiri, K.W. (2012). NIH Image to ImageJ: 25 years of image analysis. Nat. Methods *9*, 671–675.

Shahni, R., Wedatilake, Y., Cleary, M.A., Lindley, K.J., Sibson, K.R., and Rahman, S. (2013). A distinct mitochondrial myopathy, lactic acidosis and sideroblastic anemia (MLASA) phenotype associates with YARS2 mutations. Am. J. Med. Genet. A. *161A*, 2334–2338.

Simon, M., Richard, E.M., Wang, X., Shahzad, M., Huang, V.H., Qaiser, T.A., Potluri, P., Mahl, S.E., Davila, A., Nazli, S., et al. (2015). Mutations of human NARS2, encoding the mitochondrial asparaginyl-tRNA synthetase, cause nonsyndromic deafness and Leigh syndrome. PLoS Genet. *11*, e1005097.

Sofou, K., Kollberg, G., Holmström, M., Dávila, M., Darin, N., Gustafsson, C.M., Holme, E., Oldfors, A., Tulinius, M., and Asin-Cayuela, J. (2015). Whole exome sequencing reveals mutations in NARS2 and PARS2, encoding the mitochondrial asparaginyl-tRNA synthetase and prolyl-tRNA synthetase, in patients with Alpers syndrome. Mol. Genet. Genomic Med. *3*, 59–68.

Soldà, G., Caccia, S., Robusto, M., Chiereghin, C., Castorina, P., Ambrosetti, U., Duga, S., and Asselta, R. (2016). First independent replication of the involvement of LARS2 in Perrault syndrome by whole-exome sequencing of an Italian family. J. Hum. Genet. *61*, 295–300.

Steenweg, M.E., Ghezzi, D., Haack, T., Abbink, T.E., Martinelli, D., van Berkel, C.G., Bley, A., Diogo, L., Grillo, E., Te Water Naudé, J., et al. (2012). Leukoencephalopathy with thalamus and brainstem involvement and high lactate 'LTBL' caused by EARS2 mutations. Brain *135*, 1387–1394.

Theisen, B.E., Rumyantseva, A., Cohen, J.S., Alcaraz, W.A., Shinde, D.N., Tang, S., Srivastava, S., Pevsner, J., Trifunovic, A., and Fatemi, A. (2017). Deficiency of WARS2, encoding mitochondrial tryptophanyl tRNA synthetase, causes severe infantile onset leukoencephalopathy. Am. J. Med. Genet. A. *173*, 2505–2510.

Ü Basmanav, F.B., Cau, L., Tafazzoli, A., Méchin, M.C., Wolf, S., Romano, M.T., Valentin, F., Wiegmann, H., Huchenq, A., Kandil, R., et al. (2016). Mutations in three genes encoding proteins involved in hair shaft formation cause uncombable hair syndrome. Am. J. Hum. Genet. *99*, 1292–1304.

Vantroys, E., Smet, J., Vanlander, A.V., Vergult, S., De Bruyne, R., Roels, F., Stepman, H., Roeyers, H., Menten, B., and Van Coster, R. (2018). Severe hepatopathy and neurological deterioration after start of valproate treatment in a 6-year-old child with mitochondrial tryptophanyl-tRNA synthetase deficiency. Orphanet J. Rare Dis. *13*, 80.

Wall, C.E., Whyte, J., Suh, J.M., Fan, W., Collins, B., Liddle, C., Yu, R.T., Atkins, A.R., Naviaux, J.C., Li, K., et al. (2015). High-fat diet and FGF21 cooperatively promote aerobic thermogenesis in mtDNA mutator mice. Proc. Natl. Acad. Sci. USA *112*, 8714–8719.

Wang, M., Sips, P., Khin, E., Rotival, M., Sun, X., Ahmed, R., Widjaja, A.A., Schafer, S., Yusoff, P., Choksi, P.K., et al. (2016). Wars2 is a determinant of angiogenesis. Nat. Commun. *7*, 12061.

Wolny, S., McFarland, R., Chinnery, P., and Cheetham, T. (2009). Abnormal growth in mitochondrial disease. Acta Paediatr. *98*, 553–554.

Wortmann, S.B., Timal, S., Venselaar, H., Wintjes, L.T., Kopajtich, R., Feichtinger, R.G., Onnekink, C., Mühlmeister, M., Brandt, U., Smeitink, J.A., et al. (2017). Biallelic variants in WARS2 encoding mitochondrial tryptophanyl-tRNA synthase in six individuals with mitochondrial encephalopathy. Hum. Mutat. *38*, 1786–1795.

STAR★METHODS

KEY RESOURCES TABLE

REAGENT or RESOURCE	SOURCE	IDENTIFIER
Antibodies		
WARS2 – rabbit polyclonal by Covolab	This paper	N/A
Mouse monoclonal anti-NDUFB8	Abcam	Cat#ab110242; CLONE No: 20-E9DH10C12; RRID: AB_10859122
Mouse monoclonal anti-SDHA	Abcam	Cat#ab14715; CLONE No: 2E3GC12FBZAE2; RRID:AB_301433
Mouse monoclonal anti-UQCRC2	Abcam	Cat#ab14745; CLONE No: 13G12AF12BB11; RRID:AB_2213640
Mouse monoclonal anti-MTCO1	Abcam	Cat#ab14705; CLONE No: 1D6E1A8; RRID:AB_2084810
Mouse monoclonal anti-ATP5A	Abcam	Cat#ab14748; CLONE No: 15H4C4; RRID:AB_301447
Rabbit monoclonal anti-eIF2α	Cell signaling	Cat#5324; CLONE No: D7D3; RRID:AB_10692650
Rabbit monoclonal anti-phosphor-Ser51-eIF2α	Epitomics	Cat#1090-1/ab32157; CLONE No: E90; RRID:AB_732117
Rabbit polyclonal anti-ATF4	Santa Cruz	Cat#sc-22800; LOT No: CREB-2(H-290); RRID:AB_2058742
Rabbit polyclonal anti-LONP1	Abcam	Cat#ab103809; RRID:AB_10858161
Rabbit monoclonal anti-CLPP	Abcam	Cat#ab124822; CLONE No: EPR7133; RRID:AB_10975619
Rabbit polyclonal anti-HSP60	Abcam	Cat#ab46798; RRID:AB_881444
Mouse monoclonal anti-HSP70	Abcam	Cat#ab2799; CLONE No:JG1; RRID:AB_303311
Goat polyclonal anti-UCP1	Santa Cruz	Cat#sc-6529; LOT No: M17; RRID:AB_2213781
Mouse monoclonal anti-Actin	Millipore	Cat#MAB1501; CLONE No: C4; RRID:AB_2223041
Rabbit polyclonal anti-α-Tubulin	Cell Signaling	Cat#2144
Mouse monoclonal anti-GAPDH	Abcam	Cat#ab8245; CLONE No: 6C5; RRID:AB_2107448
Total mouse monoclonal anti-OXPHOS rodent WB antibody cocktail (5 monoclonal antibodies)	Abcam	Cat#ab110413; RRID:AB_2629281
Chemicals, Peptides, and Recombinant Proteins		
Seahorse XF Cell Mito Stress Test Kit	Agilent	Cat#103015-100
Critical Commercial Assays		
Plasma FGF21 Quantikine ELISA Mouse / Rat FGF-21 Immunoassays (ELISA)	R&D Systems	Cat#MF2100
Plasma Free fatty Acids	Alpha Labs	Reagent 1- 434-91795/ Reagent 2- 436-91995
Plasma Glucose	Beckman Coulter	OSR6121
Plasma triglycerides	Beckman Coulter	OSR61118
D-3-hydroxybutyrate	Randox	RB 1007
Blood Glucose (IPGTT) – Abbott Alphatrak strips	Larkmead Veterinary	N/A

(*Continued on next page*)

Continued

REAGENT or RESOURCE	SOURCE	IDENTIFIER
Deposited Data		
Reference mouse genome sequence NCBI Mouse Build 38, mm10	Genome Reference Consortium	https://www.ncbi.nlm.nih.gov/assembly/GCF_000001635.20/
WARS2 (5EKD, Human mitochondrial tryptophanyl-tRNA synthetase bound by indolmycin and Mn*ATP. Williams, T.L., Carter Jr., C.W.)	PDB	http://www.rcsb.org/
Experimental Models: Cell Lines		
Primary Mouse Embryo Fibroblasts (MEFS), wildtype and *Wars2*$^{V117L/V117L}$	This paper	N/A
Experimental Models: Organisms/Strains		
Mouse *Wars2*:V117L	This paper and Potter et al., 2016	N/A
Mouse *Wars2:tm1(KOMP)Vlcg*	UCDAVIS KOMP Repository	KOMP: VG15335
C3H/Pde (Pde6b+ repaired mice)	MRC Harwell Institute	N/A
C57BL/6J	MRC Harwell Institute	JAX:000664, RRID:IMSR_JAX:000664
Oligonucleotides		
Primers for Genotyping, see Table S2	This Paper	N/A
Wars2 (Exon 2-3) (Mm04208965_m1)	ThermoFisher	CAT#: 4351372
Wars2 (Exon 4-5) (Mm04208967_m1)	ThermoFisher	CAT#: 4351372
Wars2 (Exon 5-6) (Mm00840490_m1)	ThermoFisher	CAT#: 4331182
Pgc1α (Mm01208835_m1)	ThermoFisher	CAT#: 4331182
Atf4 (Mm00515324_m1)	ThermoFisher	CAT#: 4331182
Atf5 (Mm00459515_m1)	ThermoFisher	CAT#: 4331182
Chop (Mm01135937_g1)	ThermoFisher	CAT#: 4331182
Fgf21 (Mm00840165_g1)	ThermoFisher	CAT#: 4331182
Tfam (Mm00447485_m1)	ThermoFisher	CAT#: 4331182
Pparα (Mm00440939_m1)	ThermoFisher	CAT#: 4331182
Ucp1 (Mm01244861_m1)	ThermoFisher	CAT#: 4331182
Dio2 (Mm00515664_m1)	ThermoFisher	CAT#: 4331182
Cidea (Mm00432554_m1)	ThermoFisher	CAT#: 4331182
Pparγ (Mm00440945_m1)	ThermoFisher	CAT#: 4331182
Cox7a1 (Mm00438297_g1)	ThermoFisher	CAT#: 4331182
Cox8b (Mm00432648_m1)	ThermoFisher	CAT#: 4331182
Software and Algorithms		
GenotypeCaller tool in the Genome Analysis Toolkit (GATK)	Potter et al., 2016	https://software.broadinstitute.org/gatk/
Phyre2	Kelley et al., 2015	http://www.sbg.bio.ic.ac.uk/~phyre2/html/page.cgi?id=index
NetGene2	Hebsgaard et al., 1996	http://www.cbs.dtu.dk/services/NetGene2/
PyMOL by schrodinger	Schrodinger	https://pymol.org/2/
ImageJ 1.8.0_172	Schneider et al., 2012	https://imagej.nih.gov/ij/
SPSS	IBM	https://www.ibm.com/uk-en/products/spss-statistics
Graphpad PRISM v7.0d	GraphPad	https://www.graphpad.com/scientific-software/prism/

CONTACT FOR REAGENT AND RESOURCE SHARING

Further information and requests for resources and reagents should be directed to and will be fulfilled by the Lead Contact, Roger Cox (r.cox@har.mrc.ac.uk).

EXPERIMENTAL MODEL AND SUBJECT DETAILS

Animal Models

All mice used in this study were housed in the Mary Lyon Centre at MRC Harwell. Mice were kept and studied in accordance with UK Home Office legislation and local ethical guidelines issued by the Medical Research Council (Responsibility in the Use of Animals for Medical Research, July 1993; Home Office license 30/3146 and 30/3070). Procedures were approved by the MRC Harwell Animal Welfare and Ethical Review Board (AWERB). Mice were kept under controlled light (light 7am–7pm, dark 7pm–7am), temperature (21 ± 2°C) and humidity (55 ± 10%) conditions. They had free access to water (9–13 ppm chlorine) and were fed *ad libitum* on a commercial diet (SDS Rat and Mouse No. 3 Breeding diet, RM3, 3.6 kcal/g).

MPC-151 pedigree was generated from The Harwell Aging ENU-mutagenesis Screen as documented previously (Potter et al., 2016). These mice are C57BL/6J mutagenized mice crossed with C3H/Pde (Pde6b+ repaired mice) and subsequently maintained by backcrossing to C3H/Pde mice. Age and sex of mice is indicated in the figure legends. Estimates for required cohort sizes were made using GraphPad Statmate using trait data from previous experiments.

Cohorts of male and female mice were bred for longitudinal blood and body composition-based phenotyping tests. Four cohorts of mice $Wars2^{V117L/V117L}$ mice were generated from $Wars2^{+/V117L}$ x $Wars2^{+/V117L}$ matings and were aged to 1- (20 mice total), 3- (58 mice total), 9- (74 mice total) and 12 months (58 mice total) of age before being humanely killed in accordance with Home Office schedule 1 regulations. $Wars2^{V117L/-}$ mice were generated from $Wars2^{+/-}$ x $Wars2^{V117L/+}$ matings (78 mice total). $Ppa2^{Y123F/Y123F}$ mice were generated from $Ppa2^{+/Y123F}$ x $Ppa2^{+/Y123F}$ matings (47 mice total). Mice were randomly assigned to cages at weaning before subsequent genotyping of individual mice. Downstream phenotyping experiments were performed blinded to the genotype of the mice.

For body composition three cohorts were analyzed, two with multiple time points and one at 1 month only. In the first cohort one wild-type mouse was humanely killed because it was sick and one found dead and all data from these animals was excluded. Final cohort sizes were $Wars2^{+/+}$ n = 11 and 3, $Wars2^{+/V117L}$ n = 13 and 17, $Wars2^{V117L/V117L}$ n = 7 and 8, male and female respectively. In the second cohort data from one homozygous mouse was excluded after being found dead before the 6-month time-point and two heterozygous mice humanely killed to reduce cage numbers prior to starting phenotyping. Final cohort sizes were $Wars2^{+/+}$ n = 13 and 18, $Wars2^{+/V117L}$ n = 19 and 19, $Wars2^{V117L/V117L}$ n = 11 and 7, male and female respectively. In cohort 3 there were $Wars2^{+/+}$ n = 4, $Wars2^{+/V117L}$ n = 15, $Wars2^{V117L/V117L}$ n = 9 and none of the differences for body weight, fat mass or lean mass were significant (tested at one month only).

An additional fifth intercross cohort, congenic on C3H/Pde, was generated for additional replication experiments including OXPHOS blots and FGF21 measurements in plasma at 3-4 months.

The NIH KOMP *Wars2*-KO allele ($Wars2^{tm1(KOMP)Vlcg}$) obtained from the KOMP repository (https://www.komp.org/) comprises a targeting construct integrated into the C57BL/6N ES cell genome by homologous recombination, deleting 46632bp of the *Wars2* gene locus, including coding regions of both *Wars2*-Exon1 and *Wars2*-Exon2, leading to a frameshift and a premature stop codon. $Wars2^{tm1(KOMP)Vlcg}$ ES cells were micro-injected into C57BL/6N blastocysts generating mosaic C57BL/6N-$Wars2^{tm1(KOMP)Vlcg}$ offspring. Germ-line transmission (GLT) of the $Wars2^{tm1(KOMP)Vlcg}$ construct was determined by genotyping C57BL/6N-$Wars2^{tm1(KOMP)Vlcg}$ x C57BL/6N offspring for the neomycin selection cassette.

Primary Cultures

MEFs were harvested from E12.5-14 (dpc) embryos from timed $Wars2^{+/V117L}$ x $Wars2^{+/V117L}$ matings and dissected on ice in Dulbeccos PBS (ThermoFischer 14190094). The sex of the embryos was unknown. Head, liver, heart and limbs were placed in 3ml GIBCO 0.25% trypsin (EDTA) (ThermoFischer 25200056) and minced using surgical scissors and then pipetted 10x using a P1000 pipette and sterile filter tip, before transfer to a 15ml Falcon tube and incubated at 37°C for 10 minutes. The trypsin was neutralized with 7ml of culture medium, DMEM (ThermoFischer 31966021) supplemented with 1 X NEAA (Sigma-Aldrich M7145), 1 X Penicillin/Streptomycin (ThermoFischer 15070-063), 50 μM 2-mercaptoethanol (2-mercaptoethanol (ThermoFischer 31350010) and 10% GIBCO FBS (ThermoFischer 10500064). Cells were then plated on a 10cm dish and incubated at 37°C and 5% CO_2.

METHOD DETAILS

SNP Mapping and Whole Genome Sequencing

SNP mapping and NGS were performed as described previously (Potter et al., 2016). Briefly Individual mutations were mapped using the Illumina GoldenGate Mouse Medium Density Linkage Panel (Gen-Probe Life Sciences Ltd, UK) that utilizes over 900 SNPs for the C3H/Pde (Pde6b+ repaired mice) and C57BL/6J strains. The genotypes of G3 'affected' (elevated ABR thresholds) MPC-151 mice were compared to 'non-affected' ('normal' ABR thresholds) littermate controls. This allowed us to identify a 75Mb region within which all 'affected' MPC-151 mice were homozygous for C57BL/6J SNPs and 'non-affected' MPC-151 littermates were either heterozygous or homozygous for C3H.Pde6b+ SNPs. To identify candidate causal ENU-induced mutations within the mapped region, WGS was performed using DNA from an 'affected' G3 MPC-151 mouse. WGS was performed as previously described (Potter et al., 2016). Briefly, following DNA extraction a library was generated and a single lane or paired-end sequencing (100nt) was performed using the Illumina HiSeq platform (Oxford Genomics Centre, Wellcome Centre for Human Genetics). The 100nt paired-end

reads were aligned to the reference mouse genome (NCBIM38/mm10) using Burrows-Wheeler Aligner software (Li and Durbin, 2009). Single-nucleotide variants (SNVs) were identified for each alignment using the unified GenotypeCaller tool in the Genome Analysis Toolkit (GATK) as previously described (Potter et al., 2016). Here the mouse dbSNP version 137 was used as the background SNP set using default parameters. Identified SNVs were then given a quality score (Phred scaled quality score, -10 x log(1-p), p is the probability of a SNV being called incorrectly). SNVs with a quality score of < 100 or with a read depth of < 3 reads were removed from all further analysis. All remaining SNVs were termed 'high-confidence' mutations and were compared to previously identified SNPs from 17 inbred strains from the Mouse Genome Project (Keane et al., 2011) as well as an in-house library of SNVs. Any overlapping sites were removed leaving the final list of novel ENU-induced SNVs for the 'affected' MPC-151 G3 mouse. SNVs were annotated using NGS-SNP to give an indication of the nature of the SNV (e.g., Missense, splice-site variant or intronic). 3 high-confidence, ENU-induced, missense mutations were identified for the MPC-151 G3 'affected' mouse that were located within the 75Mb mapped region as previously identified.

Genotyping

Mice were assayed for the presence or absence of ENU-induced mutations $Ppa2^{A398T}$ and $Wars2^{G349T}$ by pyrosequencing (Potter et al., 2016). PCR primers were designed to amplify the regions of interest using a biotinylated primer for the Pyrosequencing template strand. $Ppa2^{A398T}$ primers: biotinylated forward (5′- CTCAATCCCATTAAGCAAGATAT-3′), reverse (5′-GGTTTCTGTAGAAGG CATAAAAG-3′) and sequencing reverse (5′-GGGAAGATGTTCGGTG-3′). $Wars2^{G349T}$ primers: forward (5′-GGTCACCTTTCTTT CTCTCC-3′), biotinylated reverse (5′-CAGGTGAGGATCCAACTTAA-3′) and forward sequencing (5′-TTTCTCTCCTTCCTTTTAG-3′).

Mice generated from $Wars2^{V117L/-}$ x $Wars2^{+/-}$ matings were genotyped using two strategies. The $Wars2^{V117L}$ allele was genotyped using the Idaho Technology LightScanner System (Idaho Technology Inc, Utah, USA) and was used in accordance with the manufacturers standard protocols. $Wars2^{V117L}$ Primers: forward (5′-TCAGCCTATCCCTGTTGTCTA-3′), reverse (5′-TGG TGTAAATGCTGCAATCG-3′) and probe (5′-CCTTCCTTTTAGTTGTCTGAACACACTCAG-3′). The *Wars2*-KO allele was genotyped for the presence of the LacZ reporter cassette using a RT-PCR copy number assay using FAM-labeled taqman probes. Assays were performed using FAM-labeled TaqMan probes for LacZ and *Wars2*-WT DNA sequences as controls. Each assay was performed along with an additional VIC-labeled TaqMan probe designed to Dot1l that acted as an internal controls. $Wars2^{WT}$ primers: forward (5′-GCCCAGCACTTGGGATGT-3′) and reverse (5′-GCAGCCAGCTCACCAATG-3′), FAM labeled probe (5′-TCCCTTCACTTTCCTGTCTCCGTTTC-3′). LacZ primers: forward (5′-CTCGCCACTTCAACATCAAC-3′), reverse (5′-TTAT CAGCCGGAAAACCTACC-3′), FAM labeled probe (5′-TCGCCATTTGACCACTACCATCAATCC-3′). Dot1l primers: forward (5′-GCCCCAGCACGACCATT-3′), reverse (5′-TAGTTGGCATCCTTATGCTTCATC-3′) and VIC labeled probe (5′-CCAGCTCT CAAGTCG-3′).

Auditory phenotyping

Click box protocol as previously described (Hardisty-Hughes et al., 2010). Briefly, mice were placed on the operator's palm and hearing was tested using a purpose built frequency calibrated click box (CB) that emits a 90 dB SPL tone at 20 kHz (CB apparatus was obtained from MRC Institute of Hearing Research, Nottingham, UK). The CB emits a tone that elicits a Preyer reflex from the mouse as seen by a visible flick of the pinna or a startle response if the mouse can hear. The presence or absence of a Preyer reflex is then scored as follows: 2 – normal startle response, 1 – reduced startle, 0 – no startle. CB testing was performed blinded and away from the home cage to prevent littermates from becoming attenuated to the CB tone. Auditory-evoked brainstem response (ABR) testing was performed as previously described (Hardisty-Hughes et al., 2010). Briefly, mice were anaesthetized via administration of an intra-peritoneal injection of anesthetic (1 mL Ketamine, 0.5 mL Xylasine, 8.5 mL sterile H_2O) at a rate of 0.1 ml/10 g of body mass. Once unconscious, the mouse was placed on a heated mat in a sound proof booth. Electrodes were then placed sub-dermally below the right pinna (reference), into the muscle mass below the left ear (ground) and on the midline of the skull (active). Mice were placed with their auditory canal 1 cm from the speaker and were exposed to a broadband 'click' stimulus, followed by tones at 8, 16 and 32 kHz. The electrodes recorded the auditory brainstem responses to the tones. The recorded data was calibrated, generated and processed using the Tucker Davies Technology (TDT) system III. Following the ABR testing an IP injection of Antipamezole (0.1 mL in 9.9 mL of sterile water) at a rate of 0.1 mL < 50 g or 0.2 mL > 50 total body mass was administered to reverse the anesthetic.

Body weight and composition analysis

Body mass was measured monthly on scales calibrated to 0.01 g. Body composition was measured monthly using an Echo-MRI quantitative NMR machine (Echo-MRI-100, Echo-MRI, Texas, U.S.A.).

Echocardiograms

Mice were placed under general anesthetic using 4% isoflurane using an anesthetic chamber. Once unconscious the mouse was placed on an ECG platform (Visualsonics heatpad / ECG platform) and mouse limbs are taped to ECG probes to allow heart rate monitoring. Anesthesia was maintained using a nose cone and 1.5% (or as appropriate to maintain a heart rate < 400 bpm) isoflurane. Hair was removed from the mouse chest using hair clippers followed by hair removal cream. A rectal thermometer was inserted and used to monitor core body temperature throughout the procedure. Contact gel was applied to the shaven mouse chest and a 707B probe was lowered to the mouse chest locating the mouse heart left ventricle until contractions of the left ventricle could be monitored

on the Visualsonics Vevo 770 high resolution *in vivo* micro imaging system. Several images of mouse heart were taken in M-mode and were analyzed using the Vevo 770 software. Following successful data capture, the rectal probe, contact gel and limb tape was removed and the mouse was placed in a heat box to recover from the anesthetic.

Comprehensive Laboratory Animal Monitoring System

The Comprehensive lab animal monitoring system (CLAMS) was used to measure mice energy expenditure at home cage temperature (22°C) according to standard protocols. Briefly mice were placed in individual cages for a total of 72 hours. Measurements of oxygen (O_2) and carbon-dioxide (CO^2) in-flow and out-flow concentrations were automatically monitored and recorded along with food consumption and water intake throughout the 72-hour period. Data from the first 24 hours was removed from the analysis as this period was used to allow the mice to acclimatize to their new environment. Data collected from the second 24-hour period was used for all subsequent analysis. Energy expenditure was calculated as follows EE = CV x VO_2 (where CV = Calorific value = 3.815 + 1.232 x RER and VO_2 = ViO_2i - VoO_2o (o = outflow, i = inflow). EE values were normalized to lean mass using multiple linear regression analysis (ANCOVA) as described previously (McMurray et al., 2013).

Intraperitoneal Glucose Tolerance Test (IPGTT)

Mice were fasted overnight and IPGTT were performed the following morning. On the morning of the IPGTT, mice were weighed and a local anesthetic was administered to the mouse tail (EMLA cream, Eutectic mixture of Local Anesthetics Lidocaine / Prilocaine, AstraZeneca, UK). A blood sample was collected from the mouse tail at time point zero in Lithium-Heparin microvette tubes (CB30, Sarstedr, Numbrecht, Germany) to establish a baseline blood glucose level. Mice were then administered an intra-peritoneal injection of 2 g glucose / kg body weight (20% glucose in 0.9% NaCl). Blood samples were then taken 60 and 120 mins post-injection. At each time point, blood glucose levels were measured using the handheld Alphatrak (Abbott) glucose monitor with a fresh Alphatrak strip (Abbott) being used for every reading.

Tissue collection

Mice were humanely killed at 1-, 3-, 9- and 12 months of age and tissues were harvested for analysis. Following confirmation of death: cochlea, heart, liver, kidney, iWAT, gonadal which adipose tissue, BAT and skeletal muscle were dissected. For subsequent protein, RNA and DNA analysis tissues were placed in cryotubes (Nunc, Thermo Fisher Scientific-Heraeus) and snap frozen in liquid nitrogen. Tissue samples were stored long-term at −70°C.

Blood Biochemistry and ELISA analysis

Food was withdrawn and mice were fasted at 8:00 AM. 4hrs later, mice were humanely killed by administration of an over-dose of anesthetic (0.2 mL of pentobarbitone) via intra-peritoneal injection in accordance with home office procedures. Once the mouse was fully anaesthetized a glass capillary is inserted into the anterior corner of the mouse eye to puncture the membrane of the retro-orbital sinus. Blood was collected from the capillary in Lithium-Heparin microvette tubes (CB30, Sarstedr, Numbrecht, Germany). Blood samples were centrifuged for 10 mins at 8000 x g at 8°C. The supernatant blood plasma was removed and analyzed on a Beckman Coulter AU680 clinical chemistry analyzer using reagents and settings recommended by the manufacturer. Plasma FGF21 proteins levels were assayed using Quantikine ELISA Mouse / Rat FGF-21 Immunoassays (R&D Systems) according to manufacturer's instructions.

Mitochondrial stress test in MEFs

Oxygen consumption rate (OCR) and extracellular acidification rate (ECAR) were measured in MEFs using the Seahorse XF24 flux analyzer (Seahorse Bioscience). Primary MEFs were seeded at a density of 40000 cells/well on XF-24 tissue culture plate and left to adhere overnight. The following day MEFs media was replaced with XF Assay Media supplemented with L-glutamine 2 mM, sodium pyruvate 2 mM, and glucose 10 mM (pH 7.4) and were incubated for 1 hour at 37°C in a CO_2 free incubator before being placed in the XF24 analyzer. OCR and ECAR measurements were measured under basal conditions and following administration of mitochondrial inhibitors oligomycin (1 μM), antimycin (1 μM) and rotenone (1 μM) or in the presence of the mitochondrial uncoupler FCCP (1 μM) (Seahorse XF Cell Mito Stress Test Kit, Agilent). Oxygen consumption rates were normalized to the number of live cells using the LIVE/DEAD Viability/Cytotoxicity kit (ThermoFisher) according to the manufactures instructions.

Respiratory chain complex activities

The activities of individual respiratory chain complex activities and citrate synthase, a mitochondrial matrix marker, were determined in skeletal muscle and cardiac muscle homogenates as previously described (Kirby et al., 2007).

Western blots analysis

Proteins were extracted from snap frozen mouse tissues using CelLyic MT Mammalian Tissue Lysis Buffer (Sigma- Aldrich) supplemented with 1 X complete protease inhibitor cocktail (1 μL / 100 μL lysis buffer, Sigma- Aldrich) and 1 X PhosStop phosphatase inhibitor cocktail (1 μL / 100 μL lysis buffer, Sigma Aldrich). Tissues were homogenized using the Precellys-24 automated homogenizer (Bertin Technologies). Tissue homogenates were centrifuged at 13,000 rpm for 15 mins at 4°C to pellet cell debris. The supernatant

tissue lysates were isolated and protein concentrations were determined using the BCA (bicinchoninic acid) Protein Assay Reagent (BioRad). Samples were diluted to 4 μg / μL in lysis buffer and supplemented with NuPAGE LDS Sample Buffer (4X) and NuPage Reducing Agent (10X) and were denatured by heating to 70°C for 10mins. Protein samples were separated using 4%–12% linear gradient Bis-Tris ready polyacrylamide gels with 1 X MOPS electrophoresis running buffer (Invitrogen) using the XCell Surelock Mini Cell tanks (Invitrogen) at 200 V for 50 mins. Protein samples were electrotransferred from the gels onto PVDF membrane (Hybond – P, GE Healthcare Amersham) using a XCell II Blot Module (Invitrogen). Protein membranes were blocked in 5% non-fat milk Tris Buffered Saline with Tween 20 (TBST, Merk) (non-phosphor antibodies) or 5% Bovine Serum Albumin TBST (phosphor-antibodies) at room temperature for an hour or overnight at 4°C before being incubated with primary antibodies overnight at 4°C. Protein membranes were washed 3-5 times in TBST for 10mins at room-temperature. Secondary antibodies were diluted in 5% non-fat milk TBST. Membranes were incubated with species-specific secondary horseradish peroxidase (HRP) conjugated antibodies for 4 hr at room-temperature. Membranes were washed 5 times in TBST for 10 mins. Immunolabelled membrane were treated with Enhanced Chemiluminesence Plus (ECL plus; Amersham, GE Healthcare) and were imaged using the ChemiDoc UV chemiluminescent imager or exposure to X-ray film.

Primary antibodies used in this study: WARS2, at a 1:500 dilution (custom, Covalab); NDUFB8, at a 1:2,000 dilution (ab110242, Abcam); SDHA, at a 1:10,000 dilution (ab14715, Abcam); UQCRC2, at a 1:3,000 dilution (ab14745, Abcam); MTCO1, at a 1:2,000 dilution (ab14705, Abcam); ATP5A, at a 1:5,000 dilution (ab14748, Abcam); eIF2α, at a 1:1000 dilution (#5324, Cell signaling); phosphor-Ser51-eIF2α, at a 1:1,000 dilution (#1090-1, Epitomics); ATF4, at a 1:500 dilution (sc-22800, Santa Cruz); LONP1, at a 1:1,000 dilution (ab103809, Abcam); CLPP, at a 1:5,000 dilution (ab124822, Abcam); HSP60, at a 1:10,000 dilution (ab46798, Abcam); HSP70, at a 1:1,000 dilution (ab2799, Abcam); UCP1, at a 1:200 dilution (sc-6529, Santa Cruz); Actin, at a 1:5,000 dilution (MAB1501, Millipore); α-Tubulin, at a 1:5,000 dilution (#2144, Cell Signaling); and GAPDH, at a 1:10,000 dilution (ab8245, Abcam).

In subsequent OXPHOS blot experiments (Figure S5) a total OXPHOS rodent WB antibody cocktail was used at a 1:1,000 dilution (ab110413, Abcam).

Real-Time Quantitative PCR

Total RNA was extracted from MEFs and mouse tissues using the RNeasy Mini Plus Kit (QIAGEN) according to the manufacture's protocol. RNA concentrations were determined using a NanoDrop spectrophotometer (Thermo Scientific). RNA samples were diluted to 200 ng/μl and reverse transcription reactions were performed using Super Script III reverse transcriptase (Invitrogen) following the manufacturer's protocol to generate 2 μg of cDNA. mRNA gene expression analysis was performed using the TaqMan system. TaqMan Gene Expression Assay reagents and TaqMan FAM dye-labeled probes (Applied Biosystems, Invitrogen, U.S.A.) were used according to the manufacturers protocol and assays were performed using an ABIPRISM 7500 Fast Real-Time PCR System (Applied Biosystems). Data was normalized to house-keeping genes specific to the tissue / cell line being used. GeNORM analysis was performed for each cell / tissue used to determine the most suitable housekeeping gene. Data were analyzed using the comparative ΔΔCT method in order to determine the difference in sample groups relative to control samples. Taqman probes used in this study: *Wars2 (Exon 2-3)* (Mm04208965_m1), *Wars2 (Exon 4-5)* (Mm04208967_m1), *Wars2 (Exon 5-6)* (Mm00840490_m1), *Pgc1α* (Mm01208835_m1), *Atf4* (Mm00515324_m1), *Atf5* (Mm00459515_m1), *Chop* (Mm01135937_g1), *Fgf21* (Mm00840165_g1), *Tfam* (Mm00447485_m1), *Pparα* (Mm00440939_m1), *Ucp1* (Mm01244861_m1), *Dio2* (Mm00515664_m1), *Cidea* (Mm00432554_m1), *Pparγ* (Mm00440945_m1), *Cox7a1* (Mm00438297_g1) and *Cox8b* (Mm00432648_m1).

Prediction of WARS2 3D structure

The crystal structure of human WARS2 (PDB: 5EKD, Human mitochondrial tryptophanyl-tRNA synthetase bound by indolmycin and Mn*ATP. Williams, T.L., Carter Jr., C.W.) was downloaded from the PDB database (PDB; http://www.rcsb.org/). The predicted protein structure of human Wars2 was generated using PHYRE2 Protein fold recognition server (Kelley et al., 2015). The alignment and visualization of the protein structures was performed by PyMOL by Schrödinger (https://pymol.org/2/).

QUANTIFICATION AND STATISTICAL ANALYSIS

Statistical tests in GraphPad Prism are indicated in the figure legends and were selected depending on whether data was normally distributed as assessed by the D'Agostino & Pearson omnibus normality test in Prism. Equal variance was assessed by an F-test in Prism and non-parametric tests used if this test was failed. Where necessary AUC's were calculated using Prism to allow analysis of longitudinal data. Number of animals and cellular assay replicates are indicated in the figure legends.

Western blot bands were analyzed and quantified using ImageJ (Schneider et al., 2012).

Energy Expenditure adjustment for lean mass by ANCOVA using SPSS.

Cell Reports, Volume *25*

Supplemental Information

A Wars2 Mutant Mouse Model Displays OXPHOS Deficiencies and Activation of Tissue-Specific Stress Response Pathways

Thomas Agnew, Michelle Goldsworthy, Carlos Aguilar, Anna Morgan, Michelle Simon, Helen Hilton, Chris Esapa, Yixing Wu, Heather Cater, Liz Bentley, Cheryl Scudamore, Joanna Poulton, Karl J. Morten, Kyle Thompson, Langping He, Steve D.M. Brown, Robert W. Taylor, Michael R. Bowl, and Roger D. Cox

Supplementary Information

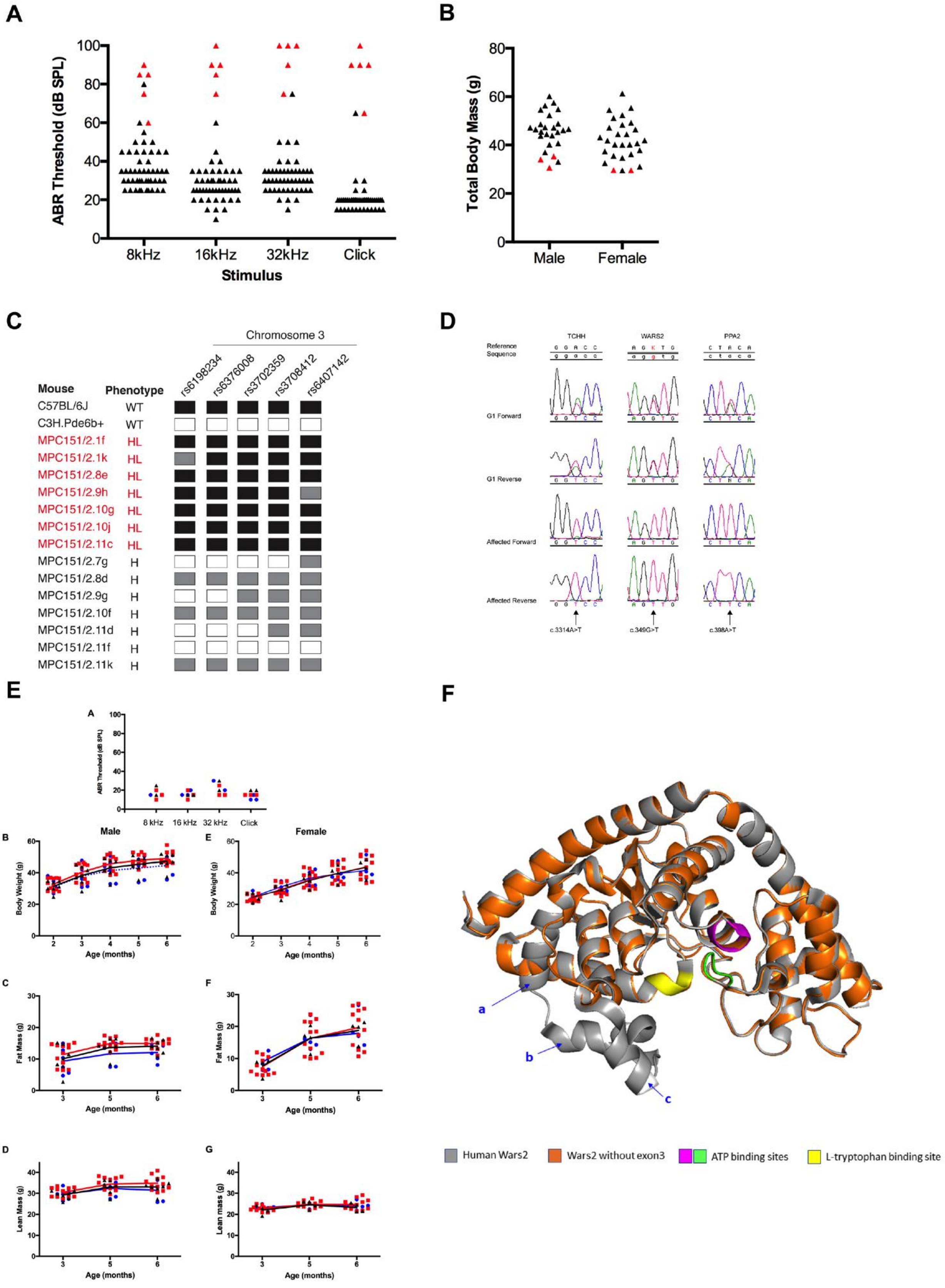

Supplementary Figure 1. Related to Figure 1. Auditory phenotyping, SNP mapping, whole genome sequencing of mouse pedigree MPC151, non-segregation of the phenotype in PP2A mice and the crystal structure of WARS2.

A) Auditory brainstem response (ABR) phenotyping of pedigree MPC151 at 12-months of age showed 5 mice with elevated hearing thresholds (red triangles) at all the frequencies tested (8, 16 and 32 kHz) and for the click stimulus, compared to their normal-hearing littermates (n=48, black triangles).

B) Body weight phenotyping of male and female pedigree MPC-151 at 12-months of age shows that 5 mice with elevated hearing thresholds (male n=3, female n=2, red triangles) also exhibit low body weight, compared to normal-hearing littermates (male n=23, female n=26, black triangles).

C) DNA from the 7 mice exhibiting hearing loss (HL) at 9-months of age and 7 normal-hearing (H) littermates was analyzed by whole genome SNP mapping. The first column indicates the mouse identification numbers and the second column their respective phenotype. The genotype of each mouse is either homozygous for C57BL/6J (black) or C3H (white) or heterozygous (grey) for each marker. The analysis defined a ~73.3Mb critical interval on Chromosome 3 between markers rs6198234 and rs6407142 (Chr3:70361430-143619317, GRCm38).

D) Sanger sequencing of MPC-151 G1 founder and MPC-151 'affected' G3 (MPC151/2.10g) at the shown locations corresponding to the three identified ENU-induced missense mutations on Chr 3: *Tchh*-c.3314A>T, *Wars2*-c.349G>T and *Ppa2*-c.398A>T. The MPC-151 G1 founder is heterozygous for all 3 missense mutations. The MPC-151 'affected' G3 is homozygous for the three missense mutations.

E) Intercross cohort mice segregating the PPA2 Y123F mutation do not exhibit a phenotype. In subpanels (A) ABR at 6 months of age and (D-G) phenotyping over a 6 month time-course in male and female mice respectively for (B,E) body weight, (C,F) fat mass and (D,G) lean mass. In subpanel (A) PPA2$^{Y123F/Y123F}$ n=4, PPA2$^{Y123F/+}$ n=3, PP2A$^{+/+}$ n=2, and in subpanels (B-G) male and female for PPA2$^{Y123F/Y123F}$ n=6 and n=5, for PPA2$^{Y123F/+}$ n=12 and n=12 and for PP2A$^{+/+}$ n=7 and n=4 respectively. Time course data were analyzed with a 2-way ANOVA and Bonferroni correction for multiple testing and all comparisons were non-significant. Homozygous PPA2$^{Y123F/Y123F}$ are blue circles, heterozygous PPA2$^{Y123F/+}$ are red squares and wildtype colony-mates are black triangles.

F) The crystal structure of Human WARS2 protein (pdb id: 5ekd) colored grey aligned to the predicted protein structure of WARS2 *without* exon 3 colored orange. The structural prediction of the deletion of exon 3 shows that three α -helices (a, b & c) are removed in the resulting predicted Wars2 protein structure. The two exon 3 encoded alpha helices are a and b. The catalytic domain, which comprises a Rossmann fold, holds three active sites; Class I PxxxxHIGH and KMSKS active-site catalytic (ATP binding) motifs, colored magenta and green respectively, and the L-tryptophan binding site, in yellow. The catalytic motifs are not directly affected by the mutation. However, these three 3 α –helices are part of the AARs catalytic domain which synthesizes aminoacyl adenylate and moves the amino acid to the anticodon binding domain (not shown).

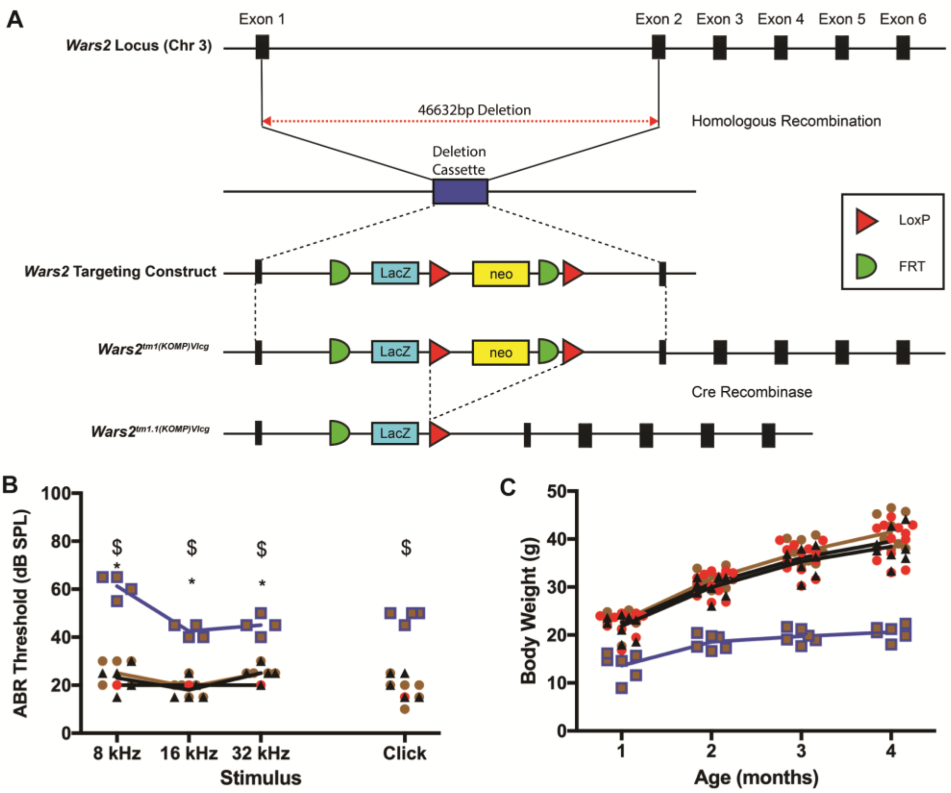

	1 month	2 month	3 month	4 month
Wars2$^{+/+}$ to *Wars2*$^{V117L/-}$	0.0519	0.0554	0.0387	0.0728
Wars2$^{V117L/+}$ to *Wars2*$^{V117L/-}$	0.0026	0.0030	0.0041	0.0043
Wars2$^{+/-}$ to *Wars2*$^{V117L/-}$	0.0016	0.0011	0.0011	0.0004

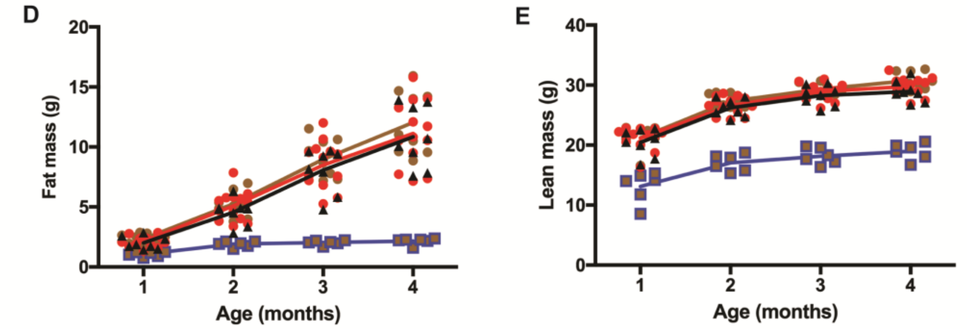

	1 month	2 month	3 month	4 month
Wars2$^{+/+}$ to *Wars2*$^{V117L/-}$	0.053	0.0691	0.0224	0.0285
Wars2$^{V117L/+}$ to *Wars2*$^{V117L/-}$	0.0050	0.0022	0.0041	0.0051
Wars2$^{+/-}$ to *Wars2*$^{V117L/-}$	0.0005	0.0013	0.0021	0.0012

	1 month	2 month	3 month	4 month
Wars2$^{+/+}$ to *Wars2*$^{V117L/-}$	0.0349	0.0649	0.0649	0.1589
Wars2$^{V117L/+}$ to *Wars2*$^{V117L/-}$	0.0022	0.0039	0.0021	0.0027
Wars2$^{+/-}$ to *Wars2*$^{V117L/-}$	0.0031	0.0006	0.0016	0.0003

Supplementary Figure 2. Related to Figure 1. ***Wars2$^{V117L/-}$*** **compound heterozygous knockout phenotyping data.**

A) Schematic diagram illustrating the generation of the KOMP *Wars2*-KO allele (*Wars2$^{tm1(KOMP)Vlcg}$*). The targeting construct integrated into the C57BL/6N ES cell genome by homologous recombination, deleting 46632bp of the *Wars2* gene locus, including coding regions of both *Wars2*-Exon1 and *Wars2*-Exon2, leading to a frame-shift and a premature stop codon. *Wars2$^{tm1(KOMP)Vlcg}$* ES cells were micro-injected into C57BL/6N blastocysts generating mosaic C57BL/6N-*Wars2$^{tm1(KOMP)Vlcg}$* offspring. Germ-line transmission (GLT) of the *Wars2$^{tm1(KOMP)Vlcg}$* construct was determined by genotyping C57BL/6N-*Wars2$^{tm1(KOMP)Vlcg}$* x C57BL/6N offspring for the neomycin selection cassette (data not shown). Once GLT was achieved C57BL/6N-*Wars2$^{tm1(KOMP)Vlcg}$* mice were crossed with cre-recombinase expressing mice to remove the neomycin selection cassette. *Wars2$^{+/-}$* indicates heterozygous mice with one wildtype allele (+) and one deleted allele (-).

B) Auditory brainstem response thresholds at 4-months of age were recorded at single frequencies: 8, 16 and 32kHz, and a click stimulus. *Wars2$^{+/+}$*,*Wars2$^{V117L/+}$* (*Tchh$^{D1105V/+}$*), *Wars2$^{+/-}$* and *Wars2$^{V117L/-}$* (*Tchh$^{D1105V/+}$*) animal numbers 5, 1, 6 and 4 respectively. Data were analyzed using a 1-way ANOVA non-parametric Kruskal-Wallis test and Dunns multiple comparison test between *Wars2$^{+/+}$* compared to *Wars2$^{V117L/-}$* (*Tchh$^{D1105V/+}$*) and *Wars2$^{+/-}$* compared to *Wars2$^{V117L/-}$*, (*Tchh$^{D1105V/+}$*) shown as * or $ $P<0.05$ respectively. Wildtype colony-mate *Wars2$^{+/+}$* black triangles, heterozygote point-mutation *Wars2$^{V117L/+}$*(*Tchh$^{D1105V/+}$*) red filled circles, heterozygous knockout *Wars2$^{+/-}$* brown filled circle and compound heterozygote *Wars$^{V117L/-}$* blue square filled with brown.

C) Body weight, **D)** Fat mass, **E)** Lean Mass were recorded from male mice 1- to 4-months of age. *Wars2$^{+/+}$*,*Wars2$^{V117L/+}$* (*Tchh$^{D1105V/+}$*), *Wars2$^{+/-}$* and *Wars2$^{V117L/-}$* (*Tchh$^{D1105V/+}$*) animal numbers 8, 13, n=9 and 6 respectively. AUCs were calculated baselined to zero and *Wars2$^{+/+}$*compared to *Wars2$^{V117L/-}$*, *Wars2$^{+/V117L}$* compared to *Wars2$^{V117L/-}$* (*Tchh$^{D1105V/+}$*) and *Wars2$^{+/-}$* compared to *Wars2$^{V117L/-}$* (*Tchh$^{D1105V/+}$*), using a 1-way ANOVA non-parametric Kruskal-Wallis test and Dunns multiple comparison test giving p values of 0.0691, 0.0038 and 0.006 respectively. Significance at specific time-points was calculated with a 1-way ANOVA non-parametric Kruskal-Wallis test and Dunn's multiple comparison test and is shown in table below each figure. Wildtype colony-mate *Wars2$^{+/+}$* black triangles, heterozygote point-mutation *Wars2$^{V117L/+}$*(*Tchh$^{D1105V/+}$*) red filled circles, heterozygous knockout *Wars2$^{+/-}$* brown filled circle and compound heterozygote *Wars$^{V117L/-}$* blue square filled with brown.

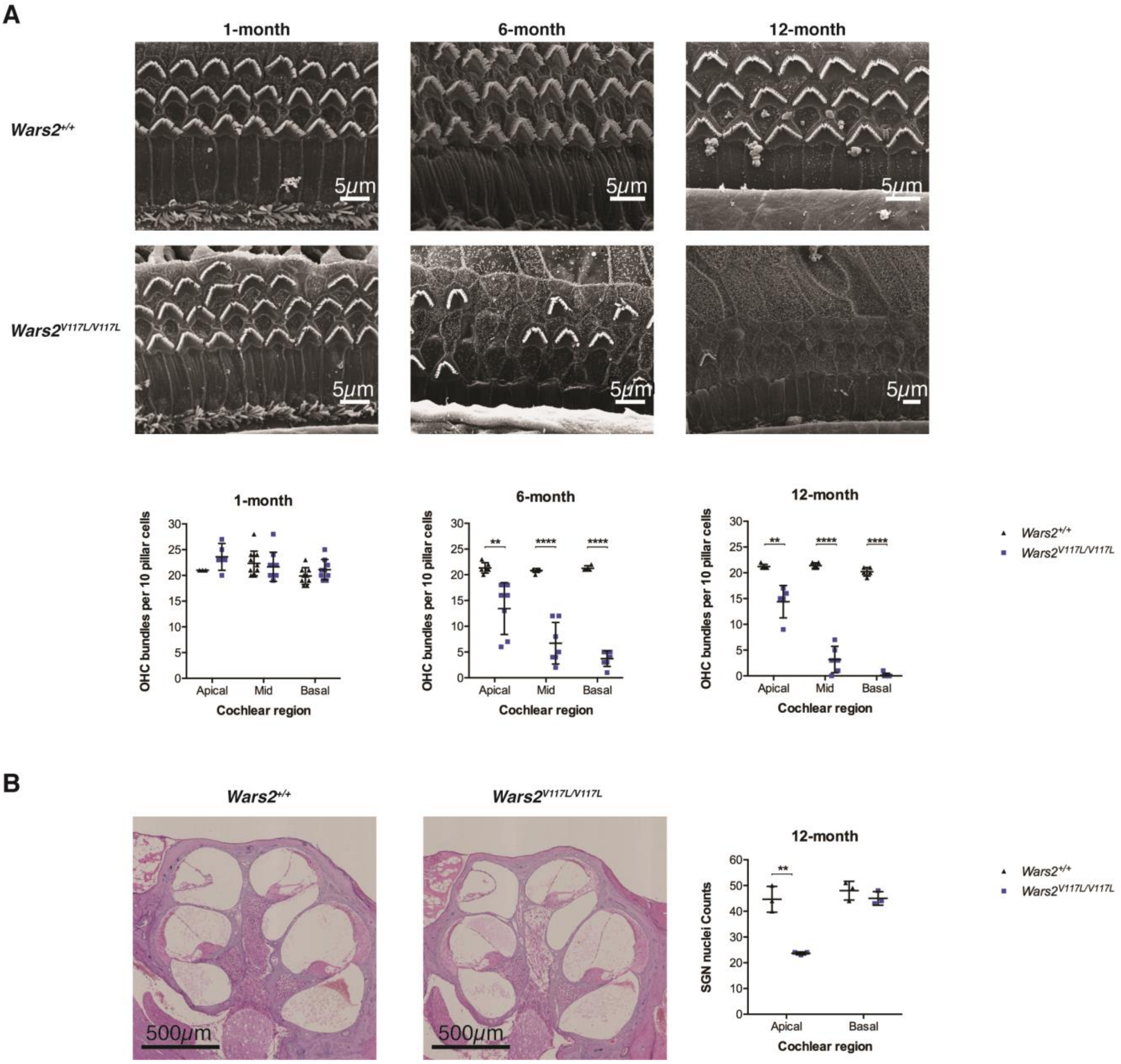
A
1-month
6-month
12-month
Wars2+/+
Wars2V117L/V117L
5μm
OHC bundles per 10 pillar cells
Apical
Mid
Basal
Cochlear region
**
****
B
Wars2+/+
Wars2V117L/V117L
500μm
12-month
SGN nuclei Counts
Apical
Basal
Cochlear region
**

Supplementary Figure 3. Related to Figure 1. Ultrastructural analyses reveal progressive loss of outer hair cell bundles, and histology shows reduced spiral ganglion neuron number, in *Wars2*$^{V117L/V117L}$ mutant mice.

A) Scanning Electron Micrographs of the mid-coil of the cochlear sensory epithelium from *Wars2*$^{+/+}$ and *Wars2*$^{V117L/V117L}$ mice at 1-, 6-, and 12-months of age. At 1-month of age, the number and appearance of the outer hair cell stereocilia bundles are as expected and similar across genotypes. At 6-months, there is loss of outer hair cell bundles in the *Wars2*$^{V117L/V117L}$ mutant mice, which is not observed in the *Wars2*$^{+/+}$ control mice. By 12-months of age, there is a near complete loss of outer hair cell bundles in the *Wars2*$^{V117L/V117L}$ mutant mice, which is not observed in the *Wars2*$^{+/+}$ control mice. Shown are representative images from the mid region of the cochlear spiral, at least three cochleae from independent mice were imaged per region for each genotype. Scale bar 5µm. To assess the loss of outer hair cell bundles in the apical, mid and basal turns of the cochlear coil counts were undertaken to determine the number of bundles adjacent to ten pillar cells. At 1-month of age *Wars2*$^{+/+}$ (apex n=3, Mid n=12, base n=9) and *Wars2*$^{V117L/V117L}$ (apex n=5, Mid n=9, base n=9) mice have similar numbers of OHC bundles. However, by 6-months of age *Wars2*$^{V117L/V117L}$ (apex n=7, Mid n=7, base n=7) mice have a reduced number of OHC bundles in all cochlear regions compared to *Wars2*$^{+/+}$ (apex n=6, Mid n=6, base n=4) mice. At 12-months of age Wars2$^{V117L/V117L}$ (apex n=5, Mid n=6, base n=7) mice show a further loss of OHC bundles in all cochlear regions compared to *Wars2*$^{+/+}$ (apex n=5, Mid n=5, base n=5) mice. While *Wars2*$^{V117L/V117L}$ mice show a progressive loss of OHC bundles throughout the cochlear spiral, no significant OHC bundle loss is observed in the *Wars2*$^{+/+}$ mice up to 12-months of age. Mean ± SD. Homozygous *Wars2*$^{V117L/V117L}$ are blue squares and *Wars2*$^{+/+}$ black triangles.

B) H&E-stained mid-modiolar cochlear sections from *Wars2*$^{+/+}$ and *Wars2*$^{V117L/V117L}$ mice at 12-months of age. Visual assessment of the sections shows the number of SGN nuclei to be similar from base-to-apex in *Wars2*$^{+/+}$ mice, but there appears fewer nuclei from base-to-apex in *Wars2*$^{V117L/V117L}$ mice. Shown are representative sections from one *Wars2*$^{+/+}$ and one *Wars2*$^{V117L/V117L}$ mouse. Scale bar 500µm. To assess this apparent reduced number of nuclei, counts were undertaken to determine the number of spiral ganglion neuron nuclei within a set 5000µm2 area within the apical and basal turns.This shows that in the basal turn *Wars2*$^{+/+}$ and *Wars2*$^{V117L/V117L}$ mice have comparable numbers of nuclei. In addition, *Wars2*$^{+/+}$ mice have comparable numbers of nuclei in the apical and basal turns. However, *Wars2*$^{V117L/V117L}$ mice do not have comparable numbers of nuclei in the apical and basal turns. Three sections per genotype were used for counts, obtained from independent mice, mean ± SD. Homozygous *Wars2*$^{V117L/V117L}$ are blue squares and *Wars2*$^{+/+}$ black triangles. Significance was determined using an unpaired t test: ** $P<0.01$, *** $P<0.001$, **** $P<0.0001$.

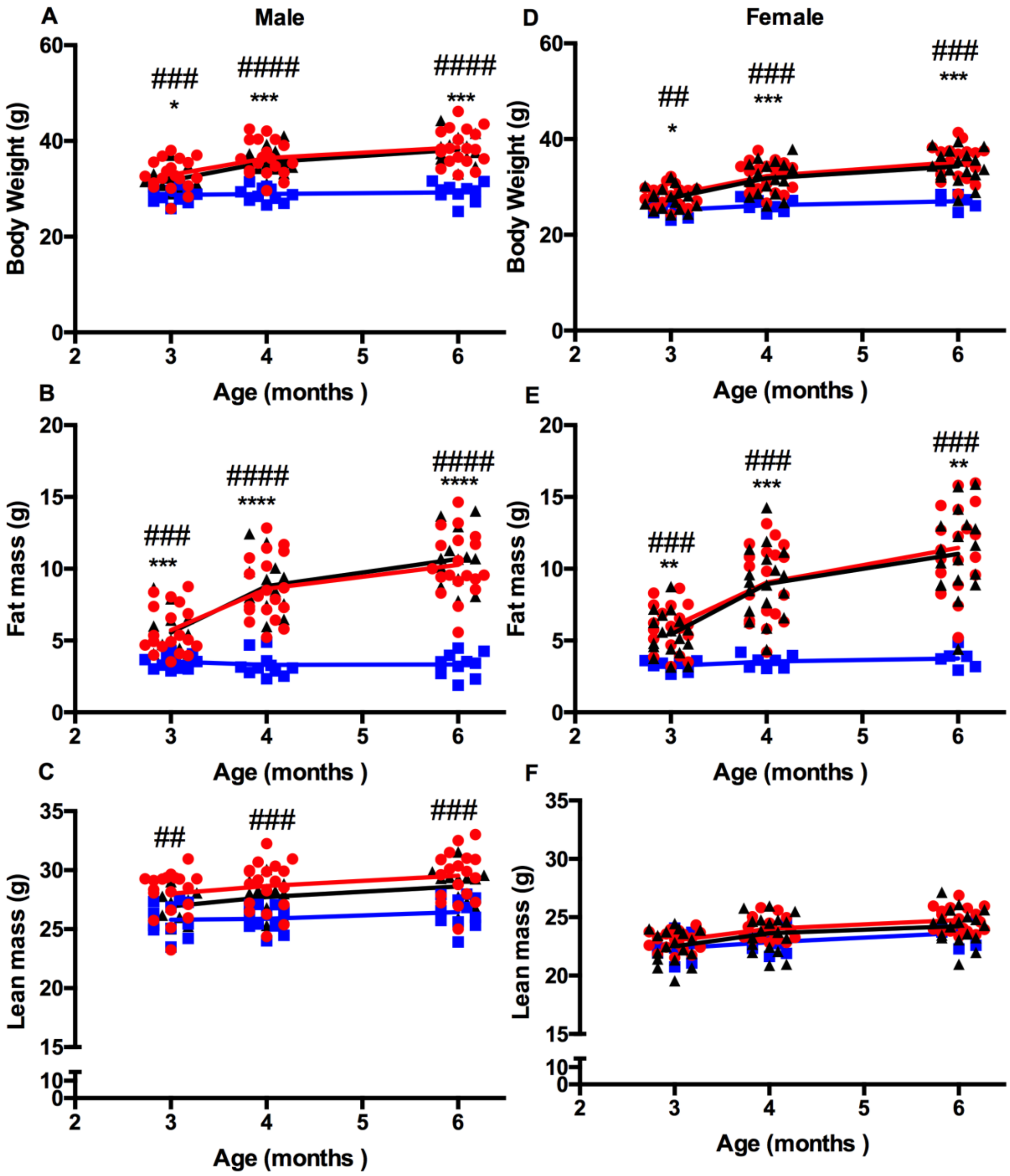

A
Male
Body Weight (g)
60
40
20
0
2
3
4
5
6
Age (months)
###
*
####
***
####
***
D
Female
##
*
###
***
###
***
B
Fat mass (g)
20
15
10
5
###
***
####
****
####
****
E
###
**
###
***
###
**
C
Lean mass (g)
35
30
25
15
10
##
###
###
F

Supplementary Figure 4. Related to Figure 2. ***Wars2***$^{V117L/V117L}$ **mice have reduced body weight due to reduced adiposity.** Male and female **A and D)** Body weight **B and E)** Fat mass and **C and F)** Lean mass in cohort 2 mice. *Wars2*$^{V117L/V117L}$,*Wars2*$^{V117L/+}$*, Wars2*$^{+/+}$ animal numbers, male and female, were 11 and 6-7, 19 and 19 and 13 and 18, respectively. AUC calculated with zero baselines and compared using a 1-way ANOVA non-parametric Kruskal-Wallis test and Dunns multiple comparison test. For AUC comparing *Wars2*$^{+/+}$ and *Wars2*$^{V117L/V117L}$, *Wars2*$^{V117L/+}$ and *Wars2*$^{V117L/V117L}$*,* and *Wars2*$^{+/+}$ and *Wars2*$^{V117L/+}$: Male body weight 0.0003, <0.0001 and >0.999; fat mass <0.0001, <0.0001 and >0.999; lean mass 0.0847, 0.0005 and 0.4131 respectively; Female body weight 0.0020, 0.0005 and >0.999; fat mass 0.0016, 0.0005 and >0.999; lean mass 0.8539, 0.2825 and >0.999 respectively. Significance at specific time-points was also calculated using a 1-way ANOVA non-parametric Kruskal-Wallis test and Dunns multiple comparison test, significance between *Wars2*$^{V117L/V117L}$ and *Wars2*$^{+/+}$ shown as * P<0.05, ** P<0.01, *** P<0.001, **** P<0.0001 and significant differences between *Wars2*$^{V117L/V117L}$ and *Wars2* $^{V117L/+}$shown as # P<0.05, ## P<0.01, ### P<0.001, #### P<0.0001. Homozygous *Wars2*$^{V117L/V117L}$ are blue squares, heterozygous *Wars2*$^{V117L/+}$ are red circles and wildtype colony-mate *Wars2*$^{+/+}$ black triangles.

Quantification of Western blots at 12 months of age Figure 4 D to J

A

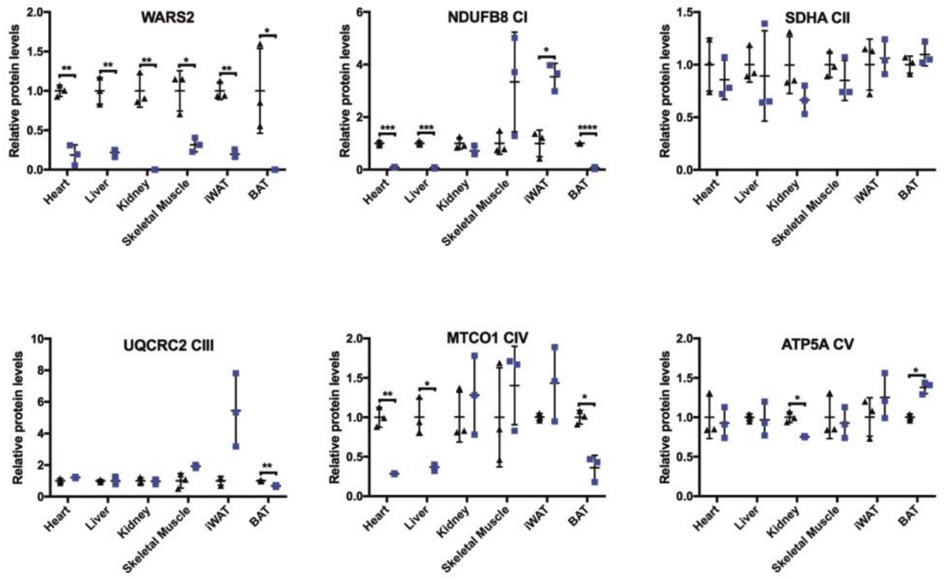

Respiratory chain complex activities

B C

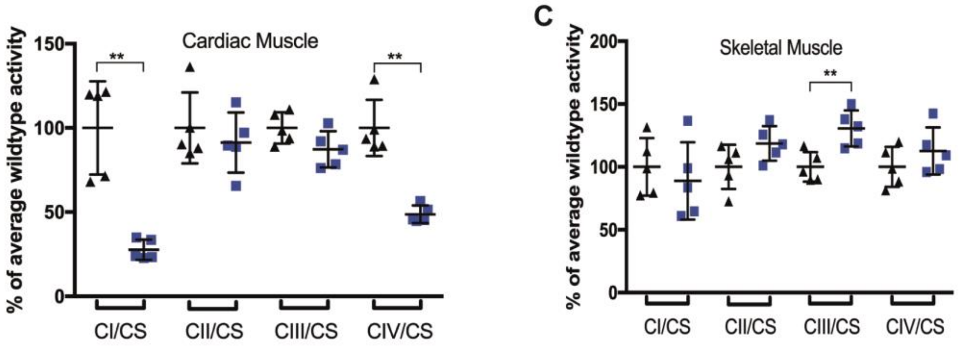

Brain Western blots 3-5 months of age

D E F

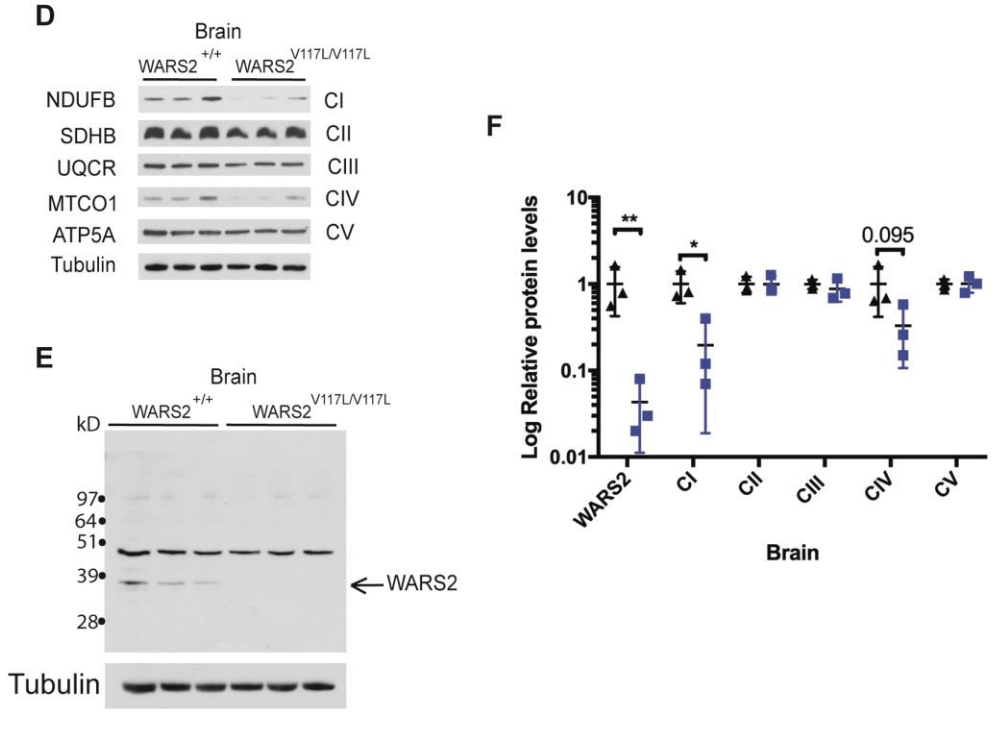

Supplementary Figure 5. Related to Figure 4. (A) Quantification of Western blots from Figures 4 D to J. (B and C) Reduced complex I and IV activities in cardiac muscle and increased CIII activity in skeletal muscle of *Wars2*$^{V117L/V117L}$ mice by 12 months of age. (D, E and F) Reduced WARS2 and complex I and IV deficiencies in whole brain of mice at 3-5 months of age.

A) Quantification of 12 month of age WARS2 and respiratory chain complex subunits in multiple tissues shown in main **Figure 4** western blots. *Wars2*$^{V117L/V117L}$ and *Wars2*$^{+/+}$ animal numbers were 3 each. Statistical analysis was done by multiple t-tests using the Holm-Sidak method and without assuming consistent standard deviation. Adjusted p values are shown * $P<0.05$, ** $P<0.01$, *** $P<0.001$, **** $P<0.0001$.

B and C) Quantified respiratory chain complex activities in **B)** cardiac muscle and **C)** skeletal muscle, normalized to citrate synthase activity and expressed as a percentage of average wildtype values**.** *Wars2*$^{+/+}$ and *Wars2*$^{V117L/V117L}$ animal numbers 5 each. Cardiac muscle CI and CIV data was analyzed using a Mann-Whitney t-test and all other data with an unpaired two-tailed t-test.

D) Immunoblot analysis of WARS2 and **E)** mitochondrial respiratory chain sub-unit protein levels protein in *Wars2*$^{V117L/V117L}$ *Wars2*$^{+/+}$ (C3H/Pde) whole brain samples from male mice aged between approximately 3 and 5 months of age. *Wars2*$^{V117L/V117L}$ and *Wars2*$^{+/+}$ animal numbers were 3 each. **F)** Quantification of protein blots in **D** and **E**, data plotted as Log10 relative to widltype. For statistical analysis raw data was square root transformed and analyzed by an unpaired t-test for each probe. * $P<0.05$, ** $P<0.01$. Homozygous *Wars2*$^{V117L/V117L}$ are shown as blue squares and wildtype colony-mate *Wars2*$^{+/+}$ as black triangles.

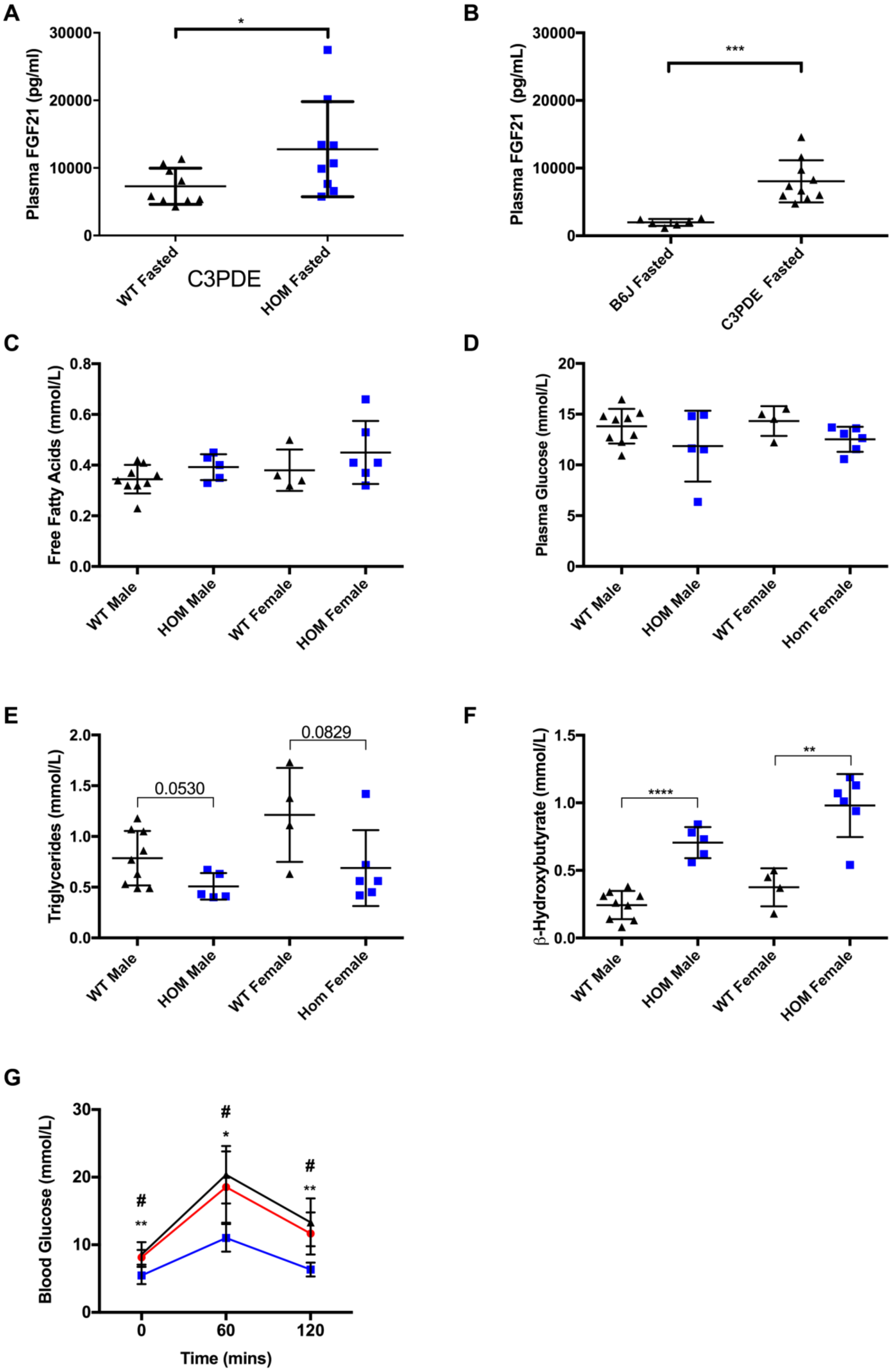
A
Plasma FGF21 (pg/ml)
30000
20000
10000
0
*
WT Fasted
HOM Fasted
C3PDE
B
Plasma FGF21 (pg/mL)
***
B6J Fasted
C3PDE Fasted
C
Free Fatty Acids (mmol/L)
0.8
0.6
0.4
0.2
0.0
WT Male
HOM Male
WT Female
HOM Female
D
Plasma Glucose (mmol/L)
20
15
10
5
0
WT Male
HOM Male
WT Female
Hom Female
E
Triglycerides (mmol/L)
2.0
1.5
1.0
0.5
0.0
0.0530
0.0829
WT Male
HOM Male
WT Female
Hom Female
F
β-Hydroxybutyrate (mmol/L)
1.5
1.0
0.5
0.0
****
**
WT Male
HOM Male
WT Female
HOM Female
G
Blood Glucose (mmol/L)
30
20
10
0
#
**
#
*
#
**
0
60
120
Time (mins)

Supplementary Figure 6. Related to Figure 7. Increased plasma FGF21 is linked with systemic changes in metabolism in *Wars2$^{V117L/V117L}$* mice.

A and B) Plasma FGF21. A) Fasted plasma FGF21 in 4-month old overnight fasted male mice. *Wars2$^{V117L/V117L}$* and *Wars2$^{+/+}$* animal numbers were 9 each, mean ± SD. **B)** Fasted plasma FGF21 in 3-4-month old overnight fasted male mice C57BL/6J (B6J) and C3H/Pde (C3PDE) B6J and C3PDE animal numbers were 6 and 10 respectively, mean ± SD. Significance in **A)** and **B)** calculated using a Mann Whitney 2-tailed t-test, * <0.05 and *** <0.001. Note that the C3PDE background on which the *Wars2^{V117L}* mutation is maintained shows strain specific differences in FGF21 concentration. Homozygous *Wars2$^{V117L/V117L}$* are blue squares and wildtype colony-mate *Wars2$^{+/+}$* black triangles. Significant differences between groups shown as * P<0.05, *** P<0.001.

C, D, E and F) plasma clinical chemistry. C) Free fatty acids (FFA), **D)** glucose, **E)** triacylglycerides (TAG) and **F)** β-hydroxybutyrate levels were analysed in plasma samples collected from *Wars2$^{V117L/V117L}$* and *Wars2$^{+/+}$* mice at 12-months of age. *Wars2$^{V117L/V117L}$* and *Wars2$^{+/+}$* male and female animal numbers were 5, 6 and 9,4 respectively, mean ± SD. Data A and B analysed by Mann-Whitney test and data C and D by unpaired two-tailed t-test. Homozygous *Wars2$^{V117L/V117L}$* are blue squares and wildtype colony-mate *Wars2$^{+/+}$* black triangles. Significant differences between groups shown as * P<0.05, **** P<0.0001

G) Intraperitoneal glucose tolerance tests (IPGTT) were performed in male *Wars2$^{V117L/V117L}$*, *Wars2$^{V117L/+}$* and *Wars2$^{+/+}$* mice at 6-months of age. *Wars2$^{V117L/V117L}$*, *Wars2$^{V117L/+}$* and *Wars2$^{+/+}$* animal numbers were 5,7 and 5 respectively, mean ± SD. IPGTT were performed early in the morning following over-night fasting and tail blood glucose levels were taken 0, 60 and 120 mins after an intraperitoneal glucose injection. AUC was calculated with zero baselines and compared using a one-way ANOVA with Tukey's multiple comparison test. For AUC comparisons between *Wars2$^{+/+}$* and *Wars2$^{V117L/V117L}$*, *Wars2$^{V117L/+}$* and *Wars2$^{V117L/V117L}$, Wars2$^{+/+}$* and *Wars2$^{V117L/+}$,* 0.0143, 0.0474 and 0.6545. Significance at specific time-points was calculated with a 1-way ANOVA with Tukey's multiple comparison test. Significance between *Wars2$^{V117L/V117L}$* and *Wars2$^{+/+}$* shown as * P<0.05, ** P<0.01, and significant differences between *Wars2$^{V117L/V117L}$* and *Wars2$^{V117L/+}$* shown as # P<0.05, ## P<0.01. Homozygous *Wars2$^{V117L/V117L}$* are blue squares, heterozygous *Wars2$^{V117L/+}$* are red circles and wildtype colony-mate *Wars2$^{+/+}$* black triangles.

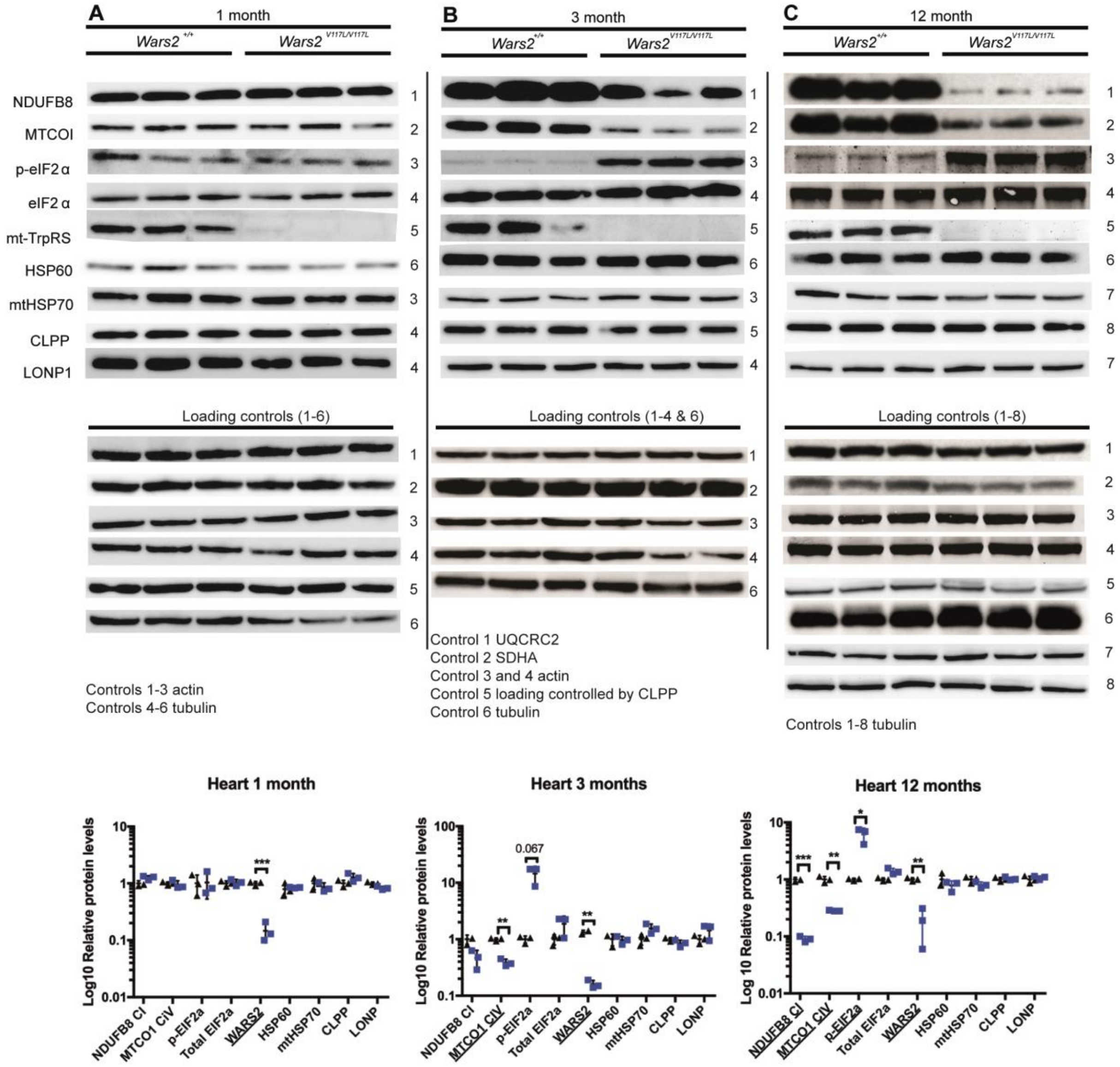
A
1 month
Wars2 +/+
Wars2 V117L/V117L
NDUFB8
MTCOI
p-eIF2α
eIF2α
mt-TrpRS
HSP60
mtHSP70
CLPP
LONP1
Loading controls (1-6)
Controls 1-3 actin
Controls 4-6 tubulin
B
3 month
Loading controls (1-4 & 6)
Control 1 UQCRC2
Control 2 SDHA
Control 3 and 4 actin
Control 5 loading controlled by CLPP
Control 6 tubulin
C
12 month
Loading controls (1-8)
Controls 1-8 tubulin
Heart 1 month
Heart 3 months
Heart 12 months
Log10 Relative protein levels
Log 10 Relative protein levels
0.067
NDUFB8 CI
MTCO1 CIV
p-EIF2a
Total EIF2a
WARS2
HSP60
mtHSP70
CLPP
LONP

Supplementary Figure 7. Related to Figure 7. Activation of the ISR is progressive with age, co-incident with mitochondrial respiratory chain deficiencies and independent of disrupted mitochondrial proteostasis in *Wars2$^{V117L/V117L}$* heart. Immunoblot analysis of LONP1, CLPP, mtHsp70, mtHsp60, p-eIF2α, (total)eIF2α, COXI (CIV) and NDUFB8 (CI) protein levels in *Wars2$^{V117L/V117L}$* and *Wars2$^{+/+}$* heart tissue collected from female mice at **A)** 1-month, **B)** 3-months and **C)** 12-months of age. *Wars2$^{V117L/V117L}$* and *Wars2$^{+/+}$* animal numbers were 3 each. Note that some 12-month blots are reproduced here from display figures for ease of comparison. The controls for each gel are indicate by numbers on the right of each panel and then shown in the loading control below each panel, note that some blots are re-probed and therefore share controls. Quantification plots are shown directly below the corresponding western blot panels. Statistical analysis was done by multiple t-test (PRISM) using the Holm-Sidak method and without assuming consistent standard deviation. Adjusted p values are shown * $P<0.05$, ** $P<0.01$, *** $P<0.001$. Homozygous *Wars2$^{V117L/V117L}$* are shown as blue squares and wildtype colony-mate *Wars2$^{+/+}$* as black triangles.

Supplementary Table 1, Related to Figure 2.

***Wars2$^{-/-}$* causes embryonic lethality.** Number of mice born per genotype from three inter-crosses: **A)** *Wars2$^{V117L/+}$* x *Wars2$^{+/-}$*, **B)** *Wars2$^{V117L/+}$* x *Wars2$^{V117L/+}$* and **C)** *Wars2$^{+/-}$* x *Wars2$^{+/-}$*. Data were analysed for deviance from expected Hardy-Weinberg ratios using a Chi-squared test.

**A) Inter-cross of *Wars2$^{V117L/+}$* x *Wars2$^{+/-}$* mice**		
**Genotype**	**Expected**	**Observed**
Wars2$^{+/+}$	19.5	23
Wars$^{V117L/+}$	19.5	26
Wars2$^{+/-}$	19.5	21
Wars$^{V117L/-}$	19.5	8
Total	78	78

Chi-squared test p=0.1103

**B) Inter-cross of *Wars2$^{V117L/+}$* x *Wars2$^{V117L/+}$* mice**		
**Genotype**	**Expected**	**Observed**
Wars2$^{+/+}$	51.5	56
Wars$^{V117L/+}$	103	101
Wars$^{V117L/V117L}$	51.5	49
Total	*206*	*206*

Chi-squared test p=0.8647

**C) Inter-cross of *Wars2$^{+/-}$* x *Wars2$^{+/-}$* mice**		
**Genotype**	**Expected**	**Observed**
Wars2$^{+/+}$	44.5	53
Wars$^{+/-}$	89	125
Wars$^{-/-}$	44.5	**0**
Total	*178*	*178*

Chi-squared test p=<0.0001

Supplementary Table 2, Related to Star Methods

Oligonucleotide primer sequences

**Primers for genotyping**	
**Name**	**Sequence**
CTCAATCCCATTAAGCAAGATAT	Ppa2^{A398T} primers: biotinylated forward
GGTTTCTGTAGAAGGCATAAAAG	Ppa2^{A398T} primers: biotinylated reverse
GGGAAGATGTTCGGTG	Ppa2^{A398T} primers: sequencing reverse
GGTCACCTTTCTTTCTCTCC	Wars2^{G349T} primers: forward
CAGGTGAGGATCCAACTTAA	Wars2^{G349T} primers: reverse
TTTCTCTCCTTCCTTTTAG	Wars2^{G349T} primers: forward sequencing
TCAGCCTATCCCTGTTGTCTA	*Wars2*^{V117L} Primers: forward
TGGTGTAAATGCTGCAATCG	*Wars2*^{V117L} Primers: reverse
CCTTCCTTTTAGTTGTCTGAACACACTCAG	*Wars2*^{V117L} Primers: probe
GCCCAGCACTTGGGATGT	Wars2^{WT} primers: forward
GCAGCCAGCTCACCAATG	Wars2^{WT} primers: reverse
TCCCTTCACTTTCCTGTCTCCGTTTC	Wars2^{WT} primers: FAM labeled probe
CTCGCCACTTCAACATCAAC	LacZ primers: forward
TTATCAGCCGGAAAACCTACC	LacZ primers: reverse
TCGCCATTTGACCACTACCATCAATCC	LacZ primers: FAM labelled probe
GCCCCAGCACGACCATT	Dot1l primers: forward
TAGTTGGCATCCTTATGCTTCATC	Dot1l primers: reverse
CCAGCTCTCAAGTCG	Dot1l primers: VIC labelled probe
